# An open-source workflow for identifying hydrodynamic water quality events in rivers by continuous water quality monitoring and time-series data processing using R and US EPA CANARY^☆^

**DOI:** 10.1016/j.mex.2025.103538

**Published:** 2025-07-27

**Authors:** L. Cronin, C.M. Taylor, C. Briciu Burghina, F.E. Lucy, F. Regan

**Affiliations:** a School of Chemical Sciences, Glasnevin, Dublin 9, Ireland; b DCU Water Institute, Glasnevin, Dublin 9, Ireland; c Centre for Environmental Research, Innovation and Sustainability CERIS, Dept. of Environmental Science, Atlantic Technological University, Sligo Campus, F91 YW50, Ireland

**Keywords:** Event detection, Water quality time series, US EPA CANARY, Hydrodynamic water quality events, Event driven pollution, Diffuse pollution, Continuous monitoring, High frequency monitoring, Water quality events workflow, EU water framework directive

## Abstract

Improving European surface water quality requires urgent action to address diffuse pollution sources particularly from agriculture, with increased frequency and intensity of hydroclimatic events also a key driver of pollutant export to waters and water quality decline worldwide.

However, the need for comprehensive, practical protocols for sensor deployment, sensor maintenance and data management for the adoption of high frequency water quality monitoring has been highlighted, along with the challenges for citizen scientists in analyzing millions of water quality data points and sharing metadata. The practical method presented, with reproducibility built into the workflow, is designed for multiple users and a step-by-step application of the workflow is demonstrated including:

• Deployment arrangement for water quality sondes in two temporary monitoring stations with different site characteristics.
• Data collection and data validation methods.
• Concise, reproducible, open-source workflow detailing the use of R, R markdown and US EPA CANARY software for data import, data cleaning, data visualization, data integrity, along with site-specific CANARY event system configuration for the detection of potential water quality events.

Results for two monitoring stations on different rivers show CANARY successfully identified 100 % (n 47) and 97 % (n 39) of the manually identified turbidity events.

## Graphical abstract

**Figure:**
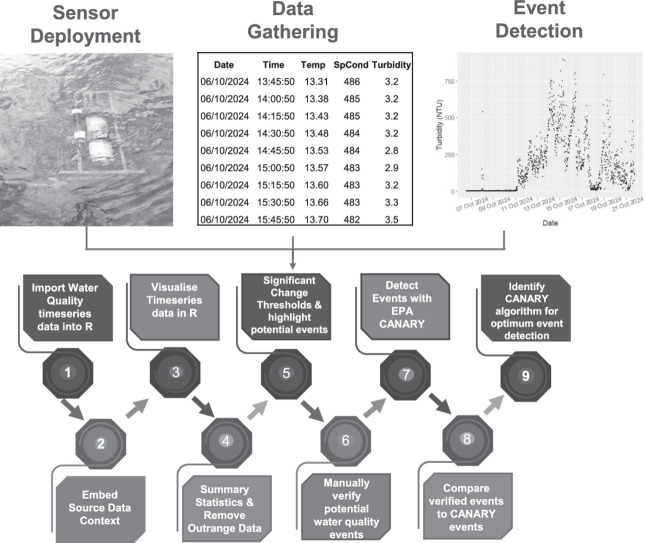

## Specifications table

**Table:** 

**Subject area**	Environmental Science
**More specific subject area**	*High frequency river monitoring for temperature, conductivity and turbidity to detect rainfall driven water quality events.*
**Name of your method**	*Analysis of time series water quality data using R and US EPA CANARY.*
**Name and reference of original method**	*Burkhardt, J.B., Sahoo, D., Hammond, B., Long, M., Haxton, T., Murray, R., 2022. Near real-time event detection for watershed monitoring with CANARY. Environ. Sci. Adv. 1, 170–181.*https://doi.org/10.1039/D2VA00014H
**Resource availability**	1. Water Quality sonde YSI 6600 EDS V2–2 software: Ecowatch Lite v1.0.5.15, YSI Inc., Ohio, USA, https://www.ysi.com/customer-support/software-firmware-downloads/software?srsltid=AfmBOoqzASTLYbXCJY4k7S-9Gk0-hOYObej76o4TtyAmE-GLfjbBjihm 2. USEPA CANARY-EDS Event Detection Software https://github.com/USEPA/CANARY 3. Statistical computing and graphics: • R Statistical language v4.4.1 (R Core Team, 2024) • R Studio v2024.04.2+764 (Posit team, 2024) • Packages in R: cowplot (Wilke, 2024), data.table (Barrett et al., 2024), dplyr (Wickham et al., 2023), ggplot2 v3.5.2 (Wickham, 2009), grid (Murrell, 2005), gridExtra (Auguie and Antonov, 2017), here (Müller and Bryan, 2020), labelled (Larmarange et al., 2025), lubridate (Grolemund and Wickham, 2011), naniar (Tierney and Cook, 2023), plotly (Sievert, 2020), scales (Wickham, Pedersen, et al., 2024), stargazer (Hlavac, 2022), stringr (Wickham, Software and PBC, 2023), tidyr (Wickham, Vaughan, et al., 2024), tidyverse (Wickham et al., 2019). For all references listed above, see Supplementary References S5. 4. Application code, output files, R markdown and sample dataset can be found at: https://github.com/LCroninATU/Workflow-for-identifying-water-quality-hydrodynamic-events-in-time-series-data

## Background

Almost 25 years since the Water Framework Directive (WFD) (European Commission, 2000, see Supplementary References) was adopted, only 37 % of European surface waters have attained good or high ecological status and only 29 % have attained good chemical status (EEA, 2024; see Supplementary References). Diffuse sources of pollution, particularly those from agriculture, are one of the greatest obstacles to restoring water quality (Kristensen et al., 2018; see Supplementary References), with increased frequency and intensity of hydrodynamic events due to climate change a key driver of water quality decline [1].

Identifying effective restoration measures requires adequate data and high frequency monitoring to facilitate rapid, real time strategic adaptation for the delivery of water quality improvements [2] with more data required to determine specific water quality pressures [3,4]. Numerous studies have demonstrated the value of high frequency, continuous water quality monitoring for surface waters [[5], [6], [7], [8]]. High frequency water quality monitoring requires ‘robust deployment, maintenance, and data management protocols’ [9] along with ‘practical guidelines’ [10], and the challenges for citizen scientists in sharing metadata and analyzing millions of water quality data points has been highlighted [11]. Furthermore, many researchers are not software engineers or formally trained in coding [12] with code errors and the inability to reproduce analyses frequently encountered, even where datasets and code were published [13,14].

High frequency water quality monitoring is being carried out in six diverse catchments in Ireland [15] via permanent, long term, monitoring stations. One of the key attributes of this method is that no permanent infrastructure is required for the off-grid monitoring stations, with a focus on shorter term, temporary, remote deployments that can be moved between waterbodies. In dynamic environments such as river catchments, this flexibility allows for rapid response to changing conditions or seasonal deployment. The original method for this research filtered out rainfall events and provided little detail on the method for event matching with CANARY event detection software [16], both of which are included in this method.

The practical method presented here is designed for regulators, environmental agencies, hydrological agencies and researchers to facilitate the adoption of high frequency water quality monitoring. This method was developed based on the knowledge acquired through the deployment of high frequency water quality monitoring sondes, in two temporary, rural, river monitoring stations in the northwest of Ireland. The data was processed through US EPA CANARY (Hart and Haxon, 2020; see Supplementary References). The framework presented provides a concise, reproducible, open-source method. The workflow details the use of R (R Core Team, 2024; see Supplementary References)), each step in the data import, cleaning, and visualization process, in addition to site-specific US EPA CANARY event system configuration for the detection of potential water quality events.

To minimize the issues associated with non-reproducible code, this workflow incorporates best practice in data management, data stewardship and reproducible research. In addition to the data, output files, and R script, an R Markdown file for this project was published (Cronin and Taylor, 2025; see Supplementary References) which includes the code, the results, and explanatory text in a single interactive file to facilitate ease of reproducibility. The use of the open-source R software and US EPA CANARY event detection, the publication of data, of code and outputs of this research allows for the code to be adopted, modified, improved or forked, allowing for greater flexibility and customization by end users.

## Method details

### Study area

**Fig. 1:**
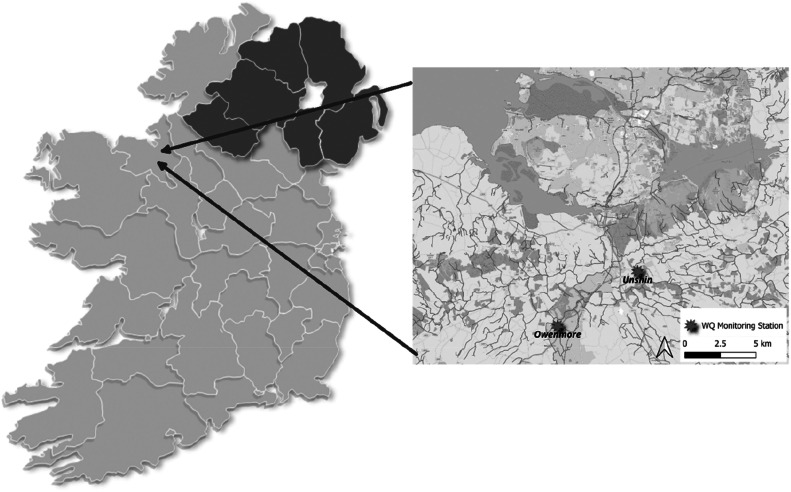
Location of the two continuous monitoring stations in the study area (Unshin & Owenmore rivers, Co. Sligo, Ireland).

The study sites were located in the northwest region of Ireland on two different rivers in the Sligo Bay catchment with one monitoring station on the River Owenmore and one station on the River Unshin. The Owenmore-Unshin catchment covers an area of 655 km^2^ with the Owenmore river running for circa 52 km and the River Unshin, a tributary of the River Owenmore, running for circa 23 km (Goodwillie, Buckley and Douglas, 1992; see Supplementary References)). The location of both monitoring stations at the study sites was influenced by a number of factors including adequate water flow and level, the close proximity of hydrometric stations, the availability of historical water quality data, permission by landowners to site monitoring equipment, and security of the monitoring equipment over the long deployment period (Fig. 1).

The Unshin river location was selected as an example of a river with a high-status objective under the WFD. The national water quality monitoring station RS35U010500 with historical data available from 2007 to 2023 along with the hydrometric station 35003 at Ballygrania, is located 1300 m downstream of the monitoring stations for this study. The Owenmore River is larger than the Unshin and has moderate water quality status under the WFD. The national water quality monitoring station RS35O060600 with historical data available from 2007 to 2023, along with the hydrometric station 35001 Ballynacarrow, is located 200 m upstream of the study site.

### Sensor deployment

A YSI 6600 EDS V2–2 sonde (YSI Inc., Ohio, USA) was deployed at each of two river monitoring stations in the northwest of Ireland to continuously measure temperature (YSI6560), specific conductance (YSI 6560), and turbidity (nephelometer YSI 6136, including a mechanical wiper to minimize biofouling). Samples were taken every 15 min to monitor changes in these parameters over the study period. There was no permanent infrastructure associated with these temporary monitoring stations with power provided via internal batteries in the sondes. The sondes were initially deployed for a short, non-continuous test period from Nov 2022 to Jul 2023 for training the CANARY software (Table 1). The purpose of the initial deployment was to assess the suitability of the deployment methods and locations. The sondes were deployed in November 2022 as the highest levels of rainfall occur in December and January for most areas in Ireland (Nolan, 2015; see Supplementary References)). Analysis of climate long term averages show that annual average rainfall in Ireland has increased from 1961–1990 and 1991–2020, with the greatest increases of 6–12 % occurring in the west and north of Ireland (Coonan, Curley and Ryan, 2024; see Supplementary References).

**Table 1:** Training data sets for initial CANARY configuration at the monitoring stations for this study.

Configuration Variables and Parameters	River Unshin Dataset	River Owenmore Dataset
Matrix	Surface water (river)	Surface water (river)
Data Range (not continuous)	26/11/2022 to 07/07/2023	26/11/2022 to 21/07/2023
Monitoring location coordinates	54.175332, −8.462728	54.144790, −8.551777
Number of Sensor Signals tested	3	3
Monitoring Frequency (min)	15	15
No. of timesteps per dataset	15,860	17,040

**Fig. 2:**
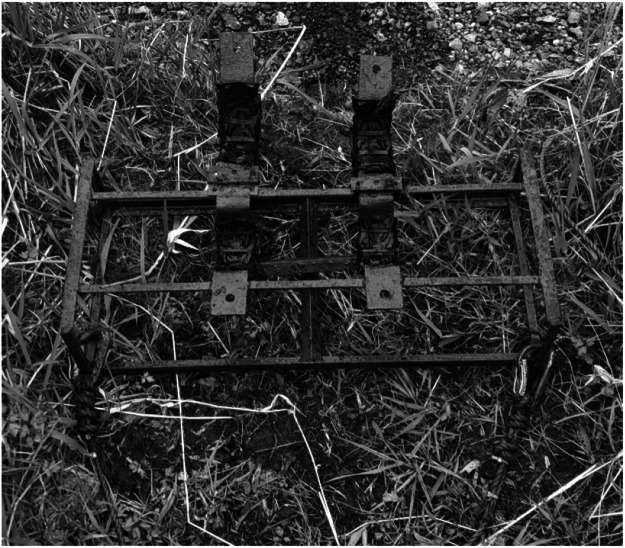
Purpose-built frame for sonde deployment on stream bed.

The method of deployment differed between the sites, with the sonde for the Owenmore River housed in a purpose-built steel frame, deployed mid width on the bed of the river and anchored in place with two large steel pins. The sonde was mounted in the frame and held within 2 circular steel cradles with hinged brackets on top which held the sensor in position. The hinged brackets were held closed using either cable ties or carabiners threaded through the holes drilled on the cradle ends (Fig. 2).

**Fig. 3:**
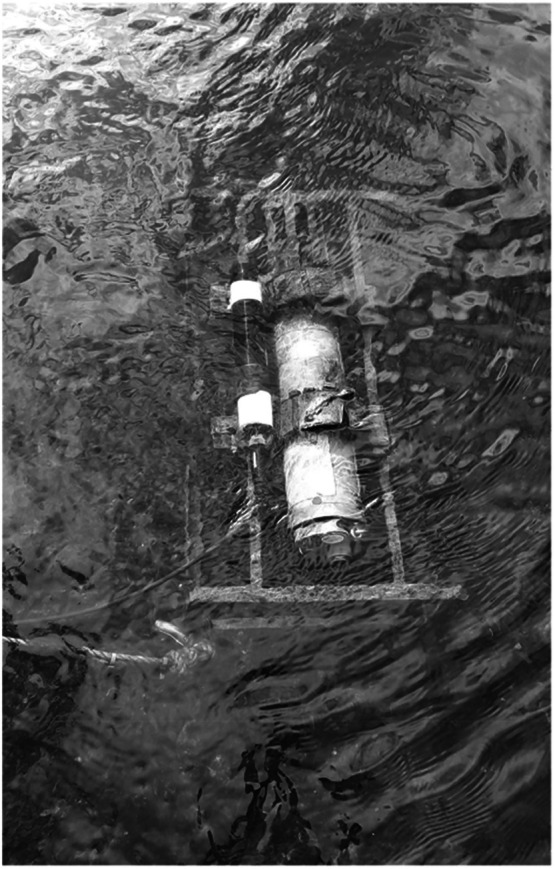
Sensor deployed on stream bed in river Owenmore.

The measuring face of the sensor was mounted in the frame opposite the direction of flow, and steel legs on the underside of the frame maintained the sensor position 10 cm above the riverbed (Fig. 3). A secondary backup mooring system consisting of two ropes was also attached at either end of the frame via stainless steel carabiners and ropes tied to large trees on the riverbank. The depth of the water at the deployment location varied between 60 cm and 150 cm and the deployment frame moved position on only one occasion, shifting 150 cm downstream during the very intense rainfall and river discharge associated with Storm Bert in Nov 2024.

**Fig. 4:**
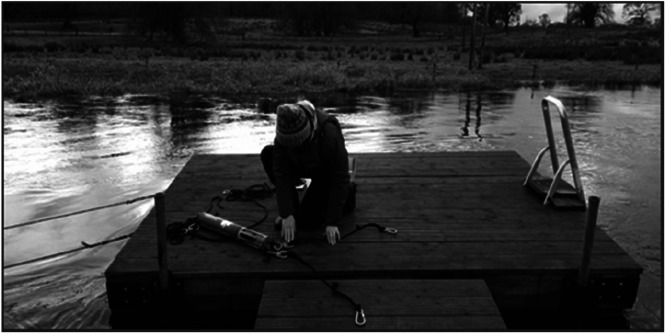
YSI Sonde on the river Unshin was mounted under a floating pontoon.

On the Unshin river the YSI 6600 EDS V2–2 sonde was deployed on the side of a privately owned recreational pontoon floating in the river (Fig. 4). The pontoon was mounted on steel poles which rose and fell with the river level, allowing the sensor to remain suspended in the water column at all times. The sonde was hung on the side of the pontoon using marine rope and stainless-steel carabiners, 30 cm below the water surface, and depending on the river flow, was approx. 120 cm to 270 cm above the riverbed. One of the advantages of this deployment arrangement was easy access to the sensor during high and low flow periods, with limited access occurring on only a few occasions over the study period, due to short term, localized flooding.

The data collected by each YSI sonde was stored on an internal sonde memory as a .txt file and downloaded by the operator to a laptop at regular intervals via EcoWatch Lite v1.0.5.15 (YSI Inc., Ohio, USA). The interval between measurements was set to 15 min. The .txt files were imported to R as per the workflow for water quality data processing for event detection as described in section 4. Prior to deployment, the sensors were allowed to run in water to check sensor operation and any ‘Start/Stop’ or ‘Out of the Water’ edits were made after the data was imported into R. This ensures transparency in data manipulation and the rationale for each manipulation is recorded in the code thereby improving reproducibility [17].

### Event detection

Hydrological event detection is a critical component of water resource management, flood forecasting, and environmental monitoring [18,19]. Land use along with catchment and climatic characteristics are strongly related to trends in water quality [20] with the analysis of time series data revealing underlying patterns, trends and anomalies [[21], [22], [23]].

#### Data management, data stewardship and reproducible research

**Fig. 5:**
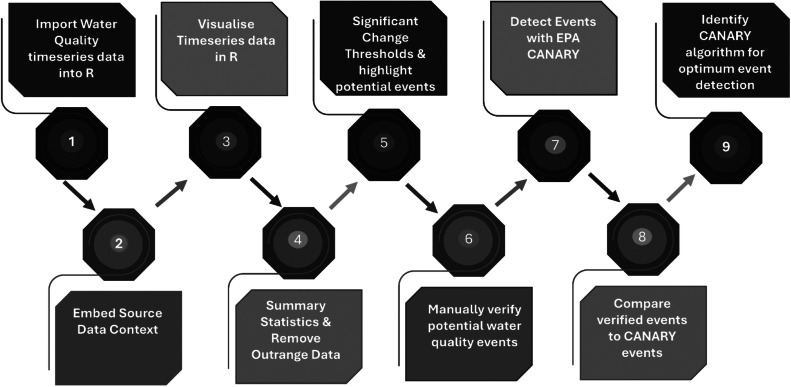
Protocol for data visualization and training of the CANARY event detection system at each water quality monitoring station.

The workflow presented for event detection is a nine-step process as outlined in Fig. 5. Each step in the workflow is comprehensively detailed in S1 User Guide in the supplementary data.

To minimize the issues associated with non-reproducible code, this workflow incorporated best practice in:

• Data management: processing of the data in R Studio v2024.04.2 + 764 (R Core Team, 2024; see Supplementary References)) was undertaken in accordance with the model of Import, Tidy, a loop of Transform, Visualize, and Model followed by Communicate (Wickham and Grolemund, 2017; see Supplementary References)).
• Data Stewardship: the source context of the data was embedded in the data imported to R using variable labels to ensure the integrity of the data persisted once imported (Pileggi, 2024; see Supplementary References)).
• Reproducible research: creating fully computationally reproducible research requires a workflow that manages the primary causes of non-reproducibility, and to this aim the method described in this research incorporated the four pillars of reproducibility; version control, dynamic document generation, dependency tracking and software management [24].

##### Data management

The data management model applied was a systematic approach to data analysis and informed Steps 1 to 4 of the workflow. Water quality data (Table 2) was imported, tidied, transformed and visualized prior to event detection. These steps included presenting a concise overview of the datasets in summary statistics tables, removing ‘out of the water’ data when the sonde was operating prior to deployment and removing ‘out-range’ data where values measured were outside the sensor range. The results of analysis on a data file alongside the relevant code were recorded using R markdown, and is presented as a detailed user guide available in Supplementary Materials S1 and on Github (Cronin and Taylor, 2025; see Supplementary References).

**Table 2:** Data imported for visualization and event detection.

River	Site Name	Turbidity (NTU) [YSI 6136] Frequency A	Temperature (^o^C) [YSI 6560] Frequency^A^	Specific conductance (µS/cm) [YSI 6560] Frequency^A^
Unshin	Upstream of Ballygrania	15 min	15 min	15 min
Owenmore (Sligo)	Ballynacarrow	15 min	15 min	15 min

A Source: Data from deployed YSI 6600 sondes.

##### Data stewardship

One of the challenges with increasing numbers of large datasets and the secondary use of data is data stewardship [25]. To preserve the integrity of the data in this research, Step 2 of the workflow included embedding the source data context via variable labels in R studio (Pileggi, 2024; see Supplementary References) detailing the location and sensor details for water quality monitoring.

##### Reproducible research

In water research, errors in computer code and the inability to reproduce analysis despite the code and the dataset being published, is common [26,13] with most errors being preventable if good coding practices are employed [14]. To minimize issues with non-reproducibility, the workflow in this research followed recommendations for research reproducibility using code [17,13,14] and a detailed user guide consisting of code alongside the output of analysis was developed. The four key components employed for reproducibility in R [24] in this workflow included:

• Git – for version control (git and RStudio were integrated via GitHub).
• renv – for managing R package dependencies.
• Docker – to manage the software environment.
• R markdown - for dynamic document generation.

An additional step of using an R project file, which places all project files in a single directory [27], and a standard folder management structure, consisting of ‘Data’, ‘Scripts’ and ‘Output_Files’ directories was also employed for each step in R studio.

#### Significant change thresholds using R and manually verifying potential water quality events

There are inherent challenges associated with water quality data including biofouling, calibration errors, spikes and sensor drift with anomalies or outliers associated with water quality events requiring validation to confirm true anomalies and minimise false positives [28]. Semi-automated validation approaches serve as a bridge between fully automated detection systems and manual expert analysis, aiming to improve the accuracy and reliability of event identification with methods often involving initial automated screening followed by manual verification ([16]; Herrera et al., 2022, see Supplementary References; [29]). Data collected from YSI sensors (YSI Inc., Ohio, USA) was processed in RStudio (Rstudio Team, 2024; see Supplementary References) as per Steps 1 to 6 of the workflow detailed in Fig. 5. This facilitated visualization of the turbidity, conductivity and temperature data, with the identification of significant change thresholds for turbidity used to aid the manual verification of potential water quality events. Significant change thresholds were identified using Tukey Fences test for outliers with outliers defined as greater than or equal to the upper bound of the calculated Tukey fence. The output of the significant change threshold (SCT) plot was exported as .csv files, and timesteps where the turbidity value exceeded the SCT for turbidity was assigned a value of 1 for true, or 0 for false.

Turbidity values obtained at the river monitoring stations examined did not show a diurnal trend, and baseline levels for turbidity at both sites were low. Turbidity has been used to monitor fine sediment transport in rivers due to storms [30], as a proxy for suspended solids [31], and to detect metal transport in rivers [32]. Patterns of significant peaks in turbidity were observed in the datasets. The analyst then manually verified values that exceeded the SCT for turbidity by visually scanning the turbidity plots and editing the .csv file. Data either side of the values exceeding the SCT may also be identified as potential events by the analyst.

#### Analysis by US EPA CANARY event detection software

Event identification was carried out as per Step 7 in the workflow by processing ‘training data’ (Table 1) through the CANARY event detection system which uses algorithms to identify user defined ‘outlier’ datapoints in time series water quality data, whilst striving to minimize the detection of ‘false alarms’ (Hart and McKenna, 2012). The linear prediction–correction filter (LPCF) algorithm was used in CANARY to produce an alarm when at least one of the sensor signals deviated from the baseline for a required time period [16,33,34]. When enough user defined outliers occur, an alarm is triggered, and CANARY identifies the water quality profile as a probable event. Problems with turbidity measurements including random spikes have been well documented [35]. However, this feature of CANARY which can filter out random spikes overcomes one of the most significant challenges with continuous monitoring, especially for turbidity, where short lived signal spikes, caused by air for example, may lead to false positives and are not indicative of a water quality event. The data files processed in CANARY and the output files for the CANARY event detection are available in the ’07 EPA CANARY Event Detection’ folder (Cronin and Taylor, 2025; see Supplementary References), and details for the CANARY output files are provided in supplementary material S3.

#### Matching between manually verified events and CANARY identified events

For Step 8 of the workflow, the approach used in previous research was used to match events and identify the most suitable CANARY algorithm for the data [16]. Matching events was carried out by manually verifying changes in the turbidity trend of the data. R code was used to automatically compare CANARY alarms to the manually identified events for each of the 96 algorithms used to train CANARY. Matches were classified as either a full match or a partial match. An Exact match is when the start and end timestamp for an event in CANARY matches the start and end timestamp of the manually identified event file. A Partial event match is where the start or end time of an event in CANARY overlaps with a manually identified event or vice versa. The logic for identifying full and partial matches between manually verified events and EPA CANARY identified events (Burkhardt, J. 2024, see Supplementary References) is provided in the supplementary material (S2 & S3) together with an example of the match logic applied to a dataset (S4). The limitations of this manually verified approach are that the analyst requires familiarity with screening time series datasets, it is labor intensive, can be difficult to replicate, and analysts may have difficulty in detecting subtle patterns and changes. The authors plan to investigate how well the significant change thresholds (described in Step 5 of the workflow) compares to manually identifying potential outliers to semi-automate this process in the future.

#### Identify CANARY algorithm for optimum event detection

The optimum CANARY algorithm for event detection, Step 9 of the workflow, was identified by comparing the number of potential water quality events identified by CANARY to the number of manually verified water quality events [16]. The Dat_Files directory contains each of the CANARY algorithm output files matched to the manually verified events file. An example of how matching can be verified by the user is included in the file S4 'Example of match logic applied to _Dat_For_OW061024_051124C_LPCF_BED6_ET0.89063alg_1′ in the supplementary material folder. The output file ‘algo scores’ summarizes the number of full and partial matches between CANARY alarms and manually identified events for each CANARY algorithm, and assigns a percent agreement to the total number of verified events. This can be used to identify the most suitable algorithm for the particular monitoring station based on the data provided to 'train' CANARY. The algorithm with the highest percentage of matches is chosen as the algorithm with the most suitable parameters for detecting potential water quality events at that monitoring station. Adequate data points covering a range of events over a time period, is required to train CANARY and identify the most suitable algorithm. The optimum time period required depends on the frequency of events and the variability in event profiles at each monitoring station.

### Sensor calibration

One of the objectives of continuous, high frequency monitoring is to collect the most accurate and complete datasets for monitoring purposes (Hoppe, 2023; see Supplementary References). Service and calibration of the sensors was carried out off site in the laboratory as per manufacturer’s instructions. Data was downloaded to a laptop using EcoWatch Lite software and sensors were cleaned, inspected for damage, water intrusion, battery life and adequate data storage memory. Calibration for turbidity was via a two-point calibration with deionized water and 126 NTU standard (part no. 607300), and for conductivity using 1413µS/cm standard conductivity calibration solution as per manufacturer’s instructions. Prior to deployment the sensors were allowed to run in stream water to check that the sensor was logging, and turbidity, conductivity and temperature values were consistent. Site visits were carried out every 2–4 weeks for sonde maintenance. Cleaning of sensors was carried out using a soft-bristle brush to remove debris from the outside of the sensors, wiping with a damp towel and then rinsing with deionized water. The frequency of sensor maintenance and appropriate service intervals is determined by the rate of sensor fouling, and this rate depends on the monitoring location, the sensor type, hydrological conditions and the time of year (Wagner et al., 2006; see Supplementary References). Fouling was not found to be a particular issue at either site in the autumn, winter and spring but during the summer months there was significant accumulation of filamentous algae on the frame and anchor ropes at the Owenmore site.

### Validation

#### Data validation

Instrument checks to monitor sensor drift and to verify the operation of the sondes were carried out on site. River turbidity, specific conductance and temperature were measured using calibrated field meters (Wagner et al., 2006; see Supplementary References). A portable turbidity meter (HACH 2100Q Portable Turbidimeter), and a portable conductivity/temperature meter (HACH HQ series Conductivity) with a calibration criterion of ± 10 % of the measured value for specific conductance and temperature and ± 20 % or within 1.0 NTU for turbidity. It is the relative change in turbidity, specific conductance and temperature and not the absolute values that were of interest in this study as it is the relative change in data values of a parameter from the baseline that is used by US EPA CANARY for event identification. Significant drift was not detected in the datasets and the wiper system on the turbidity sensors (YSI 6136) was found to be effective at maintaining a clean window. Differences in measured turbidity between different sensors has previously been identified as an issue for turbidity monitoring [36] so the same turbidity sensors were used throughout this study.

#### R code validation

The code written by the corresponding author (who is not formally trained in coding) was validated to identify code errors, to optimise code for efficiency and scalability, to maintain consistency in code across the workflow and to identify any potential issues. The code was initially written to process a single dataset at a time. Due to the large number of CANARY files to be matched, the code was amended to automate this processing which also involved standardising filename conventions and standardising file paths to directories. The code was edited to provide a more efficient approach to joining data frames and was also reviewed and edited for the use of R packages for functions that can be easily replicated using base R.

#### Method validation

Results for the river Unshin and Owenmore data processed through the workflow are comparable with the results of previous studies [16,34,37]. CANARY generated alarms for all 47 of the manually identified events for the river Owenmore turbidity data i.e. 100 % agreement and 38 of the 39 manually identified events i.e. 97 % agreement for the river Unshin turbidity data, compared to 94 % agreement in another surface water study [16]. There were no exact matches (i.e. identical start time and end time) between the CANARY events and the manually identified events, so all matches were partial matches. Different algorithm configurations for each of the Owenmore and Unshin monitoring stations were identified for optimum event identification at those sites. This is an advantage of the US EPA CANARY event detection software as locations in a river system often respond differently to storm events [38]. The authors recognise the importance of validating these results using larger datasets and plan to undertake further research using expanded datasets and monitoring locations.

## Conclusion

The aim of this research was to develop a comprehensive, reproducible, practical, open-source workflow for high frequency water quality monitoring suitable for multiple users. This method describes all steps in the process detailing the use of R, R markdown and US EPA CANARY software for data import, data cleaning, data visualization, data integrity, along with site-specific CANARY event system configuration for the detection of potential water quality events. Application of this method to two, temporary, remote monitoring stations in a single catchment demonstrated the ability of US EPA CANARY to successfully identify 100 % (n 47) and 97 % (n 39) of the manually identified turbidity events.

High frequency water quality monitoring can provide timely data for real-time strategic adaptation, for the identification of significant water quality pressures and for the identification of appropriate water quality improvement measures in surface waters, but comprehensive, practical protocols such as the method presented here are required to accelerate the adoption of high frequency water quality monitoring and realize its full potential.

## Limitations

The method described is for hydrodynamic water quality event monitoring in small rivers and alternative deployment methods may be required in larger rivers or other waterbodies. Access to sensors for calibration, maintenance and recovery back to the laboratory following hydrodynamic events can be challenging due to flooding, high water levels and the lifting of pontoons from anchor points as occurred in this research. The processing of data for event detection as described in this method is the same regardless of the deployment method. The frequency of sensor maintenance and appropriate service interval is site and sensor specific and must be determined for each deployment. Characterisation of event length at individual river stations is required to optimise the event timeout parameter in configuring CANARY and the amount of data required to ‘train’ the CANARY system will depend on the profile of water quality events at each monitoring station.

## Ethics statements

Not applicable.

## CRediT authorship contribution statement

**L. Cronin:** Conceptualization, Methodology, Software, Validation, Data curation, Writing – original draft, Formal analysis, Software, Visualization, Investigation, Writing – review & editing. **C.M. Taylor:** Conceptualization, Methodology, Software, Validation, Data curation, Writing – original draft, Formal analysis, Software, Visualization, Investigation, Writing – review & editing. **C. Briciu Burghina:** Validation, Data curation, Writing – original draft, Writing – review & editing. **F.E. Lucy:** Supervision, Software, Validation, Writing – review & editing. **F. Regan:** Conceptualization, Methodology, Software, Supervision, Software, Validation, Writing – review & editing.

## Declaration of competing interest

The authors declare that they have no known competing financial interests or personal relationships that could have appeared to influence the work reported in this paper.

## Data Availability

We have shared a link to a repository for the code/data referenced in the article

## Appendix Supplementary materials

Supplementary material *and/or* additional information The supplementary material for this research includes a detailed step by step user guide to the workflow for importing the data, running the R code, and for the output of the analysis provided as Supplementary Files S1-S4. S1: User Guide - Timeseries Water Quality data processing with R and CANARY for Hydrodynamic Event Monitoring. S2: Details for CANARY Output Files S3: Logic for identifying full and partial matches between manually verified events and CANARY events. S4: Example of match logic applied to _Dat_For_OW061024_051124C_LPCF_BED6_ET0.89063alg_1. S5: Supplementary References for non-peer reviewed publications. The source code used for visualisation and data analysis is publicly available on GitHub (Cronin and Taylor, 2025) along with a README file describing how to reproduce the results. The repository contains data, an interactive R markdown file with code and results of analysis, so users can apply the workflow directly to their own datasets. The repository is structured as per the nine-step protocol for data visualization and training of the CANARY event detection system detailed in Fig. 5 and detailed in the user guide. All materials are freely accessible under the MIT license. Image, application 1
