# Supplementary material for: An open-source workflow for identifying hydrodynamic water quality events in rivers by continuous water quality monitoring and time-series data processing using R and US EPA CANARY
Source: MethodsX. 2025 Jul 27;15:103538. doi: 10.1016/j.mex.2025.103538 (PMC12347471; doi:10.1016/j.mex.2025.103538)
Supplement: Supplementary file 1 — Supplementary material and/or additional information The supplementary material for this research includes a detailed step by step user guide to the workflow for importing the data, running the R code, and for the output of the analysis provided as Supplementary Files S1-S4. S1: User Guide - Timeseries Water Quality data processing with R and CANARY for Hydrodynamic Event Monitoring. S2: Details for CANARY Output Files S3: Logic for identifying full and partial matches between manually verified events and CANARY events. S4: Example of match logic applied to _Dat_For_OW061024_051124C_LPCF_BED6_ET0.89063alg_1. S5: Supplementary References for non-peer reviewed publications. The source code used for visualisation and data analysis is publicly available on GitHub (Cronin and Taylor, 2025) along with a README file describing how to reproduce the results. The repository contains data, an interactive R markdown file with code and results of analysis, so users can apply the workflow directly to their own datasets. The repository is structured as per the nine-step protocol for data visualization and training of the CANARY event detection system detailed in Fig. 5 and detailed in the user guide. All materials are freely accessible under the MIT license. [file S1 User Guide R and CANARY for Hydrodynamic Event Monitoring.pdf › S1 User Guide R and CANARY for Hydrodynamic Event Monitoring.pdf]

Timeseries Water Quality data processing with R and CANARY for Hydrodynamic Event Monitoring

User Guide

A full functional water quality project written in R showing a simple step-by-step workflow for processing time series data from a water quality sensor and how to use that data for the identification of potential water quality events.

Authors: Lisa Cronin, Cian Taylor
2025-04-25

For the supporting code and data: https://github.com/LCroninATU/Workflow-for-identifying-water-quality-hydrodynamic-events-in-time-series-data

Please contact me with questions by email Lisa.cronin@atu.ie. For more information on projects being carried out at DCU Water Institute https://dcuwater.ie/ led by Prof. Fiona Regan

Institiúid Uisce DCU
DCU Water Institute

Introduction

This guide is based on and designed to be used in conjunction with the R markdown file available on github at https://github.com/LCroninATU/Workflow-for-identifying-water-quality-hydrodynamic-events-in-time-series-data . The markdown file contains a detailed description of the workflow, the R code and output files from that R code, and from the EPA CANARY event detection software.

Cite the repository

If you use this software in your work, please cite it using the following metadata.
Lisa Cronin and Cian Taylor (2025) “LCroninATU/Workflow-for-identifying-water-quality-hydrodynamic-events-in-time-series-data: V1.0.0”.
DOI: https://doi.org/10.5281/zenodo.15358190

Description

This project contains a concise, reproducible, open-source workflow detailing the use of R, R markdown and EPA CANARY event detection software and was designed for catchment scientists, researchers and those working in on stream hydrochemistry, diffuse pollution or any EU Water Framework Directive water quality projects who require a simple, detailed workflow that can be easily replicated requiring minimum expertise in R. One of the primary objectives of this project was to provide simple code that non-coders can follow and to produce outputs at each step of the workflow so the user can validate the code for their data. This project includes a sample data file and a detailed markdown so you can take the sample file provided through each step of the workflow, prior to applying the code to your own data.

Each part of this project is simple sample code which details how to do the following:

- import time series data,
- clean the data,
- visualize the data,
- maintain data integrity,
- manually identify potential water quality events,
- match those events to the output of EPA CANARY event detection software,
- identify the most suitable configuration of EPA CANARY for that particular water quality monitoring station.

Open-source Workflow for Hydrodynamic Water Quality Event Monitoring in small rivers using R & EPA CANARY

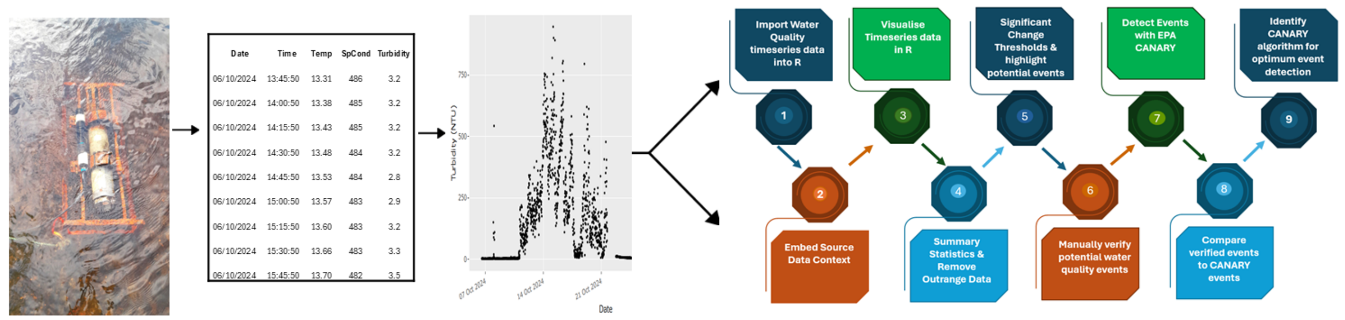

Sensor Deployment | Data Collection | Event Detection | Workflow for Water Quality Data Processing for Event Detection

Getting Started

Dependencies

Needs recent install of R and Rstudio.

Dependencies are managed through the renv package which was created by RStudio developers specifically for this purpose. The "renv" package will create a package library which is specific to this project. Thus you can ensure that you have the correct version of all required packages (those used in the original build). Outside of the project, you can use or install whatever package versions you prefer, those inside the project space will not be affected.

Installing

Install R and Rstudio latest versions.

Install the "renv" package by typing install.packages("renv") in the R console.

You need to have a github account and a local install of git on your machine.

Open a git BASH (start the "git BASH app") and introduce yourself to git. This will allow Rstudio to connect successfully to github.

Open RStudio and create a New Rstudio Project.

Select the option to install from "Version Control".

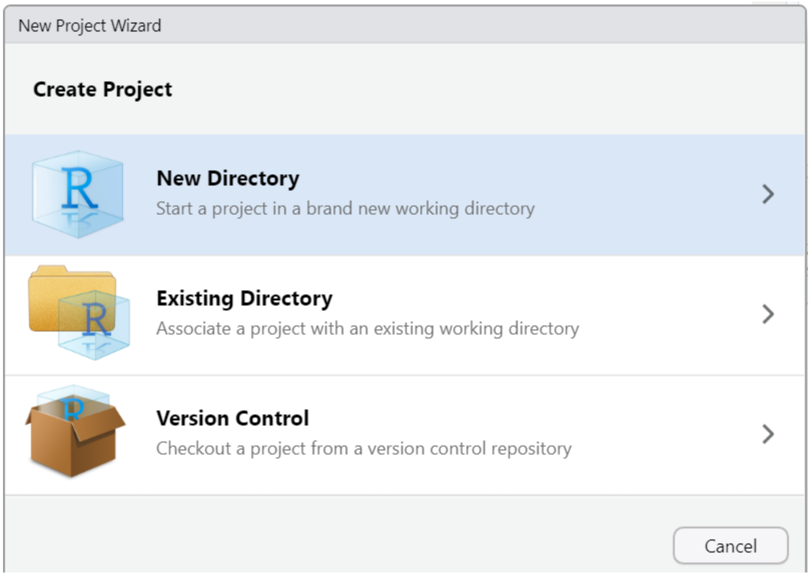

Select "git".
In the dialog box that follows, fill the first two boxes in as shown below:
Repository URL: https://github.com/LCroninATU/Workflow-for-identifying-water-quality-hydrodynamic-events-in-time-series-data

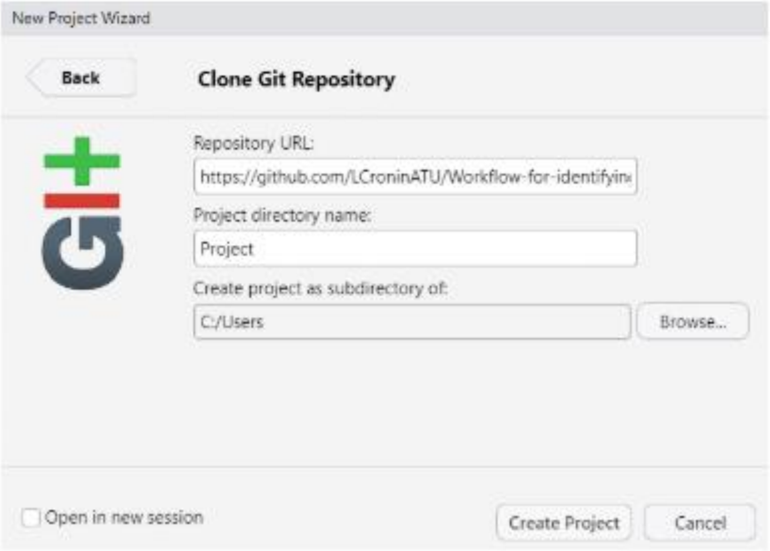

In the third box, browse to the folder on your local machine where you want to install the project.
Select "Create Project"
Ensure that "renv" is enabled for the project as follows:
In Rstudio click on "Tools" menu, followed by "Project Options".
Select "Environments" from the list of icons on the left of the dialog box.
Ensure the the box beside "Use renv with this project" is ticked as shown:

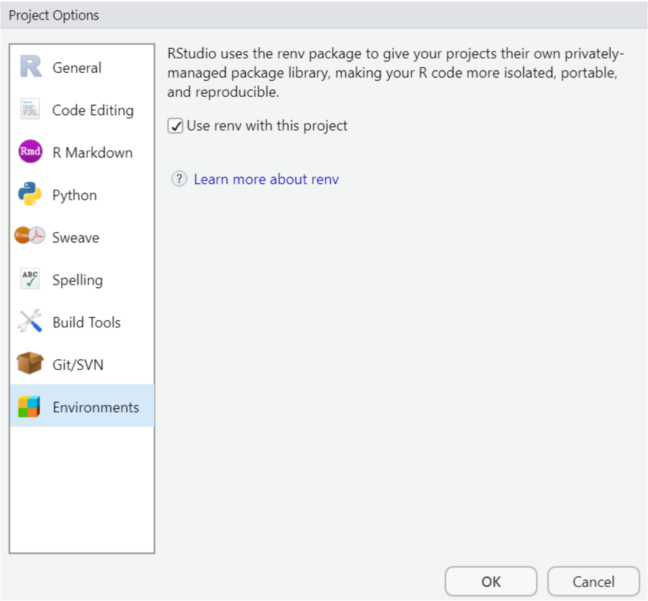

Now check that the correct versions of all required packages are installed by running the command renv::status(), from the R console. You will see a list of any dependency issues.
To resolve any dependency issues, type the command renv::restore(). The correct version of every required dependent package will now install.
At this point you should be able to execute any of the scripts or RMd documents within any directory.

Executing program

Follow the step by step instructions, the code and the output of the code in the 'Workflow for Hydrodynamic Event Detection' markdown file.
The scripts are also available but the markdown file is the place to start for users who are new to R.

Help

More information on reusing other people's code in your project
https://docs.github.com/en/get-started/learning-to-code/reusing-other-peoples-code-in-your-projects

How to tweak this project for your own use

We would encourage you to clone this project from Github and rename this project to use for your own data.

Contents

R Studio Projects

1.0 Import Water Quality Data

1.1 Load the packages

1.2 The working Directory

1.2 Set the timezone (no daylight saving)

1.3 Import the data file

1.4 Tidy the data and format the imported water quality data

1.5 Combine the files into a single dataframe

2.0 Data Integrity

3.0 Visualisation of Water Quality Data

4.0 Summary Statistics

5.0 Define significant change thresholds for turbidity and highlight on plots to identify events

6.0 Manually Identify Potential Water Quality Events with the aid of the interactive turbidity plot.

7.0 Event Detection using EPA CANARY

8.0 Compare manually identified Owenmore turbidity events to events identified by CANARY

9.0 Compare the Owenmore CANARY files to the manually identified events to identify the most suitable CANARY algorithm.

References

R Studio Projects

R Studio uses projects to store all the data, R scripts, output files including figures, tables etc associated with each individual project in one place.
There is a project file associated with this project called 'Project Workflow for detecting Hydrodynamic Water Quality Events'. You must double click on this file to open this project *prior* to running the code. Otherwise the code may not run correctly.

1.0 Import Water Quality Data

One of the first steps in processing data is importing the data into R.

1.1 Load the packages

You may need to install some of the packages before loading them. Go to the output pane on the bottom right of the screen, go to packages, install, enter the name of the package under packages and click install. You may be asked to enter 'Y' in the console to confirm the installation.

### for working with tabular data library(data.table) *### for data wrangling* library(dplyr) *### for working with dates & times* library(lubridate) *### for labelling of variables* library(labelled) *### breaks and labels for axes and legends on graphs* library(scales) *### for data visualisation* library(ggplot2) *### tidy ways to summarise, visualise, and manipulate missing data* library(naniar) *### for creating interactive web-based graphs* library(plotly) *### for combining multiple plots into a single figure* library(cowplot) *### for arranging graphical elements* library(grid) *### to arrange multiple grid-based plots* library(gridExtra) *### for publication quality tables* library(stargazer) *### to make working with strings as easy as possible* library(stringr) *### tool to tidu up messy datasets* library(tidyr) *### to import, organize, manipulate, and visualise data* library(tidyverse)

1.2 The working Directory

This is the location on your pc which r will use for reading and writing files. R operates a working directory where it looks for files that you want to load and where it saves files you request R to save. This working directory is displayed at the top of the console window. The 'here' package sets the top level of the project folder. It's very important to open the project file as described previously **before** trying to run any code to avoid problems with files and the working directory.

library(here) *# for file referencing*

1.2 Set the timezone (no daylight saving)

Sys.setenv(TZ='UTC')
Sys.timezone()
[1] "UTC"

1.3 Import the data file

There are two options in this workflow to import the data files, either as individual files or loading multiple files together. Two csv files have been provided as an example, OW061024

and OW221024. OW refers to the name of the river where the sonde was deployed, in this case the Owenmore River, Sligo, and 061024 is the date the sonde was deployed in the river.

Option 1: Import a single .csv data file and specify the variables to be loaded

In this case 'Date', 'Time', 'Temp', 'SpCond' and 'Turbid+'. Watch out for spaces in column names in some .txt files when specifying the variable name, it must match exactly. **Note: For these files, only data for temperature, specific conductivity and turbidity are valid as only these sensors were calibrated.**

```
OW061024<-fread("01_ImportYSI_Data/Data/OW061024.txt",skip=1, header=T,select = c("
OW221024<-fread("01_ImportYSI_Data/Data/OW221024.txt",skip=1, header=T,select = c("
```

Display the structure and properties of the imported file.

```
# Display the structure of the imported file str(OW061024)
## Classes 'data.table' and 'data.frame':   1466 obs. of  5 variables:
##  $      Date: chr  "06/10/2024" "06/10/2024" "06/10/2024" "06/10/2024" ...
##  $    Time  : chr  "12:30:50" "12:45:50" "13:00:50" "13:15:50" ...
##  $  Temp    : chr  "13.04" "13.09" "14.50" "15.76" ...
##  $ SpCond   : chr  "265" "264" "6" "5" ...
##  $ Turbid+  : chr  "1.2" "1.6" "0.7" "0.8" ... ##  - attr(*, ".internal.selfref")=<externalptr>
str(OW221024)
## Classes 'data.table' and 'data.frame':   1354 obs. of  5 variables:
##  $      Date: chr  "22/10/2024" "22/10/2024" "22/10/2024" "22/10/2024" ...
##  $    Time  : chr  "10:30:50" "10:45:50" "11:00:50" "11:15:50" ...
##  $  Temp    : chr  "12.59" "12.47" "12.37" "12.27" ...
##  $ SpCond   : chr  "278" "265" "266" "266" ...
##  $ Turbid+  : chr  "0.2" "0.4" "0.3" "0.3" ...
##  - attr(*, ".internal.selfref")=<externalptr>
# Display the class or the properties of the imported file, this affects how the im
class(OW061024)
## [1] "data.table" "data.frame"
class(OW221024)
## [1] "data.table" "data.frame"
```

Option 2: Import Multiple Files into R & bind them together.

Alternative option for importing files is to import the entire file and subset for the variables of interest. **Note: For these files, only data for temperature, specific conductivity and turbidity are valid as only these sensors were calibrated.**

```
# Import the data file
OW061024<-fread("01_ImportYSI_Data/Data/OW061024.txt",skip=1, header=T,)[-1:-2]
OW221024<-fread("01_ImportYSI_Data/Data/OW221024.txt",skip=1, header=T)[-1:-2]
str(OW061024)
## Classes 'data.table' and 'data.frame':   1466 obs. of  15 variables:
##  $      Date: chr  "06/10/2024" "06/10/2024" "06/10/2024" "06/10/2024" ...
```

```
## $    Time  : chr  "12:30:50" "12:45:50" "13:00:50" "13:15:50" ...
## $ Temp     : chr  "13.04" "13.09" "14.50" "15.76" ...
## $ SpCond    : chr  "265" "264" "6" "5" ...
## $ Cond     : chr  "0.204" "0.204" "0.005" "0.004" ...
## $  TDS     : chr  "0.172" "0.172" "0.004" "0.003" ...
## $   Sal    : chr  "0.13" "0.13" "0.00" "0.00" ...
## $  Press   : chr  "14.287" "14.286" "14.282" "14.281" ...
## $  Depth   : chr  "-0.265" "-0.266" "-0.268" "-0.270" ...
## $   pH     : chr  "5.17" "5.08" "5.16" "4.53" ...
## $     pH   : chr  "59.3" "62.5" "59.8" "81.7" ...
## $  Chl     : chr  "0.9" "1.2" "0.2" "0.1" ...
## $  Chl     : chr  "0.2" "0.3" "0.0" "0.0" ...
## $ Turbid+   : chr  "1.2" "1.6" "0.7" "0.8" ...
## $ Battery   : chr  "11.7" "11.7" "11.7" "11.7" ... ##  - attr(*, ".internal.selfref")=<externalptr>
str(OW221024)
## Classes 'data.table' and 'data.frame':   1354 obs. of  15 variables:
## $      Date: chr  "22/10/2024" "22/10/2024" "22/10/2024" "22/10/2024" ...
## $    Time  : chr  "10:30:50" "10:45:50" "11:00:50" "11:15:50" ...
## $ Temp     : chr  "12.59" "12.47" "12.37" "12.27" ...
## $ SpCond    : chr  "278" "265" "266" "266" ...
## $ Cond     : chr  "0.212" "0.202" "0.202" "0.202" ...
## $  TDS     : chr  "0.181" "0.173" "0.173" "0.173" ...
## $   Sal    : chr  "0.13" "0.13" "0.13" "0.13" ...
## $  Press   : chr  "14.750" "14.756" "14.759" "14.761" ...
## $  Depth   : chr  "0.062" "0.066" "0.068" "0.069" ...
## $   pH     : chr  "6.23" "6.34" "6.42" "6.42" ...
## $     pH   : chr  "23.1" "19.5" "16.7" "16.8" ...
## $  Chl     : chr  "0.8" "0.2" "0.4" "0.6" ...
## $  Chl     : chr  "0.2" "0.1" "0.1" "0.1" ...
## $ Turbid+   : chr  "0.2" "0.4" "0.3" "0.3" ...
## $ Battery   : chr  "11.3" "11.3" "11.3" "11.4" ... ##  - attr(*, ".internal.selfref")=<externalptr>
class(OW061024) ## [1] "data.table" "data.frame"
class(OW221024)
## [1] "data.table" "data.frame"
```

Display the column names, this provides the exact column name (including any spaces!) when choosing columns of interest and then drop any unwanted variables by entering NULL against these.

```
# Display the column names colnames(OW061024)
##  [1] "      Date" "    Time"   " Temp"      "SpCond"     " Cond"
##  [6] "  TDS"      "   Sal"     "  Press"    "  Depth"    "   pH"     ## [11] "     pH"     "  Chl"      "  Chl"
"Turbid+"    "Battery"
colnames(OW221024)
```

```
## [1] "      Date" "    Time"   " Temp"      "SpCond"     " Cond"
## [6] "  TDS"       "   Sal"      "  Press"     "  Depth"     "   pH"
## [11] "     pH"    "  Chl"      "  Chl"      "Turbid+"    "Battery"
# Subset for the variables of interest in OW061024 by dropping each column not requ
OW061024$` Cond`<-NULL
OW061024$`  TDS`<-NULL
OW061024$`   Sal`<-NULL
OW061024$`  Press`<-NULL
OW061024$`  Depth`<-NULL
OW061024$`   pH`<-NULL
OW061024$`     pH`<-NULL
OW061024$`  Chl`<-NULL
OW061024$"  Chl"<-NULL
OW061024$Battery<-NULL
# Subset for the variables of interest in OW221024 by dropping each column not requ
OW221024$` Cond`<-NULL
OW221024$`  TDS`<-NULL
OW221024$`   Sal`<-NULL
OW221024$`  Press`<-NULL
OW221024$`  Depth`<-NULL
OW221024$`   pH`<-NULL
OW221024$`     pH`<-NULL
OW221024$`  Chl`<-NULL
OW221024$"  Chl"<-NULL
OW221024$Battery<-NULL
# View the dataframe to see what variables have been retained (Note there is a numb
str(OW061024)
```

```
## Classes 'data.table' and 'data.frame':   1466 obs. of  5 variables:
##  $       Date: chr  "06/10/2024" "06/10/2024" "06/10/2024" "06/10/2024" ...
##  $     Time  : chr  "12:30:50" "12:45:50" "13:00:50" "13:15:50" ...
##  $  Temp     : chr  "13.04" "13.09" "14.50" "15.76" ...
##  $ SpCond    : chr  "265" "264" "6" "5" ...
##  $ Turbid+   : chr  "1.2" "1.6" "0.7" "0.8" ... ##  - attr(*, ".internal.selfref")=<externalptr>
str(OW221024)
## Classes 'data.table' and 'data.frame':   1354 obs. of  5 variables:
##  $       Date: chr  "22/10/2024" "22/10/2024" "22/10/2024" "22/10/2024" ...
##  $     Time  : chr  "10:30:50" "10:45:50" "11:00:50" "11:15:50" ...
##  $  Temp     : chr  "12.59" "12.47" "12.37" "12.27" ...
##  $ SpCond    : chr  "278" "265" "266" "266" ...
##  $ Turbid+   : chr  "0.2" "0.4" "0.3" "0.3" ...
##  - attr(*, ".internal.selfref")=<externalptr>
```

1.4 Tidy the data and format the imported water quality data

```
#Combine date and time columns to form DateTime & change from chr to as.POSIXct (as
OW061024$DateTime<-with(OW061024, dmy(OW061024$`      Date`) + hms(OW061024$`    Ti
OW221024$DateTime<-with(OW221024, dmy(OW221024$`      Date`) + hms(OW221024$`    Ti
str(OW061024)
```

```
## Classes 'data.table' and 'data.frame':   1466 obs. of  6 variables:
##  $       Date: chr  "06/10/2024" "06/10/2024" "06/10/2024" "06/10/2024" ...
##  $     Time  : chr  "12:30:50" "12:45:50" "13:00:50" "13:15:50" ...
##  $  Temp     : chr  "13.04" "13.09" "14.50" "15.76" ...
##  $ SpCond    : chr  "265" "264" "6" "5" ...
##  $ Turbid+   : chr  "1.2" "1.6" "0.7" "0.8" ...
##  $ DateTime  : POSIXct, format: "2024-10-06 12:30:50" "2024-10-06 12:45:50" ... ##  - attr(*, ".internal.selfref")=<externalptr>
```

```
str(OW221024)
```

```
## Classes 'data.table' and 'data.frame':   1354 obs. of  6 variables:
##  $       Date: chr  "22/10/2024" "22/10/2024" "22/10/2024" "22/10/2024" ...
##  $     Time  : chr  "10:30:50" "10:45:50" "11:00:50" "11:15:50" ...
##  $  Temp     : chr  "12.59" "12.47" "12.37" "12.27" ...
##  $ SpCond    : chr  "278" "265" "266" "266" ...
##  $ Turbid+   : chr  "0.2" "0.4" "0.3" "0.3" ...
##  $ DateTime  : POSIXct, format: "2024-10-22 10:30:50" "2024-10-22 10:45:50" ... ##  - attr(*, ".internal.selfref")=<externalptr>
```

```
# Rename the column names
OW061024<-setnames(OW061024, "Turbid+", "Turbidity")
OW061024<-setnames(OW061024," Temp", "Temp")       # this removes the space in fro
OW221024<-setnames(OW221024, "Turbid+", "Turbidity") OW221024<-setnames(OW221024," Temp", "Temp") colnames(OW061024)
```

```
## [1] "      Date" "    Time"   "Temp"      "SpCond"     "Turbidity" ## [6] "DateTime"
colnames(OW221024)
## [1] "      Date" "    Time"   "Temp"      "SpCond"     "Turbidity"
## [6] "DateTime"
# Change variable format from chr to numeric str(OW061024)
## Classes 'data.table' and 'data.frame':   1466 obs. of  6 variables:
##  $       Date: chr  "06/10/2024" "06/10/2024" "06/10/2024" "06/10/2024" ...
##  $     Time  : chr  "12:30:50" "12:45:50" "13:00:50" "13:15:50" ...
##  $ Temp      : chr  "13.04" "13.09" "14.50" "15.76" ...
##  $ SpCond    : chr  "265" "264" "6" "5" ...
##  $ Turbidity : chr  "1.2" "1.6" "0.7" "0.8" ...
##  $ DateTime  : POSIXct, format: "2024-10-06 12:30:50" "2024-10-06 12:45:50" ... ##  - attr(*, ".internal.selfref")=<externalptr>
```

```
str(OW221024)
## Classes 'data.table' and 'data.frame':   1354 obs. of  6 variables:
##  $      Date: chr  "22/10/2024" "22/10/2024" "22/10/2024" "22/10/2024" ...
##  $     Time  : chr  "10:30:50" "10:45:50" "11:00:50" "11:15:50" ...
##  $ Temp      : chr  "12.59" "12.47" "12.37" "12.27" ...
##  $ SpCond    : chr  "278" "265" "266" "266" ...
##  $ Turbidity : chr  "0.2" "0.4" "0.3" "0.3" ...
##  $ DateTime  : POSIXct, format: "2024-10-22 10:30:50" "2024-10-22 10:45:50" ...
##  - attr(*, ".internal.selfref")=<externalptr>
```

```
OW061024$SpCond<-as.numeric((OW061024$SpCond))
OW061024$`Temp`<-as.numeric((OW061024$Temp))
OW061024$Turbidity<-as.numeric((OW061024$Turbidity))
OW221024$SpCond<-as.numeric((OW221024$SpCond))
OW221024$`Temp`<-as.numeric((OW221024$Temp))
OW221024$Turbidity<-as.numeric((OW221024$Turbidity))
# Change variable format from chr to numeric # View the structure after the format changes
str(OW061024)
## Classes 'data.table' and 'data.frame':   1466 obs. of  6 variables:
##  $      Date: chr  "06/10/2024" "06/10/2024" "06/10/2024" "06/10/2024" ...
##  $     Time  : chr  "12:30:50" "12:45:50" "13:00:50" "13:15:50" ...
##  $ Temp      : num  13 13.1 14.5 15.8 16.4 ...
##  $ SpCond    : num  265 264 6 5 6 486 485 485 484 484 ...
##  $ Turbidity : num  1.2 1.6 0.7 0.8 1.8 3.2 3.2 3.2 3.2 2.8 ...
##  $ DateTime  : POSIXct, format: "2024-10-06 12:30:50" "2024-10-06 12:45:50" ... ##  - attr(*, ".internal.selfref")=<externalptr>
```

```
str(OW221024)
## Classes 'data.table' and 'data.frame':   1354 obs. of  6 variables:
##  $      Date: chr  "22/10/2024" "22/10/2024" "22/10/2024" "22/10/2024" ...
##  $     Time  : chr  "10:30:50" "10:45:50" "11:00:50" "11:15:50" ...
##  $ Temp      : num  12.6 12.5 12.4 12.3 12.1 ...
##  $ SpCond    : num  278 265 266 266 263 265 267 268 267 267 ...
##  $ Turbidity : num  0.2 0.4 0.3 0.3 1.7 2.4 1.7 1.5 1.7 1.7 ...
##  $ DateTime  : POSIXct, format: "2024-10-22 10:30:50" "2024-10-22 10:45:50" ...
##  - attr(*, ".internal.selfref")=<externalptr>
```

Review imported file and if required, remove out-of-the-water-edits i.e. data before sensor was deployed in the river if sensor is running before being deployed, and data after sensor is removed from the river. Often the sensor will be put into operation to check everything is logging before being deployed in the water.

```
# When importing multiple files together you will need to do this for each file imp OW061024 <-
OW061024 %>% filter(DateTime > ymd_hms('2024-10-06 13:40:00'))
# and after removed from river
OW061024 <- OW061024 %>% filter(DateTime < ymd_hms('2024-10-21 17:20:00'))
OW221024 <- OW221024 %>% filter(DateTime > ymd_hms('2024-10-22 17:45:50'))
# and after removed from river
OW221024 <- OW221024 %>% filter(DateTime < ymd_hms('2024-11-05 11:46:50'))
# and similarly if the sensor was checked in situ during deployment
```

1.5 Combine the files into a single dataframe

To process the data as one file for visualization and event detection, the files need to be joined together.

```
# Bind OW061024 and OW221024 to form new object OWCombined
OWCombined<- bind_rows(OW061024,OW221024)
```

2.0 Data Integrity

2.1 Embed source data context for data integrity by adding variable labels.

To ensure the integrity of the data persists once imported into R, it is very useful to embed the source data context in the datafile. Embed the source data context by adding variable labels to the sensor data. In this case the site name and sensor details are embedded.

```
OWCombined<-OWCombined|>   labelled::set_variable_labels(     DateTime = "Measurement Date and Time of River Owenmore, Sligo ",
    Temp = "Temperature (degC) YSI6600EDSV2_2 6560 Conductivity_Temp. Probe",
    SpCond = "Specific Conductance uScm-1 YSI6600EDSV2-2 6560 Conductivity_Temp. Pr
    Turbidity = "Turbidity (NTU) - YSI_6600_EDS_V2-2 - 6136 Turbidity Probe"
  )
# To view the variable labels # A new tab opens displaying the combined dataframe.
View(OWCombined)
```

```
##          Date     Time  Temp SpCond Turbidity            DateTime
##        <char>   <char> <num>  <num>     <num>              <POSc>
## 1: 06/10/2024 13:45:50 13.31    486       3.2 2024-10-06 13:45:50
## 2: 06/10/2024 14:00:50 13.38    485       3.2 2024-10-06 14:00:50
## 3: 06/10/2024 14:15:50 13.43    485       3.2 2024-10-06 14:15:50
## 4: 06/10/2024 14:30:50 13.48    484       3.2 2024-10-06 14:30:50
## 5: 06/10/2024 14:45:50 13.53    484       2.8 2024-10-06 14:45:50
## 6: 06/10/2024 15:00:50 13.57    483       2.9 2024-10-06 15:00:50
```

2.2 Export the tidyied datafile as a .csv file and as .rds file to working directory

Note the datalabels are lost whenexporting the files as .csv but are maintained when exported as .rds file. This file is used for visualisation and event detection.

```
# Export the cleaned datafile as .csv to the working directory #
write.table(OWCombined,"./02_Embed_Source_Data_Context/Output_Files/OW061024_051124
# save the file as RData (useful if importing into R again)
saveRDS(OWCombined, file = "./02_Embed_Source_Data_Context/Output_Files/OW061024_05
```

3.0 Visualisation of Water Quality Data

3.1 Load the cleaned datasets into R studio

Load the YSI Sonde Dataset for the River Owenmore at Ballynacarrow

```
OW061024_051124C<-readRDS("./02_Embed_Source_Data_Context/Output_Files/OW061024_051
str(OW061024_051124C)
```

Classes 'data.table' and 'data.frame': 2775 obs. of 6 variables:
$ Date: chr "06/10/2024" "06/10/2024" "06/10/2024" "06/10/2024" ...
$ Time : chr "13:45:50" "14:00:50" "14:15:50" "14:30:50" ...
$ Temp : num 13.3 13.4 13.4 13.5 13.5 ... ## ..- attr(*, "label")= chr "Temperature (degC) YSI6600EDSV2_2 6560 Conductivity ## $ SpCond : num 486 485 485 484 484 483 483 483 482 482 ...
..- attr(*, "label")= chr "Specific Conductance uScm-1 YSI6600EDSV2-2 6560 Con ## $ Turbidity : num 3.2 3.2 3.2 3.2 2.8 2.9 3.2 3.3 3.5 3.2 ...
..- attr(*, "label")= chr "Turbidity (NTU) - YSI_6600_EDS_V2-2 - 6136 Turbidit ## $ DateTime : POSIXct, format: "2024-10-06 13:45:50" "2024-10-06 14:00:50" ...
- attr(*, ".internal.selfref")=<externalptr>

3.2 Visualise the Data using ggplot2 and ggplotly

Data is visualised which aids in understanding the data. There are two type of plots created: 1. Static plots which appear as an image can be viewed in the Plots tab and 2. Interactive plots where can be viewed in the viewer tab. With interactive plots, you can hover over specific data points and the value will be displayed, you can zoom in on an area of a plot by click and drag with the mouse, and zoom out completely by double clicking. Video resources are available online for ggplotly if you want to learn more.

3.2.1 Plot the Turbidity data

ggplot(OW061024_051124C,aes(DateTime, Turbidity))+ geom_point(size=0.5)+ xlab("Date")+ ylab("Turbidity (NTU)")+ scale_x_datetime(
labels = date_format("%d %b %Y"), breaks = date_breaks("2 days"))+ theme(axis.text.x = element_text(angle = 20))

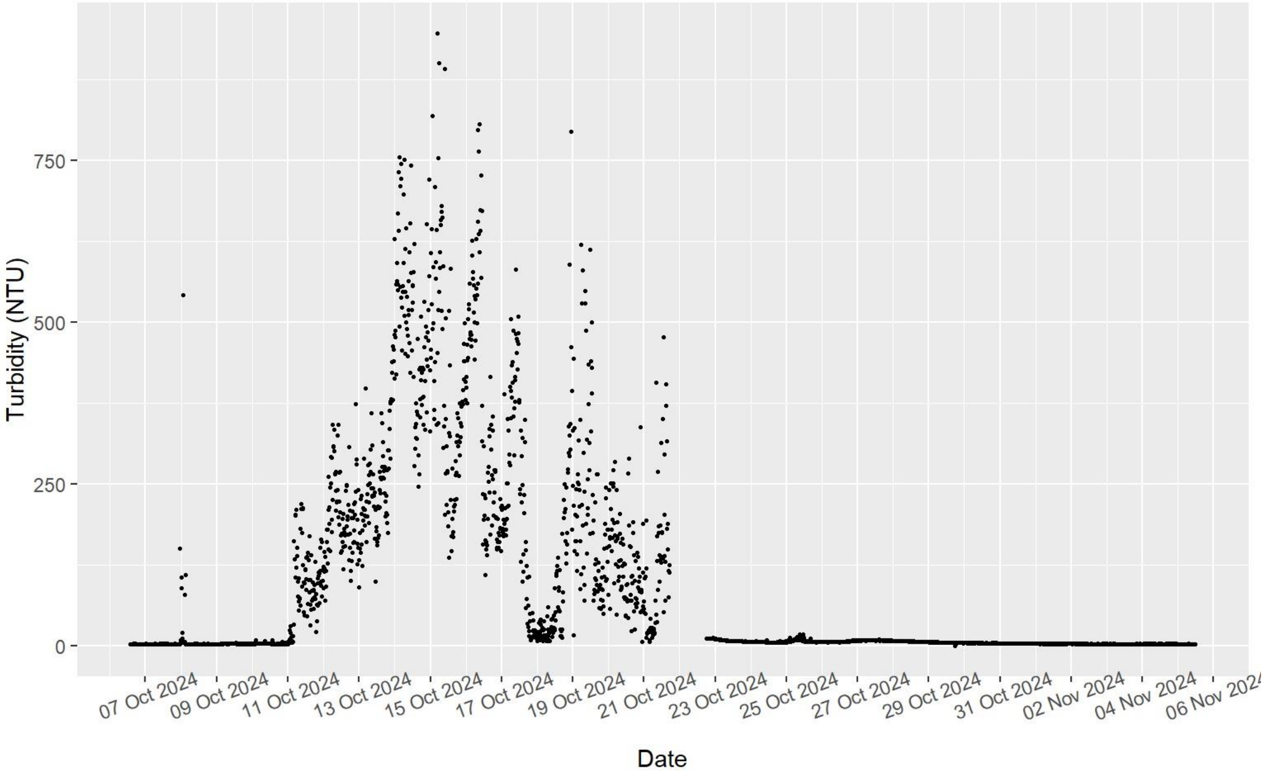

## Name the object Turbidity Plot for specific Data file OW061024_051124C_Turbidity_Plot<- ggplot(OW061024_051124C,aes(DateTime, Turbidity))+ geom_point(size=0.5)+ xlab("Date")+ ylab("Turbidity (NTU)")+ scale_x_datetime(labels = date_format("%d %b %Y"), breaks = date_breaks("2 days"))+ theme(axis.text.x = element_text(angle = 20))

Plot an interactive turbidity plot using plotly.

```
IntTurbidity_Plot<-ggplotly(OW061024_051124C_Turbidity_Plot)
IntTurbidity_Plot
```

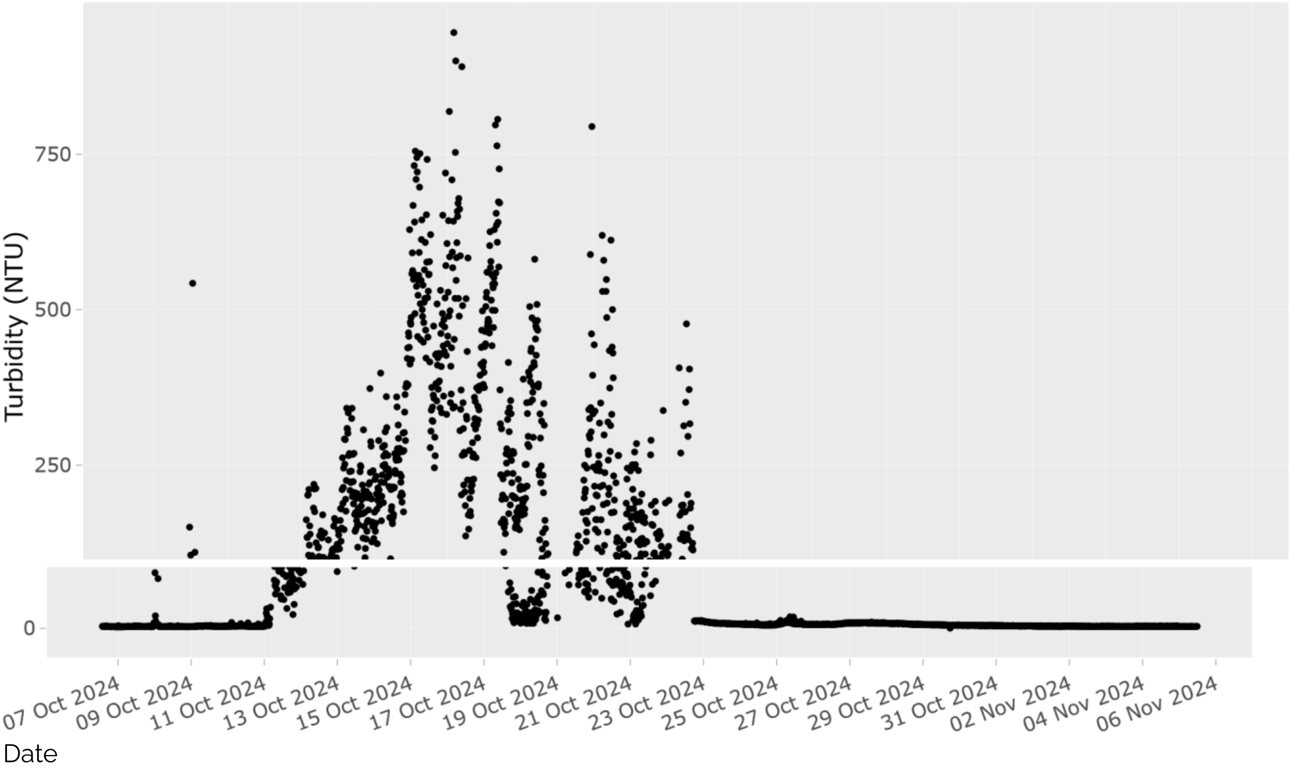

3.2.2 Plot the Specific Conductance data

ggplot(OW061024_051124C,aes(DateTime, SpCond))+geom_point(size=0.5)+ xlab("Date")+ylab("SpConductivity (uS/cm)")+ scale_x_datetime(labels = date_format("%d %b %Y"))+ theme(axis.text.x = element_text(angle = 20))

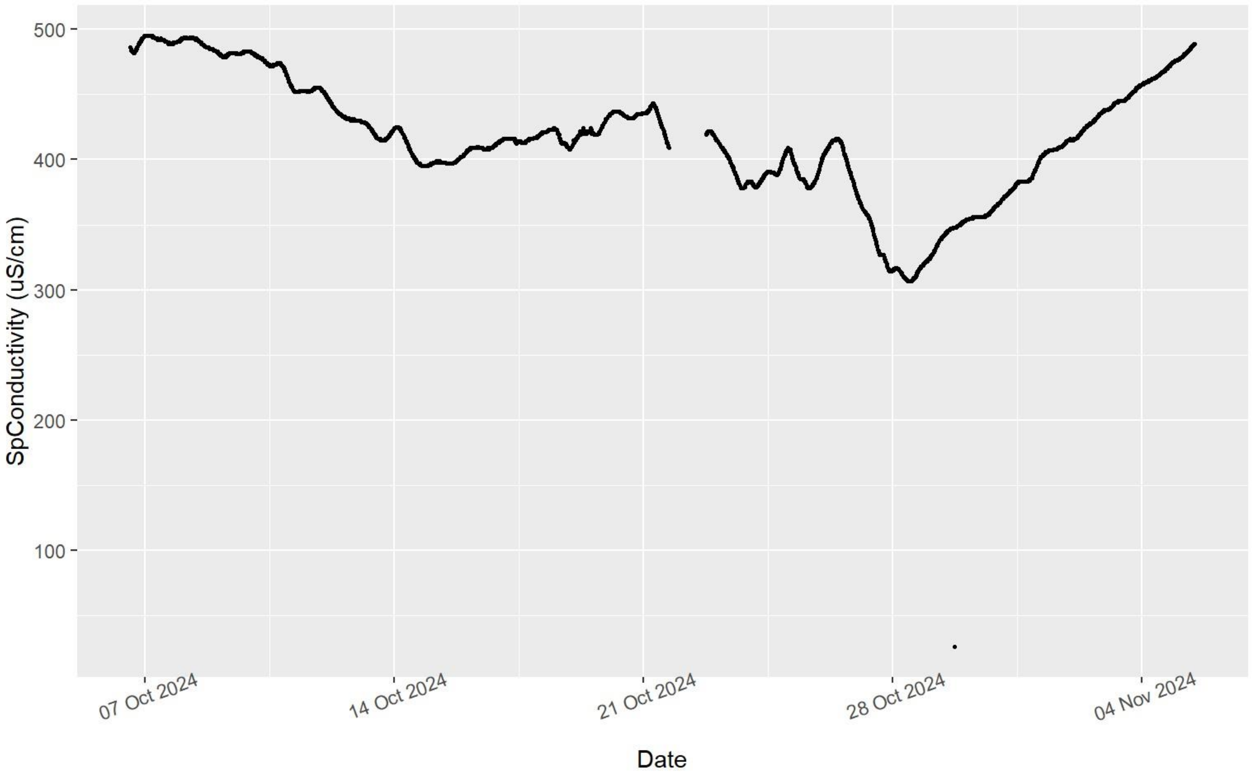

# Name the object SpCond for specific data file OW061024_051124C_SpCond_Plot<-
ggplot(OW061024_051124C,aes(DateTime, SpCond))+geom_p xlab("Date")+ylab("SpCond. (uS/cm)")+
scale_x_datetime(labels = date_format("%d %b %Y"))+ theme(axis.text.x = element_text(angle = 20))
OW061024_051124C_SpCond_Plot

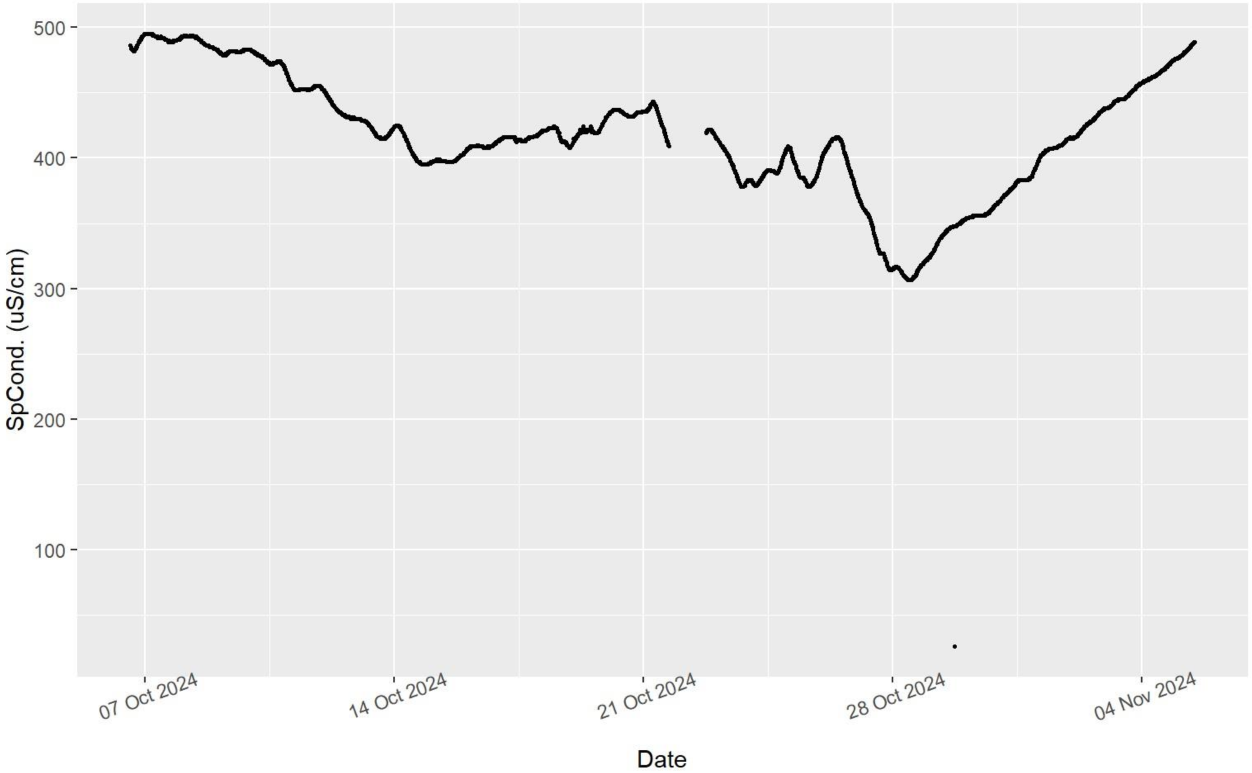

Interactive plot for specific conductance using plotly

IntSpCond_Plot<-ggplotly(OW061024_051124C_SpCond_Plot)
IntSpCond_Plot

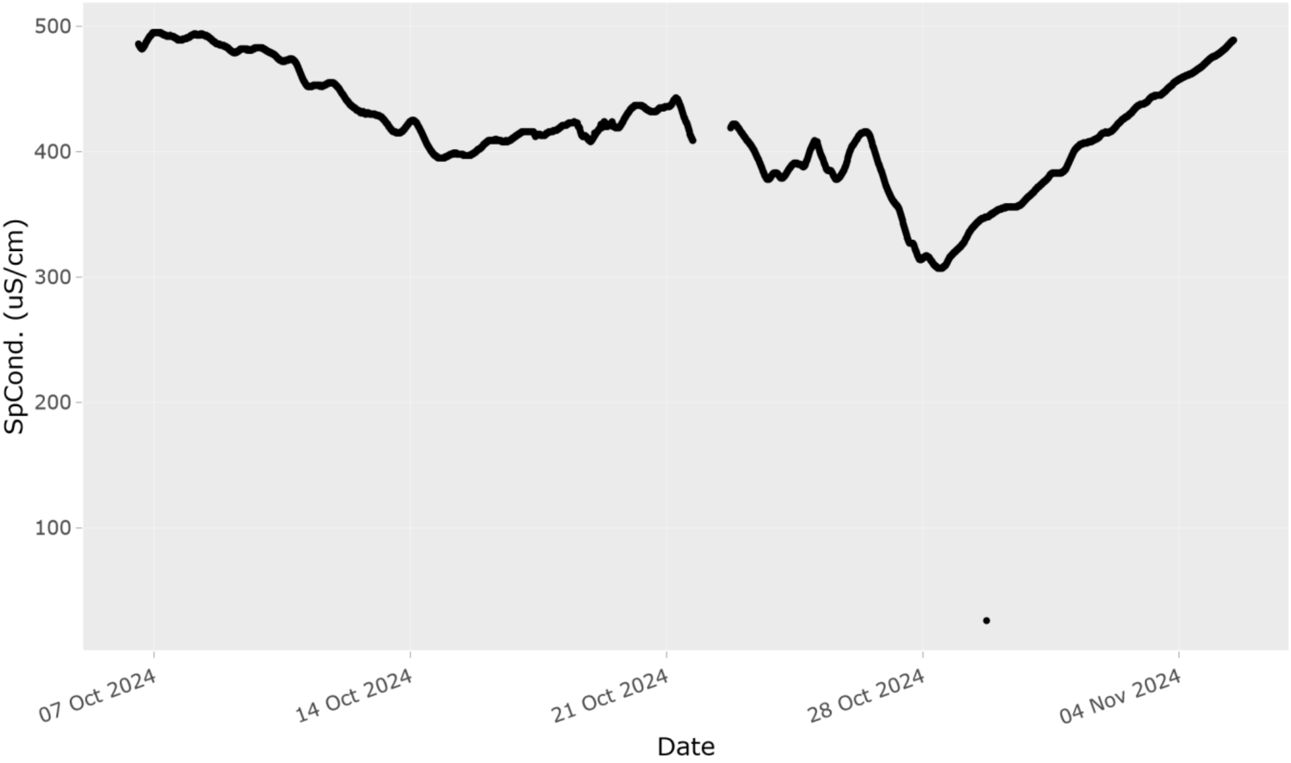

3.2.3 Plot the Temperature Data

OW061024_051124C_Temp_Plot<-ggplot(OW061024_051124C, aes(DateTime,Temp)) + geom_poi xlab("Date") + ylab(bquote(Temp. ("\u00B0C")))+ scale_x_datetime(labels = date_format("%d %b %Y"))+ theme(axis.text.x = element_text(angle = 20))+ ylim(0, 20)
OW061024_051124C_Temp_Plot

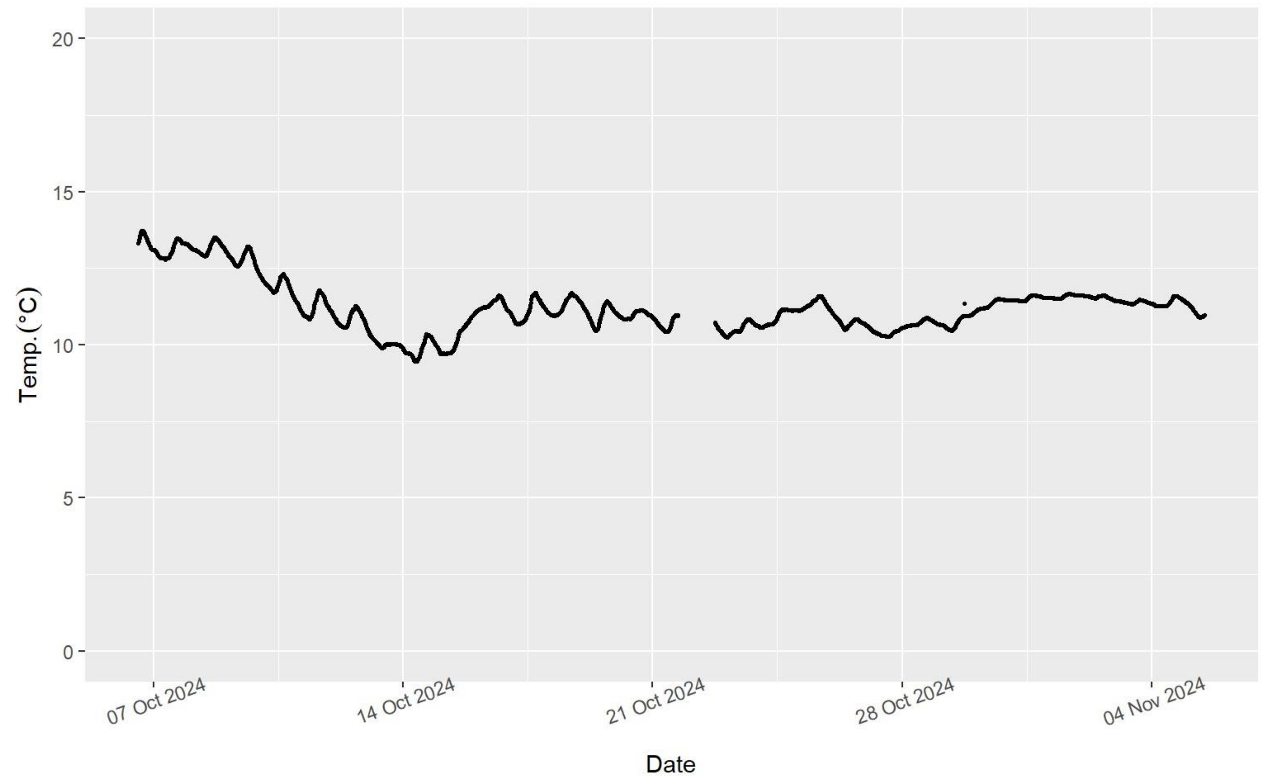

Plot the interactive temperature plot using plotly (plotly won't plot with customised symbols used for units, in this case the text 'degrees celsius' is used for the y axis label))

OW061024_051124C_Temp_PlotV1<-ggplot(OW061024_051124C, aes(DateTime,Temp)) + geom_p xlab("Date") + ylab("Temp. deg.C")+ scale_x_datetime(labels = date_format("%d %b %Y"))+ theme(axis.text.x = element_text(angle = 20))+ ylim(0, 20)
OW061024_051124C_Temp_PlotV1

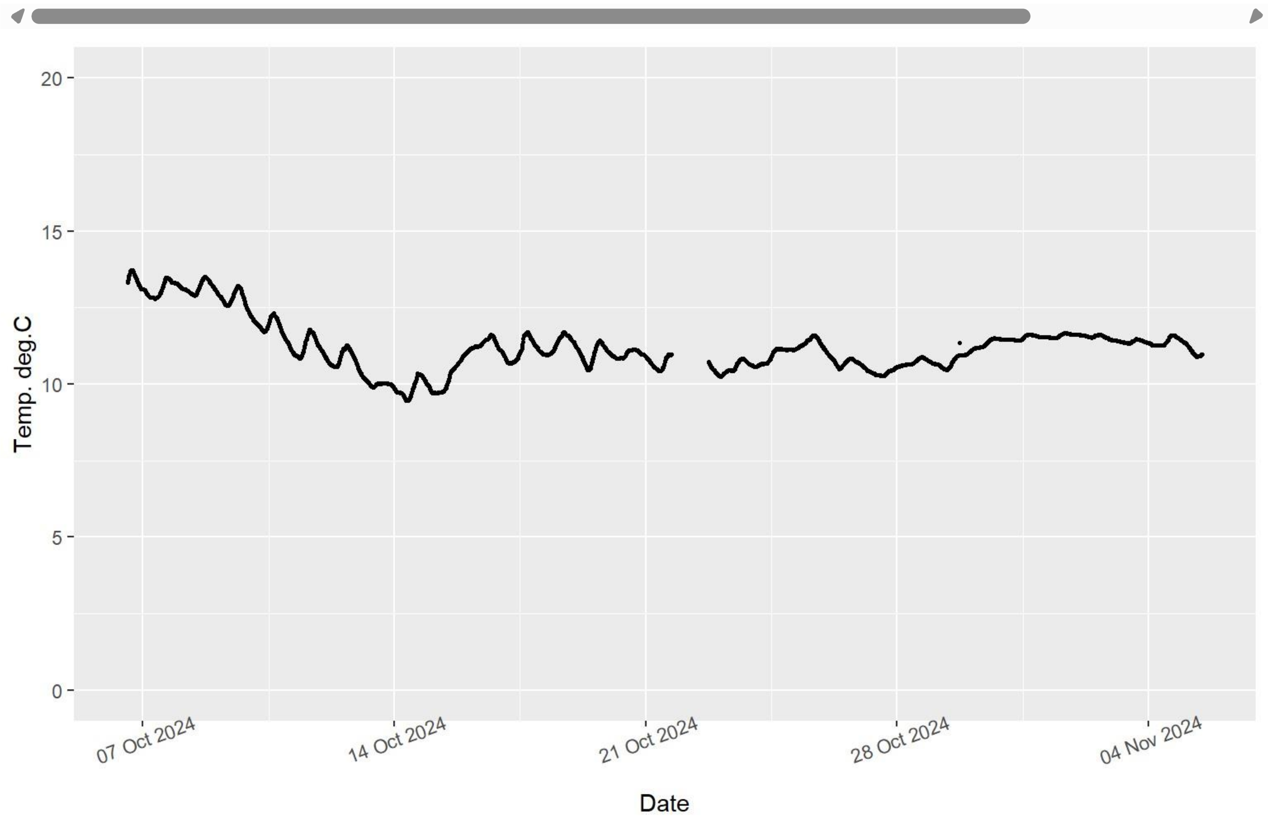

IntTemp_Plot<-ggplotly(OW061024_051124C_Temp_PlotV1)
IntTemp_Plot

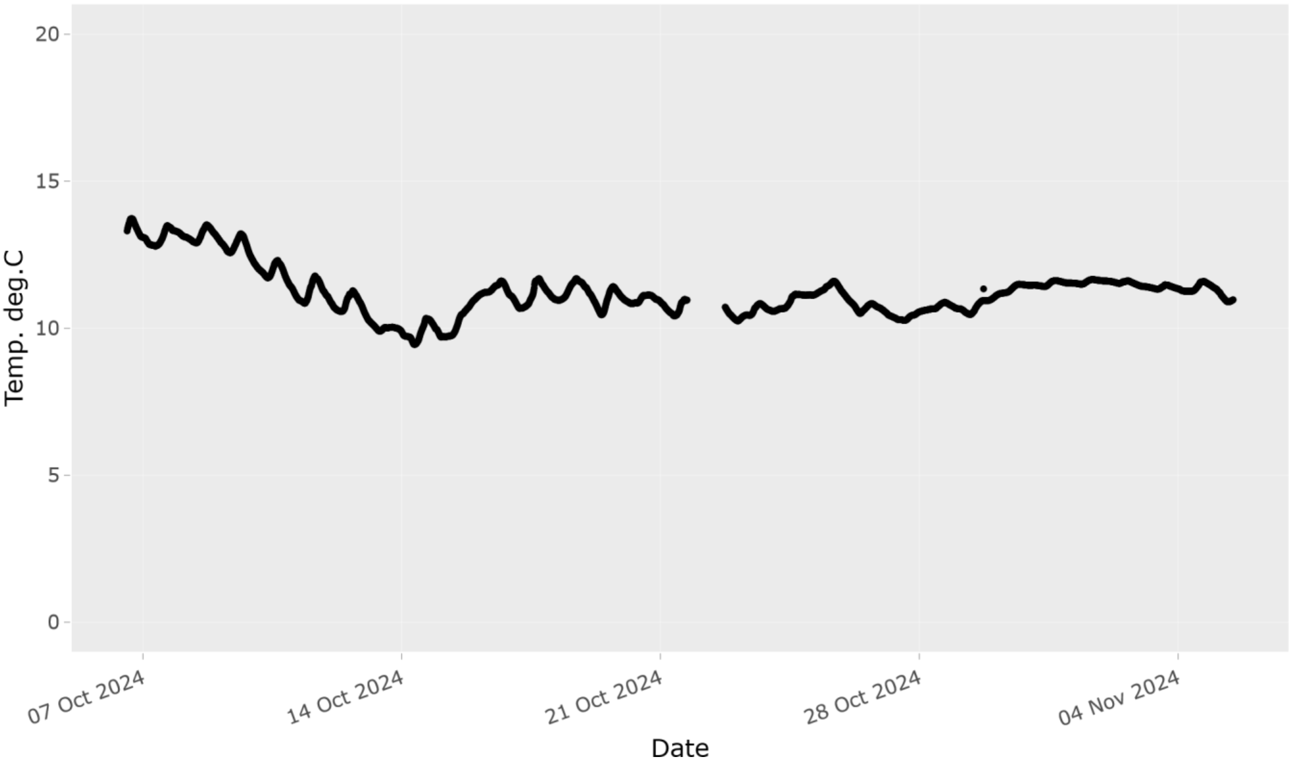

3.2.4 Plot the water quality data together in one plot

You can zoom in on a plot using zoom in the Plots tab. Arrange all plots in one column and align by xaxis

Comb_OW061024_051124C<-plot_grid(OW061024_051124C_Turbidity_Plot, OW061024_051124C_Temp_Plot, OW061024_051124C_S
Comb_OW061024_051124C

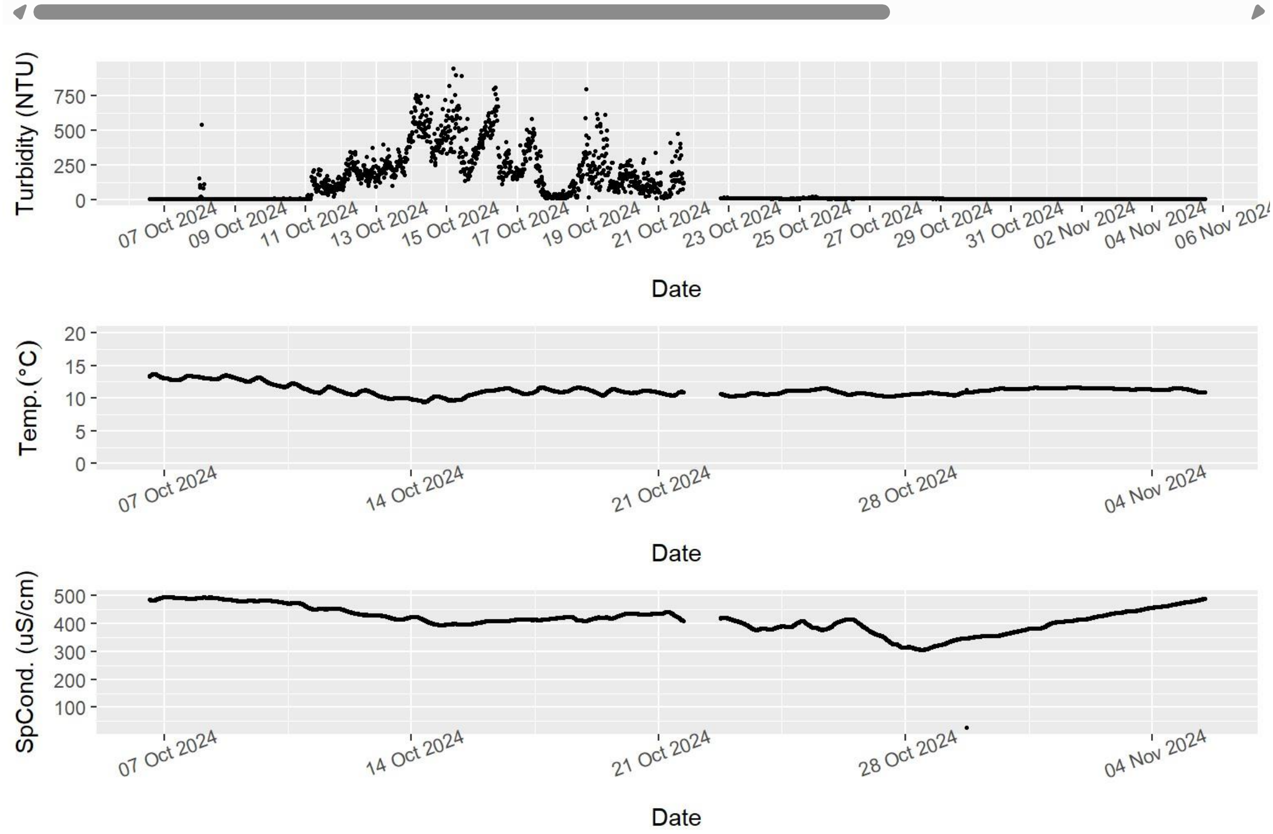

```
# To include a title on the plot
Comb_OW061024_051124CV1<-grid.arrange(OW061024_051124C_Turbidity_Plot,
                        OW061024_051124C_Temp_Plot, OW061024_051124C_SpCon
```

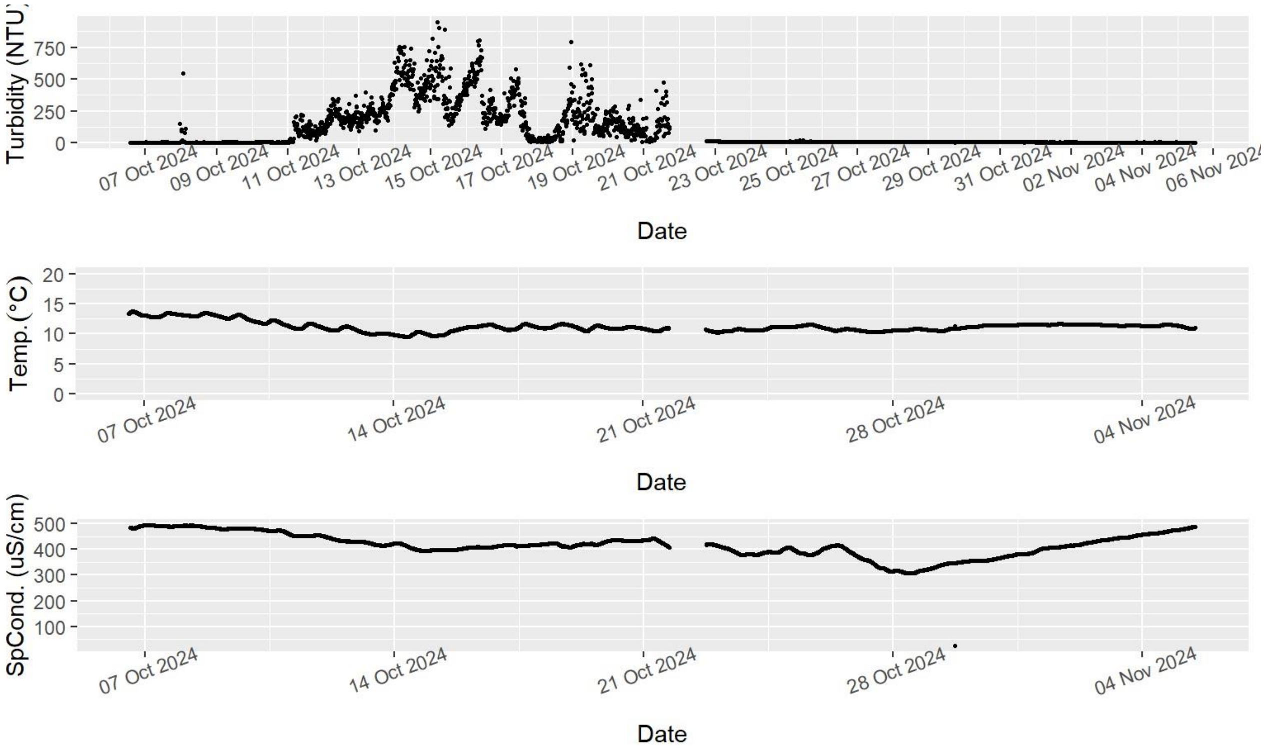

Figure 1: River Owenmore at Ballynacarrow

3.3 Save the Combined Plots

```
ggsave(here("./03_Data_Visualisation_of_TimeSeries_Data/Output_Files", "Comb_OW0610
```

```
## Saving 8 x 5 in image
```

4.0 Summary Statistics

4.1 Check for any missing values as it interferes with computing summary statistics and further analysis.

colSums(is.na(OW061024_051124C))
Date Time Temp SpCond Turbidity DateTime
0 0 0 0 0 0
# Find and count any missing values # number of missing values
which(is.na(OW061024_051124C$Turbidity)) ## integer(0)
which(is.na(OW061024_051124C$SpCond)) ## integer(0)
which(is.na(OW061024_051124C$Temp)) ## integer(0)
# location of missing values (if any) print("Position of missing values ") ## [1] "Position of missing values "
which(is.na(OW061024_051124C)) ## integer(0)

4.2 Screen for datapoints outside the measurement range of the sensors. The measurement range varies from sensor to sensor so it's important to refer to the measurement range for the particular sensors used.

Specific Conductance
# Identify where SpCond <0 uS/cm
OW061024_051124C_SpCond_lo<-OW061024_051124C$SpCond<0 *#count the number of TRUE, FALSE, and NA values in vector turbidity <0. If count 0*
summary(OW061024_051124C_SpCond_lo)

Mode FALSE
logical 2775
# Identify where SpCond>100,000 uS/cm
OW061024_051124C_SpCond_hi<-OW061024_051124C$SpCond>100000 *#count TRUE, FALSE, and NA values in vector turbidity >1000* summary(OW061024_051124C_SpCond_hi)
Mode FALSE
logical 2775
Export SpCond_OutRange Values to pdf file
pdf("04 Summary Statistics in R & Remove Outrange data/Output_Files/OW061024_051124
summary(OW061024_051124C_SpCond_lo)

Mode FALSE ## logical 2775
summary(OW061024_051124C_SpCond_hi)
Mode FALSE ## logical 2775
dev.off
function (which = dev.cur())
{
if (which == 1) ## stop("cannot shut down device 1 (the null device)")

```
##     .External(C_devoff, as.integer(which))
##     dev.cur()
## }
## <bytecode: 0x000001b12689b7e8>
## <environment: namespace:grDevices>
```
# in this case there are no values outside the measurement range so the pdf file is

Turbidity - YSI 6136 Turbidity Sensor Range 0 to 1000 NTU

# Identify where turbidity <0 NTU identify_rows_turbidity_lo<-
OW061024_051124C$Turbidity<0 *#count TRUE, FALSE, and NA values in vector turbidity <0*
summary(identify_rows_turbidity_lo)

```
##    Mode   FALSE
## logical    2775
```
# Identify where turbidity >1000NTU
identify_rows_turbidity_hi<-OW061024_051124C$Turbidity>1000 *#count TRUE, FALSE, and NA values in vector turbidity >1000* summary(identify_rows_turbidity_hi)

```
##    Mode   FALSE
## logical    2775
```
# If turbidity in the file is >1000NTU create a subset dataframe with these values.
subset(OW061024_051124C, Turbidity>1000)

```
## Empty data.table (0 rows and 6 cols):      Date,    Time,Temp,SpCond,Turbidity,
```

4.3 Replace All Data Points outside the Measuring Range of Sensor with NA and create new dataframe

Replace all turbidity values greater than sensor measuring range (>1000 NTU) with NA
OW061024_051124C_NoOutRange<- replace_with_na_at(OW061024_051124C, "Turbidity", ~ .

Replace all turbidity values less than the measuring range (<0 NTU) with NA
OW061024_051124C_NoOutRange<- replace_with_na_at(OW061024_051124C_NoOutRange, "Turb

Check datapoints have been replaced (both greater than and less than the measuring range)
identify_rows_turbidity_hi<-OW061024_051124C_NoOutRange$Turbidity>1000
summary(identify_rows_turbidity_hi)

```
##    Mode   FALSE ## logical    2775
identify_rows_turbidity_lo<-OW061024_051124C_NoOutRange$Turbidity<0
summary(identify_rows_turbidity_lo)
##    Mode   FALSE
## logical    2775
```

Save the edited dataframe to the working directory as .csv file

```
here::here() ## [1] "C:/Repos/Workflow-for-identifying-potential-water-quality-events-using-time
```

```
write.csv(OW061024_051124C_NoOutRange, "04 Summary Statistics in R & Remove Outrang
```

4.4 Summary Statistics

Produce a summary statistics table

```
df1<-as.data.frame(OW061024_051124C_NoOutRange)
data_summary<-stargazer(df1, type="text", title="River Owenmore, Sligo  06/10/24 to
covariate.labels = c("Temperature (deg.C)", "Specific Condu
summary.stat=c("n","mean", "sd","min", "median","max"),                     style="ajps",
out="04 Summary Statistics in R & Remove Outrange data/Outp
```

```
##
## River Owenmore, Sligo 06/10/24 to 05/11/24 (non-continuous)
## -----------------------------------------------------------------
## Statistic                    N    Mean  St. Dev. Min Median  Max
## -----------------------------------------------------------------
## Temperature (deg.C)          2775 11.2    0.9    9.4  11.1  13.7
## Specific Conductance (uS/cm) 2775 417.8   46.1   26   416    495
## Turbidity (NTU)              2775 93.1   158.9   0.3  6.9   945.8
## -----------------------------------------------------------------
```

Count the number of days when monitoring was carried out

```
n_distinct(OW061024_051124C_NoOutRange$`      Date`) ## [1] 31
CountDays<-n_distinct(OW061024_051124C_NoOutRange$`      Date`)
CountDays
## [1] 31
```

Count no. of observations

```
NumberRowsOW061024_051124C<-nrow(OW061024_051124C_NoOutRange)
NumberRowsOW061024_051124C
## [1] 2775
```

Record number of days and number of observations to an output file

```
sink("04 Summary Statistics in R & Remove Outrange data/Output_Files/OW061024_05112
CountDays NumberRowsOW061024_051124C sink()
```

5.0 Define significant change thresholds for turbidity and highlight on plots to identify events

5.1 Identification of Significant Change Thresholds for Turbidity

The turbidity timeseries data needs to be visually inspected to identify potential water quality events where there is a significant change in the turbidity from the baseline. To facilitate the visual identification (using ggplot2) of events from significant increases in water turbidity, a turbidity threshold value is calculated for water quality at the site. Sections of the plot greather than this turbidity threshold are highlighted and the data from this plot exported to a csv file. This csv file is used by the analyst to record the manually verified eventsand subsequenly comared to the potential water quality events identified by the CANARY software.

5.2 Visualise the data

Plot the turbidity data and remove NA values (Not Available data or missing values) as these affect how the code runs.

```
OW061024_051124C_Turbidity_Plot <- ggplot(OW061024_051124C_NoOutRange, aes(x =
Date   geom_point(na.rm = TRUE)+   scale_x_datetime(date_breaks = "1 week")+   labs(x =
"Time", y = "Turbidity (NTU)") +   theme_minimal()+
  theme(axis.text.x = element_text(angle = 45, hjust = 1)) OW061024_051124C_Turbidity_Plot
```

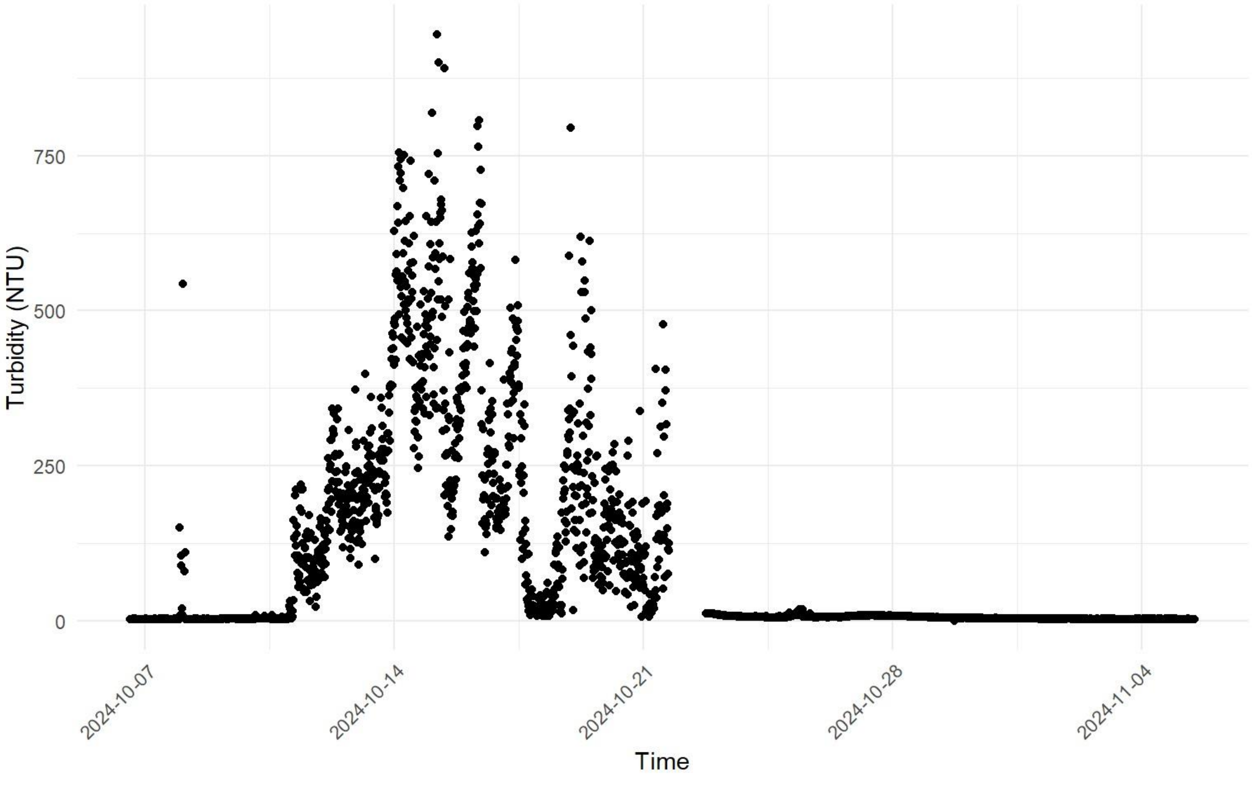

Save the plot to the working directory.

## Save the OW061024_051124C_NoOutRange_Turbidity_Plot to a pdf document pdf("05 SCT and highlight areas on plot for events/Output_Files/OW061024_051124C_Tu print(OW061024_051124C_Turbidity_Plot) dev.off()

```
## png
##   2
```

Plot an interactive turbidity plot.

Can be used to zoom in on areas of interest using the zoom function above the plot when examining potential water quality events.

```
OW061024_051124C_IntTurbPlot<- ggplotly(OW061024_051124C_Turbidity_Plot)
OW061024_051124C_NoOutRange
##          Date     Time  Temp SpCond Turbidity            DateTime
##        <char>   <char> <num>  <num>     <num>              <POSc>
##    1: 06/10/2024 13:45:50 13.31    486       3.2 2024-10-06 13:45:50
##    2: 06/10/2024 14:00:50 13.38    485       3.2 2024-10-06 14:00:50
##    3: 06/10/2024 14:15:50 13.43    485       3.2 2024-10-06 14:15:50
##    4: 06/10/2024 14:30:50 13.48    484       3.2 2024-10-06 14:30:50
##    5: 06/10/2024 14:45:50 13.53    484       2.8 2024-10-06 14:45:50
##   ---
## 2771: 05/11/2024 10:45:51 10.94    488       3.1 2024-11-05 10:45:51
## 2772: 05/11/2024 11:00:51 10.95    488       3.1 2024-11-05 11:00:51
## 2773: 05/11/2024 11:15:51 10.95    489       2.9 2024-11-05 11:15:51
## 2774: 05/11/2024 11:30:51 10.96    489       3.0 2024-11-05 11:30:51
## 2775: 05/11/2024 11:45:50 10.97    489       3.1 2024-11-05 11:45:50
```

5.3 Significant Change Threshold & Highlight potential water quality events on turbidity plots

This may be helpful for those unfamiliar with viewing time series plots. A statistical test, Tukey Fences test for outliers is used to identify potential turbidity outliers in the data, 26ccurring26 those high turbidity values 26ccurring at the upper range of the data as these can indicate possible water quality events. The identified outliers will be highlighted in red on the plot and an interactive plot of turbidity values is plotted. This allows the analyst to manually verify possible turbidity events which can be used for the initial CANARY configuration (training).

5.3.1 Tukey Fences test for outliers

Compute Inter Quartile Range (IQR) of the turbidity data. These values are displayed under 'Values' in the environment.

```
quantile(OW061024_051124C_NoOutRange$Turbidity, 0.25, na.rm=TRUE)
## 25%
## 3.5
```

```
Q1<-quantile(OW061024_051124C_NoOutRange$Turbidity, 0.25, na.rm=TRUE)
quantile(OW061024_051124C_NoOutRange$Turbidity, 0.75, na.rm=TRUE)
##   75%
## 135.6
Q3<- quantile(OW061024_051124C_NoOutRange$Turbidity, 0.75, na.rm=TRUE)
IQR<-Q3-Q1 print(IQR)
##   75% ## 132.1
multiplier <- 1.5
```

The upper bound and lower bound are the values of interest in terms of turbidity outliers

Calculate the Lower bound turbidity value

```
Q1-(IQR * multiplier)
##     25% ## -194.65
lower_bound<-Q1-(IQR * multiplier)
```

Calculate the upper bound turbidity value

```
Q3 + (IQR*multiplier)
##    75%
## 333.75
upper_bound<-Q3 + (IQR*multiplier)
```

5.3.2 Identify where the turbidity is likely to be an outlier.

An outlier is >/= upper bound in Tukey Fences. Create a new variable (column) called 'Alarm Classify' where values are categorised as 1 (True) i.e. >/= upper bound or 0 (False) i.e. </= upper bound.

Identify where Turbidity >/= upper bound (Turbidity Threshold Value) and in a new variable (column) called Event Alarm, classify as 1 (true) or 0 (false)

```
OW061024_051124C_TurbEvent<-OW061024_051124C_NoOutRange %>%
mutate(Possible_Owenmor   Turbidity > upper_bound, true = 1, false = 0))
head(OW061024_051124C_TurbEvent, 10)
```

```
##          Date     Time  Temp SpCond Turbidity          DateTime
##        <char>   <char> <num>  <num>     <num>            <POSc>
##  1: 06/10/2024 13:45:50 13.31    486       3.2 2024-10-06 13:45:50
##  2: 06/10/2024 14:00:50 13.38    485       3.2 2024-10-06 14:00:50
##  3: 06/10/2024 14:15:50 13.43    485       3.2 2024-10-06 14:15:50
##  4: 06/10/2024 14:30:50 13.48    484       3.2 2024-10-06 14:30:50
##  5: 06/10/2024 14:45:50 13.53    484       2.8 2024-10-06 14:45:50
##  6: 06/10/2024 15:00:50 13.57    483       2.9 2024-10-06 15:00:50
##  7: 06/10/2024 15:15:50 13.60    483       3.2 2024-10-06 15:15:50
##  8: 06/10/2024 15:30:50 13.66    483       3.3 2024-10-06 15:30:50
##  9: 06/10/2024 15:45:50 13.70    482       3.5 2024-10-06 15:45:50 ## 10: 06/10/2024
16:00:50 13.72    482       3.2 2024-10-06 16:00:50
##     Possible_Owenmore_Event_Alarm
##                             <num>
##  1:                             0
```

```
## 2:                        0
## 3:                        0
## 4:                        0
## 5:                        0
## 6:                        0
## 7:                        0
## 8:                        0
## 9:                        0 ## 10:                        0
str(OW061024_051124C_TurbEvent)
## Classes 'data.table' and 'data.frame':   2775 obs. of  7 variables:
## $      Date                : chr  "06/10/2024" "06/10/2024" "06/10/2024" "0 ## $     Time
: chr  "13:45:50" "14:00:50" "14:15:50" "14:30:5 ## $ Temp                   : num  13.3 13.4 13.4 13.5
13.5 ...
##   ..- attr(*, "label")= chr "Temperature (degC) YSI6600EDSV2_2 6560 Conductivity ## $
SpCond                   : num  486 485 485 484 484 483 483 483 482 482 .
##   ..- attr(*, "label")= chr "Specific Conductance uScm-1 YSI6600EDSV2-2 6560 Con ## $
Turbidity                : num  3.2 3.2 3.2 3.2 2.8 2.9 3.2 3.3 3.5 3.2 .
## $ DateTime                   : POSIXct, format: "2024-10-06 13:45:50" "2024-1 ## $
Possible_Owenmore_Event_Alarm: num  0 0 0 0 0 0 0 0 0 0 ...
##  - attr(*, ".internal.selfref")=<externalptr>
```

5.3.3 Count the number of observations where the turbidity >/= upper bound and express as a percentage of the total number of turbidity values in the data frame.

Count the number of True (1) values i.e. where turbidity >/= upper bound

```
print(Number_True_values<-length(which(OW061024_051124C_TurbEvent
$Possible_Owenmor
## [1] 275
```

Count the number of observations (i.e. number of values for each parameter in the data frame. This is also the number of rows in the dataframe.)

```
print(Number_observations<-nrow(OW061024_051124C_NoOutRange))
## [1] 2775
```

Calculate the percentage of values in dataframe where turbidity >/= upper bound

```
print(Percentage_turb_upperbound<-(Number_True_values/Number_observations)*100)
## [1] 9.90991
```

5.3.4 Export as .csv file the observations where Turbidity >/= upper bound. This file contains the observations suspected of being a possible turbidity water quality event.

Export OW061024_051124C_NoOutRange_TurbEvent as a .csv file

```
OW061024_051124C_TurbEvent$DateTime<-
format(OW061024_051124C_TurbEvent$DateTime) #
write.csv(OW061024_051124C_TurbEvent, "05 SCT and highlight areas on plot for event
```

5.3.5 Highlight significant change turbidity thresholds in red on plots

The code for this section is edited for this dataframe (Edward, 2024)

To help with visualising the turbidity values >/= upper bound (>/= Turbidity Threshold) on a plot, these values are highlighted on the plot as red lines.

```
Owenmore_Turbidity_Threshold_Plot<- ggplot(OW061024_051124C_NoOutRange, aes(x =
Dat   geom_point(na.rm = TRUE)+
  geom_rect(data = subset(OW061024_051124C_NoOutRange, Turbidity >=upper_bound),
aes(xmin = DateTime-450, xmax = DateTime+450, ymin = -Inf, ymax = Inf),           fill = "red",
alpha = 0.3) +   labs(x = "Time", y = "Turbidity (NTU)") +   theme_minimal()
Owenmore_Turbidity_Threshold_Plot
```

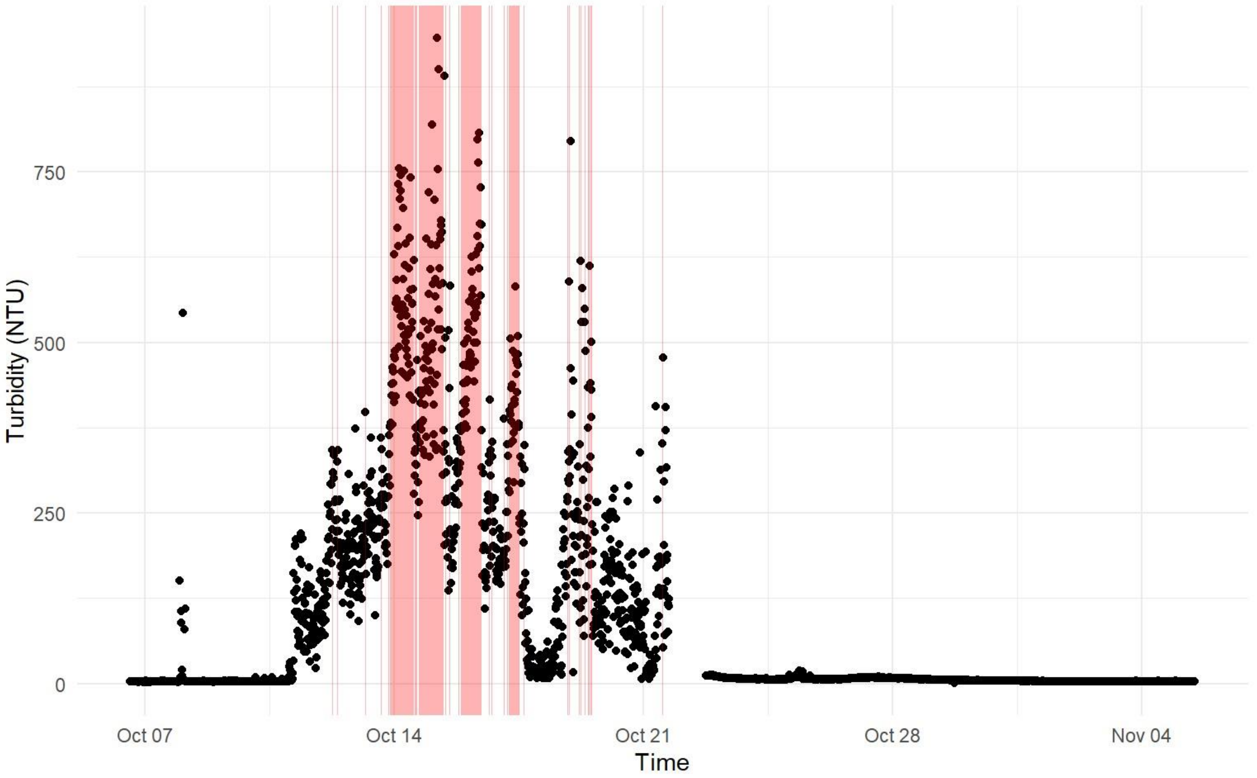

5.3.6 Interactive plot with highlighted areas on plot where turbidity is greater than the turbidity threshold value.

Interactive plots are very useful for interrogating data and this interactive plot may aid in the identification of probable turbidity events for users unfamiliar with time series datasets.

Users can zoom in and out of the plots. Use magnifying glass icon to open the plot full screen and click and drag the mouse to zoom in on an area, double click to zoom back out. The code for this section is edited for this dataframe (Kat, 2024).

Plot an interactive plot for turbidity with turbidity values >/= upper bound (>/= Turbidity Threshold) highlighted on the plot as red lines.

```
Fixer <- function(plt) {
  plt <- plotly_build(plt)            # make sure entire plot built   lapply(1:length(plt$x$data), \(k) {
# go through each trace (layer)     plt$x$data[[k]]$x <<- as.POSIXct(plt$x$data[[k]]$x) # update
x-axis data type   })   plt } tSpan <- function(gplt) {  # gplt: ggplot graph to be made into a
ggplotly   #  - assuming one x-axis within gplt
  #  - assuming geom_rect called 2nd in gplt
    # 2 here, because geom_rect was called 2nd           --- get data from plot   dta <-
data.frame(ggplot_build(gplt)$data[[2]][c("xmin", "xmax", "fill", "alpha")     mutate(xmin =
as.POSIXct(xmin), xmax = as.POSIXct(xmax))     shps <- map(1:nrow(dta), \(k) {    # create data
used in geom_rect
    list(type = "rect", xref = "x", yref = "paper",  # define shape, use x axis, no          x0 = dta$xmin[k],
x1 = dta$xmax[k],         # where on x          y0 = 0, y1 = 1,                        # where on plot (versus
y)          # update aesthetics         fillcolor = dta$fill[1], opacity = dta$alpha[1], line = list(width =
0))   })     ggplotly(gplt) %>%              # pre xaxis for dates     layout(xaxis = list(tickmode = "auto",
type = "date", autorange = T),           shapes = NA) %>%          # remove migration garbage
fixer() %>%                    # fix dates – make them dates again     layout(shapes = shps)             #
add new shapes } tSpan(Owenmore_Turbidity_Threshold_Plot)
```

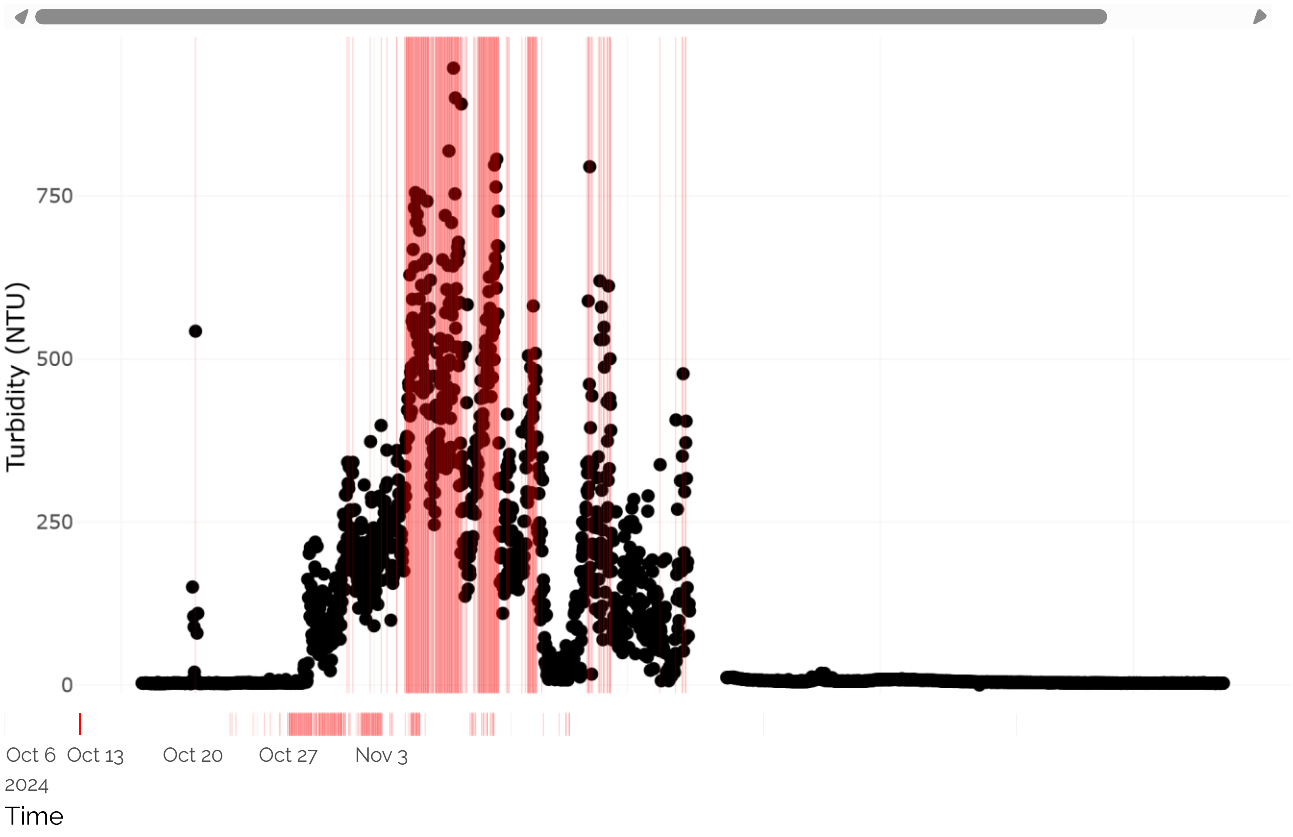

6.0 Manually Identify Potential Water Quality Events with the aid of the interactive turbidity plot.

6.1 Manually identify potential water quality events.

The analyst manually identifies potential water quality events by visual inspection i.e. looking at changes in the trend in the turbidity plot.
The highlighted areas on the interactive turbidity plot for events can be used as an aid to this manually identification as they highlight values where the Turbidity value is >/= upper bound of the Tukey Fences test for outliers. The highlighted data points are recorded in the 'Possible_Owenmore_Event_Alarm'column of the file 'OW061024_051124C_NoOutRange_TurbEvent' file and turbidity values at each timestep are recorded as either (true) if the Turbidity value >/= upper bound of the Tukey Fences test for outliers, or 0 (false) if not.
The highlighted areas can help identify potential events by drawing our attention to that area of the plot. However, data points either side of these highlighted areas may also indicate events and it is for the analyst viewing the data to determine whether a datapoint is a potential outlier by following the procedure outlined below.

The file 'OW061024_051124C_NoOutRange_TurbEvent' was copied by the analyst and renamed 'OW061024_051124C_NoOutRange_ManVer_TurbEvent.csv'. Note there is a gap in this data set between 21/10/2024 17:15:00 and 22/10/2024 18:00:00 when the sonde was removed from the river to the laboratory for service and calibration. An additional column 'Verified_OW_Event_Alarm' is manually added by the analyst to 'OW061024_051124C_NoOutRange_ManVer_TurbEvent.csv' file. The analyst manually records at each timestep whether they think the turbidity value is a potential alarm by inserting either 1 (true) or 0 (false) into 'Verified_OW_Event_Alarm' column based on visually examining the interactive turbidity plot in R which is produced in step 5.3.6 Interactive plot with highlighted areas on plot where turbidity is greater than the turbidity threshold value. The analyst may identify values either side of the highlighted areas on the plot as potential events and should insert 1 (true) at each timestep where this occurs. Once completed by the analyst, the 'OW061024_051124C_NoOutRange_ManVer_TurbEvent.csv' file is saved in the Man_Ver_Alarm folder in '06 Manually Identify Potential Water Quality Events/Output_Files' folder.

7.0 Event Detection using EPA CANARY

The software used for event detection was EPA CANARY-EDS. It is available on GitHub https://github.com/USEPA/CANARY (https://github.com/USEPA/CANARY) and includes links to download the software, and a comprehensive CANARY user manual, a quick start guide and tutorials to practice running the code. The EPA CANARY software will need to be downloaded and the OW061024_051124C_NoOutRange.csv file run through the CANARY

software. The water quality parameters analysed in EPA CANARY were Temperature, Specific Conductivity and Turbidity.

Four parameters in CANARY were varied to train the EPA CANARY software to identify probable water quality events in the dataset, with the aim of minimising the likelihood of false alarms. The parameters were history window, outlier threshold, BED window and event threshold. These parameters in CANARY were varied as per the method used in monitoring the Smith Branch watershed, Columbia, United States with the method to vary these parameters described in Section 3.2 CANARY event detection software and detailed in Table 3 (Burkhardt *et al.*, 2022). Rainfall events were not filtered for the analysis on the sample file from the Owenmore River, Ireland.

The output for step 7 event Detection using EPA CANARY is saved in 07 EPA CANARY Event Detection

Folder consisting of 96 .csv output files in six different folders. Once the data file OW061024_051124C_NoOutRange was processed in CANARY the output from the CANARY Data files for each algorithm were graphed monthly with probability, and then converted to .csv files in CANARY.

8.0 Compare manually identified Owenmore turbidity events to events identified by CANARY

This section of the code combines the manually identified events by the user with the output for each of the 96 algorithm files produced by CANARY into one dataframe, creating 96 new .csv files. The files are saved into six directories (folders) in the same file format as the CANARY output with the file name structure: sitedate_algorithm type_algorithm details_algorithm number. These files are saved in the directory associated with that data which is specified by the user by amending the code for the identifier in section 8.12. In this example, the data to be processed was placed in the directory called Data1 and the identifier set as follows #data_set_identifier<-“Data1/”. Where mutiple datasets are to be processed the user places each dataset in its own directory, e.g. Data1, Data2, Data3 etc and updates the code for data_set_identifier when running each dataset. The code automatically creates a directory in Output_Files for each dataset specified and ran through the code e.g. Output_Files/Data1, Output_Files/Data2 etc.

The output for step 8 comparing manually identified and CANARY events is saved in 08 Manually compare events to CANARY events/Output_files.

8.1 Specify the directory where the “manually verified event data” in located.

8.1.1 The path for this directory should be given relative to the project directory.

```
# Set the top directory for this step
ManVer_file_top_directory<-"06 Manually Identify Potential Water Quality Events/Out
```

8.1.2 This identifier is used to select the "manually verified event dataset" to be processed.

data_set_identifier<-"Data1/"

Sets up the full path to the "manually verified event data".

ManVer_file_directory<-paste0(ManVer_file_top_directory,data_set_identifier) (ManVer_file<-list.files(ManVer_file_directory,pattern='ManVer_TurbEvent'))

[1] "OW061024_051124C_ManVer_TurbEvent.csv"

ManVer_file_full_path<-paste0(ManVer_file_directory,ManVer_file)

8.2 Load the "manually verified event data" and format the dataframe.

Load the "manually verified event data".

ManVer_TurbEvent<-fread(ManVer_file_full_path,header=T,stringsAsFactors = F, select str(ManVer_TurbEvent)

```
## Classes 'data.table' and 'data.frame':   2775 obs. of  3 variables:
##  $ DateTime             : chr  "06/10/2024 13:45" "06/10/2024 14:00" "06/10/20 ##  $ Turbidity             : num  3.2 3.2 3.2 3.2 2.8 2.9 3.2 3.3 3.5 3.2 ...
##  $ Verified_OW_Event_Alarm: int  0 0 0 0 0 0 0 0 0 0 ...
##  - attr(*, ".internal.selfref")=<externalptr>
```

Change DateTime from chr to Date (as.POSIXct stores both a date and time with an associated time zone)

ManVer_TurbEvent$DateTime<-as.POSIXct(ManVer_TurbEvent$DateTime, format = "%d/%m/%Y str(ManVer_TurbEvent)

```
## Classes 'data.table' and 'data.frame':   2775 obs. of  3 variables:
##  $ DateTime             : POSIXct, format: "2024-10-06 13:45:00" "2024-10-06 1 ##  $ Turbidity : num  3.2 3.2 3.2 3.2 2.8 2.9 3.2 3.3 3.5 3.2 ...
##  $ Verified_OW_Event_Alarm: int  0 0 0 0 0 0 0 0 0 0 ...
##  - attr(*, ".internal.selfref")=<externalptr>
```

Rename to TIME_STEP to match the Owenmore CANARY File

ManVer_TurbEvent<- ManVer_TurbEvent%>% rename(TIME_STEP = DateTime)

8.3 Get list of directory names in the folder "07 EPA CANARY Event Detection"

Each directory is associated with a different configuration of CANARY.
Within each directory are output files generated by various versions of the algorithm.
The path for top_dir should be given relative to the project directory.

output_file_top_directory<-"07 EPA CANARY Event Detection/Output_Files/"
output_file_full_path<-paste0(output_file_top_directory,data_set_identifier)

List directories but not sub-directories.

(directory_list<-list.dirs(output_file_full_path,recursive=FALSE))

```
## [1] "07 EPA CANARY Event
Detection/Output_Files/Data1/Owenmore_LPCF_BED10_ET0.98
## [2] "07 EPA CANARY Event
Detection/Output_Files/Data1/Owenmore_LPCF_BED10_ET0.99
## [3] "07 EPA CANARY Event
Detection/Output_Files/Data1/Owenmore_LPCF_BED6_ET0.890
## [4] "07 EPA CANARY Event
Detection/Output_Files/Data1/Owenmore_LPCF_BED6_ET0.984
## [5] "07 EPA CANARY Event
Detection/Output_Files/Data1/Owenmore_LPCF_BED8_ET0.964
## [6] "07 EPA CANARY Event
Detection/Output_Files/Data1/Owenmore_LPCF_BED8_ET0.996
```

```
ID_String<-vector(mode="character",length=length(directory_list)) for (i in
1:length(directory_list)){
  ID_String[i]<-paste0("_L",sub(".*_L","",directory_list[i]))
}
```

8.4 Load the Owenmore CANARY file from 07 EPA CANARY

Event Detection Folder

```
# Now, step through each folder and locate the csv files for each algorithm. # Loop over the
directories for (i in 1:length(directory_list)){    # List all files in each parent directory, in turn.
(files_list<-list.files(directory_list[i],pattern='alg'))   #list all files in sp   alg_ver<-
vector(mode="character",length=length(files_list)) # initialise two char
Output_filename_string<-vector(mode="character",length=length(files_list))
# Loop through each file in the files_list    for (j in 1:length(files_list)){
    # Put together the input filename and directory it is in.
  input_filename_string<-paste0(directory_list[i],'/',files_list[j])
  # Read each of the "CANARY Event Detection" data files into a dataframe called
Input_Data<-fread(input_filename_string, header=T, stringsAsFactors = F,                select =
c("TIME_STEP","P_Event"))
    # Put together string name for output file.
    # First get the "algorithm version from each filename.
  # It's found by taking the part of the filename after the word "details" withou
  alg_ver[j]<-str_sub(sub(".*details-","",files_list[j]), end=-5)       # Setup the output filename.
  #(output_filename_string<-paste0("CANARYOwenmore",ID_String[i],alg_ver))

  #str(Input_Data)

  #Change TIME_STEP from chr to Date
  Input_Data$TIME_STEP<-as.POSIXct(Input_Data$TIME_STEP, format = "%d/%m/%Y
%H:%M
```

```
  # Change any negative values in CANARYOwenmoreAlg1 P_Event to absolute values t
  Input_Data$`P_Event`<-abs(Input_Data$`P_Event`)


  # A column containing the "manually verified event data" is added to the "CANAR     #... data
frame called "Combined Data".

  Combined_Data<-merge(Input_Data,ManVer_TurbEvent,by="TIME_STEP")

  #(which(is.na(Input_Data), arr.ind=TRUE))

  # View the first few rows and the structure of the combined dataframe
  #head(Combined_Data)
  #str(Combined_Data)
    # Export this data table as.csv file     here() # indicates the working directory
Combined_Data$TIME_STEP<-format(Combined_Data$TIME_STEP) # this prevents 00:00:

    # Now setup directory and filename for saving the combined data file.

  Output_directory_string<-paste0("08 Manually compare events to CANARY events/Ou
  Output_subdirectory_string<-paste0("Output_Files_",ID_String[i],"/")
  Output_directory_string_complete<-paste0(Output_directory_string,Output_subdire

  # Extract the location and date range information from the "manually verified e     # i.e, take
the first part of the filename before the "_ManVer_TurbEvents...."

  Location_and_Date_Range_ID_String<-sub("\\_M.*","",ManVer_file)

  Output_filename_string[j]<-paste0(Location_and_Date_Range_ID_String,ID_String[i

  (Output_file_path_and_name<-paste0(Output_directory_string,Output_subdirectory_
if (!dir.exists(Output_directory_string_complete)) dir.create(Output_directory_
write.csv(Combined_Data,Output_file_path_and_name, row.names=FALSE)    if (j==1){     # Only
print this message once per processed directory.
  cat("Writing data to file:\n",Output_file_path_and_name,"\n")}    }
}
## Writing data to file:
##  08 Manually compare events to CANARY events/Output_Files/Data1/Output_Files__LP
## Writing data to file:
##  08 Manually compare events to CANARY events/Output_Files/Data1/Output_Files__LP
## Writing data to file:
##  08 Manually compare events to CANARY events/Output_Files/Data1/Output_Files__LP
## Writing data to file:
```

```
##  08 Manually compare events to CANARY events/Output_Files/Data1/Output_Files__LP
## Writing data to file:
##  08 Manually compare events to CANARY events/Output_Files/Data1/Output_Files__LP
## Writing data to file:
##  08 Manually compare events to CANARY events/Output_Files/Data1/Output_Files__LP
```

9.0 Compare the Owenmore CANARY files to the manually identified events to identify the most suitable CANARY algorithm.

This is the last step in the workflow where the events identified are compared in order to identify the most suitable algorithm for that particular site based on the data provided to 'train' CANARY. This workflow used changes in the turbidity signal to identify potential water quality events at these two river stations as the turbidity values for the monitoring sites examined did not show a diurnal trend and baseline levels for turbidity at both sites was low. The identification of full and partial matches was carried out by analysing the output of step 8 where the output files of CANARY for each algorithm file were combined with manually identified event file. Events were classified as FULL or PARTIAL matches depending on whether the event time start timestep or time end timestep matched fully or partially.A description of the logic detailing how full and partial matches were classified is available in the Supplementary Material directory. Using the classification for matches the combined files (output for step 8) were analysed for matches by looping over each file to find where time steps either fully or partially matched for identified events. This was done in a step by step fashion to allow the user to verify that this code is correctly matching events for their data. The logic for how the files were matched is contained in the 'Code Logic for Finding Matches between manually verified events and EPA events' in the supplementary material folder.

Determine the number of matches and identify the most suitable CANARY algorithm.

9.1 Identify the folder for processing

Here, enter the directory containing the folders for processing

```
top_directory<-paste0("08 Manually compare events to CANARY events/Output_Files/",d
# List directories but not sub-directories.
(directory_list<-list.dirs(top_directory,recursive=FALSE))
```

```
## [1] "08 Manually compare events to CANARY events/Output_Files/Data1/Output_Files
## [2] "08 Manually compare events to CANARY events/Output_Files/Data1/Output_Files
## [3] "08 Manually compare events to CANARY events/Output_Files/Data1/Output_Files
## [4] "08 Manually compare events to CANARY events/Output_Files/Data1/Output_Files
## [5] "08 Manually compare events to CANARY events/Output_Files/Data1/Output_Files
## [6] "08 Manually compare events to CANARY events/Output_Files/Data1/Output_Files
```

# Only take the directory names which include the string "Output_Files" directory_list<-
directory_list[grepl("Output_Files",directory_list)]
# Set up dataframe for output.
Algorithm_Scores<-data.frame(algorithm=character(),FULL_MATCH=integer(),PARTIAL_MAT

9.2 Loop over all the directories for processing.

for (k in 1:length(directory_list)){ *# List all files in each parent directory, in turn.*
# Only consider filenames that have the string 'alg' in them.

(files_list<-list.files(directory_list[k],pattern='alg'))
# Loop over all the files in the current directory.
for (j in 1:length(files_list)){ *# Put together the directory path and filename for the current file to be proce* input_filename_string<-paste0(directory_list[k],'/',files_list[j]) dat<-read.csv(input_filename_string, header=T, stringsAsFactors = F) *# loading*

# Compare the Owenmore Turb Events to CANARY algorithm to determine the number
# The method requires an extra row at the top and bottom of the dataframe. *# Add row of 0s at start of dataframe.* dat<-rbind(0,dat) *# Add row of 0s at end of dataframe.* dat<-rbind(dat,0) *#Initialise variables to 0*
dat<-cbind(dat,Ver_Alarm_Start=0,Ver_Alarm_End=0) *#Initialise variables to 0* dat<-cbind(dat,CANARYTurb_Alarm_Start=0,CANARYTurb_Alarm_End=0) *#Initialise variables to 999*
dat<-cbind(dat, FULL_MATCH=999,PARTIAL_MATCH=999)

# Before doing the match, check for any na values in the dataframe, there *# if NA is present the code returns where NA occurs in the dataframe.* dat[which(rowSums(is.na(dat[,-1]))>0),]
## Also, ensure that there is a least 1 alarm present in the ##"Verified_OW_Event_A
contains_one<-any(dat$Verified_OW_Event_Alarm==1) if(!contains_one){ stop("There are no Verified Alarms in the data, nothing to do!")
}
# Any records containing NA values should be checked and must be removed for th

# The code will iterate over rows of the dataframe starting from row 2
# This loop locates the start and end point for each Verified alarm and labels *# Also, this loop locates the start and end point for each CANARY alarm and lab*
for (i in 2:nrow(dat)){
# Checks if the value in Verified_OW_Event_Alarm of the previous row (i-1) is *# the alarm has commenced and 1 is placed in Ver_Alarm_Start which marks the* if ((dat$Verified_OW_Event_Alarm[i-1]==0) & (dat$Verified_OW_Event_Alarm[i]==
{dat$Ver_Alarm_Start[i]<-1}

# Checks if the value in Verified_OW_Event_Alarm of the previous row (i-1) is *# the alarm has finished and 1 is placed in Ver_Alarm_End which marks the end* if ((dat$Verified_OW_Event_Alarm[i-1]==1) & (dat$Verified_OW_Event_Alarm[i]==
{dat$Ver_Alarm_End[i-1]<-1}

```
    # As above for P_Event
    #if ((dat$P_Event[i-1]==0.00000) & (dat$P_Event[i]==1.000000))
#{dat$CANARYTurb_Alarm_Start[i]<-1}       if ((dat$P_Event[i-1]<1) &
(dat$P_Event[i]==1.000000))      {dat$CANARYTurb_Alarm_Start[i]<-1}           if ((dat$P_Event[i-
1]==1) & (dat$P_Event[i]<1))       {dat$CANARYTurb_Alarm_End[i-1]<-1}

  }



  # locate the indices for the start and end points of each CANARY Alarm
  CANARYTurb_Alarm_Start_Indices<-grep(1,dat$CANARYTurb_Alarm_Start)
CANARYTurb_Alarm_End_Indices<-grep(1,dat$CANARYTurb_Alarm_End)

  # locate the indices for the start and points of each Verified Alarm
  Ver_Alarm_Start_Indices<-grep(1,dat$Ver_Alarm_Start)
  Ver_Alarm_End_Indices<-grep(1,dat$Ver_Alarm_End)

  # ALL CASES     # This only runs if "CANARY" has found at least one alarm.
  if(length(CANARYTurb_Alarm_Start_Indices)>0){ # checks for any start indices in       for (m
in 1:length(Ver_Alarm_Start_Indices)){  # This loop iterates over each
Ver_Alarm_Chunk_Length<-(Ver_Alarm_End_Indices[m]-Ver_Alarm_Start_Indices[m
CANARY_TEST_Chunk1<-dat$P_Event[(Ver_Alarm_Start_Indices[m]):(Ver_Alarm_End

     CANARY_Alarm_ON_Chunk1_Length<-sum(CANARY_TEST_Chunk1)
     if (Ver_Alarm_Chunk_Length==CANARY_Alarm_ON_Chunk1_Length)       {          # Now
extend the CANARY test Chunk by 1 step above and below.
      # If FULL_MATCH, then sum(CANARY test chunk) will not change.
      CANARY_TEST_Chunk2<-dat$P_Event[(Ver_Alarm_Start_Indices[m]-1):(Ver_Alarm
CANARY_Alarm_ON_Chunk2_Length<-sum(CANARY_TEST_Chunk2)
      if (CANARY_Alarm_ON_Chunk1_Length==CANARY_Alarm_ON_Chunk2_Length)
{dat$FULL_MATCH[Ver_Alarm_End_Indices[m]]<-"TRUE"}                 else
{dat$PARTIAL_MATCH[Ver_Alarm_End_Indices[m]]<-"TRUE"}
     }
                  else if (CANARY_Alarm_ON_Chunk1_Length>=1){
dat$PARTIAL_MATCH[Ver_Alarm_End_Indices[m]]<-"TRUE"       }

    }
  }

  (count_FULL_MATCH <- length(which(dat$FULL_MATCH == "TRUE")))
  (count_PARTIAL_MATCH <- length(which(dat$PARTIAL_MATCH == "TRUE")))
  (count_TOTAL_MATCH<-count_FULL_MATCH+count_PARTIAL_MATCH)
     # Count the number of actual alarms.
```

```
   Num_Ver_Alarms<-sum(dat$Ver_Alarm_Start==1)
      # Count the number of CANARY alarms.

   Num_CANARY_Alarms<-sum(dat$CANARYTurb_Alarm_Start==1)

   # Calculate the % Agreement between CANARY and Ver Alarms
   Percent_CANARY_Agreement_to_TOTAL_Verified_Events<-
round((count_TOTAL_MATCH/Num        current_row_for_output<-list(files_list[j],
count_FULL_MATCH, count_PARTIAL_MAT
   Algorithm_Scores[nrow(Algorithm_Scores) + 1,] <- current_row_for_output

   # Ensure that a folder is available to receive the output data/     # "data_set_identifer" will be
used as the folder name.        if (!dir.exists(paste0('09 ID CANARY Algorithm for turbidity
events/Output_File
      dat_out_directory<-paste0('09 ID CANARY Algorithm for turbidity events/Output_F
# Save Current dat to file, for troubleshoot or testing as required.
   if (!dir.exists(dat_out_directory)) dir.create(dat_out_directory, recursive = F
   write.csv(dat,(paste0(dat_out_directory,'/Dat_For_',files_list[j])), row.names=     if (j==1){     #
Only print this message once per processed directory.
   cat("Writing data to file:\n",(paste0(dat_out_directory,'/Dat_For_',files_list[

  }
}
## Writing data to file:
##  09 ID CANARY Algorithm for turbidity events/Output_Files/Data1/Dat_Files/Dat_Fo ##
Writing data to file: ##  09 ID CANARY Algorithm for turbidity
events/Output_Files/Data1/Dat_Files/Dat_Fo ## Writing data to file: ##  09 ID CANARY
Algorithm for turbidity events/Output_Files/Data1/Dat_Files/Dat_Fo ## Writing data to file:
##  09 ID CANARY Algorithm for turbidity events/Output_Files/Data1/Dat_Files/Dat_Fo ##
Writing data to file:
##  09 ID CANARY Algorithm for turbidity events/Output_Files/Data1/Dat_Files/Dat_Fo ##
Writing data to file: ##  09 ID CANARY Algorithm for turbidity
events/Output_Files/Data1/Dat_Files/Dat_Fo
```

```
dat$TIME_STEP<-format(dat$TIME_STEP) # this prevents 00:00:00 being removed from Ti
Location_and_Date_Range_ID_String<-paste0(sub("\\_L.*","",files_list[1]),"_")
Filename_and_path_for_algorithm_scores<-paste0("09 ID CANARY Algorithm for turbidit
write.csv(Algorithm_Scores,Filename_and_path_for_algorithm_scores, row.names=FALSE)
```

9.3 Output files for comparing the CANARY files to the manually identified events and identify the most suitable CANARY algorithm.

The output for step 9 identifying the best CANARY algorithm for turbidity events is saved in 09 ID CANARY Algorithm for turbidity events/Output_Files. It consists of two outputs, a Dat_Files directory containing 96 files and an 'Algo_scores files.

9.3.1 The Dat_Files directory

The Dat_Files directory contains each of the CANARY alorithm output files matched to the manually verified events file. In addition, for the sample data provided with this workflow, an example of how matching could be verified by the user is included in the file 'Match_highlighted_for_Dat_For_OW061024_051124C_LPCF_BED6_ET0.89063alg_1' in the supplementary material folder.

```
head(dat)
##           TIME_STEP P_Event Turbidity Verified_OW_Event_Alarm Ver_Alarm_Start
## 1 0                   0       0.0                 0             0
## 2 2024-10-06 13:45:00       0       3.2                 0             0
## 3 2024-10-06 14:00:00       0       3.2                 0             0
## 4 2024-10-06 14:15:00       0       3.2                 0             0
## 5 2024-10-06 14:30:00       0       3.2                 0             0
## 6 2024-10-06 14:45:00       0       2.8                 0             0
##   Ver_Alarm_End CANARYTurb_Alarm_Start CANARYTurb_Alarm_End FULL_MATCH
## 1           0                  0                  0        999
## 2           0                  0                  0        999
## 3           0                  0                  0        999
## 4           0                  0                  0        999
## 5           0                  0                  0        999
## 6           0                  0                  0        999
##   PARTIAL_MATCH
## 1           999
## 2           999
## 3           999
## 4           999
## 5           999
## 6           999
```

9.3.2 Algo scores file

The algo scores file summarises the number of full and partial matches for each CANARY algorithm and assigns a percent agreement to the total number of verified events. This can be used to identify the most suitable algorithm for the particular monitoring station based on the data provided to 'train' CANARY. It should be noted that adequate data covering a range of events over a time period is required to train CANARY and identify the most suitable algorithm. The optimum time period will depend on the frequency of events and the variability in event profiles for each monitoring station.

For this short dataset all algorithms show 100% identification of events i.e. they all identified the 4 manually identified events so more data would be required to distinguish the most suitable algorithm for this monitoring location.

```
head(Algorithm_Scores)
##                                  algorithm FULL_MATCH PARTIAL_MATCH
```

```
## 1  OW061024_051124C_LPCF_BED10_ET0.98926alg_1.csv         0           4
## 2 OW061024_051124C_LPCF_BED10_ET0.98926alg_10.csv         0           4
## 3 OW061024_051124C_LPCF_BED10_ET0.98926alg_11.csv         0           4
## 4 OW061024_051124C_LPCF_BED10_ET0.98926alg_12.csv         0           4
## 5 OW061024_051124C_LPCF_BED10_ET0.98926alg_13.csv         0           4
## 6 OW061024_051124C_LPCF_BED10_ET0.98926alg_14.csv         0           4
##   TOTAL_MATCH Total_CANARY_Events Total_Verified_Events
## 1           4                 112                     4
## 2           4                  38                     4
## 3           4                  36                     4
## 4           4                  26                     4
## 5           4                  34                     4
## 6           4                  15                     4
##   Percent_CANARY_Agreement_to_TOTAL_Verified_Events
## 1                                               100
## 2                                               100
## 3                                               100
## 4                                               100
## 5                                               100
## 6                                               100
```

References

Auguie, B. and Antonov, A. (2017) 'gridExtra: Miscellaneous Functions for "Grid" Graphics'. Available at: https://cran.r-project.org/web/packages/gridExtra/index.html (https://cran.rproject.org/web/packages/gridExtra/index.html) (Accessed: 28 April 2025).

Barrett, T. *et al.* (2024) 'Data.table: Extension of 'data.frame''. Available at: https://cran.rproject.org/web/packages/data.table/index.html (https://cran.rproject.org/web/packages/data.table/index.html) (Accessed: 14 February 2025).

Burkhardt, J.B. *et al.* (2022) 'Near real-time event detection for watershed monitoring with CANARY', *Environmental Science: Advances*, 1(2), pp. 170–181. Available at: https://doi.org/10.1039/D2VA00014H (https://doi.org/10.1039/D2VA00014H).

Edward, [https://stackoverflow.com/users/7514527/edward] (2024) 'Highlight areas in a time series when y is greater than a threshold value and a range of values'. Stackoverflow. Available at: https://stackoverflow.com/questions/78469527/highlight-area-in-a-time-series-when-y-is-greaterthan-a-threshold-value-and-a-r (https://stackoverflow.com/questions/78469527/highlight-area-in-atime-series-when-y-is-greater-than-a-threshold-value-and-a-r).

Hlavac, M. (2022) 'Stargazer: Well-Formatted Regression and Summary Statistics Tables'. Available at:

https://cran.r-project.org/web/packages/stargazer/index.html
(https://cran.rproject.org/web/packages/stargazer/index.html) (Accessed: 28 April 2025).
Kat, [https://stackoverflow.com/users/5329073/kat] (2024) 'Highlight areas of interactive time series plotly plot where y is greater than defined threshold and annotate them'. Stackoverflow. Available at: https://stackoverflow.com/questions/78761758/highlight-areas-of-interactive-time-series-plotly-plotwhere-y-is-greater-than-d (https://stackoverflow.com/questions/78761758/highlight-areas-ofinteractive-time-series-plotly-plot-where-y-is-greater-than-d).
Larmarange, J. *et al.* (2025) 'Labelled: Manipulating Labelled Data'. Available at: https://cran.rproject.org/web/packages/labelled/index.html (https://cran.rproject.org/web/packages/labelled/index.html) (Accessed: 16 February 2025).
Müller, K. and Bryan, J. (2020) 'Here: A Simpler Way to Find Your Files'. Available at: https://cran.rproject.org/web/packages/here/index.html (https://cran.rproject.org/web/packages/here/index.html) (Accessed: 14 February 2025).
Sievert, C. *et al.* (2024) 'Plotly: Create Interactive Web Graphics via 'plotly.js''. Available at: https://cran.rproject.org/web/packages/plotly/index.html (https://cran.rproject.org/web/packages/plotly/index.html) (Accessed: 14 February 2025).
Spinu, V. *et al.* (2024) 'Lubridate: Make Dealing with Dates a Little Easier'. Available at: https://cran.rproject.org/web/packages/lubridate/index.html (https://cran.rproject.org/web/packages/lubridate/index.html) (Accessed: 14 February 2025).
Team, R.C. (2024) 'R: A Language and Environment for Statistical Computing'. Vienna, Austria: R Foundation for Statistical Computing. Available at: https://www.R-project.org/ (https://www.Rproject.org/).
Tierney, N. *et al.* (2024) 'Naniar: Data Structures, Summaries, and Visualisations for Missing Data'. Available at: https://cran.r-project.org/web/packages/naniar/index.html (https://cran.rproject.org/web/packages/naniar/index.html) (Accessed: 28 April 2025).
Wickham, H., François, R., *et al.* (2023) 'Dplyr: A Grammar of Data Manipulation'. Available at: https://cran.r-project.org/web/packages/dplyr/index.html (https://cran.rproject.org/web/packages/dplyr/index.html) (Accessed: 14 February 2025).
Wickham, H., Pedersen, T.L., *et al.* (2023) 'Scales: Scale Functions for Visualization'. Available at:
https://cran.r-project.org/web/packages/scales/index.html (https://cran.rproject.org/web/packages/scales/index.html) (Accessed: 14 February 2025).
Wickham, H., Chang, W., *et al.* (2024) 'ggplot2: Create Elegant Data Visualisations Using the Grammar of Graphics'. Available at: https://cran.r-project.org/web/packages/ggplot2/index.html (https://cran.rproject.org/web/packages/ggplot2/index.html) (Accessed: 14 February 2025).
Wickham, H., Vaughan, D., *et al.* (2024) 'Tidyr: Tidy Messy Data'. Available at: https://cran.rproject.org/web/packages/tidyr/index.html (https://cran.rproject.org/web/packages/tidyr/index.html) (Accessed: 14 February 2025).
Wickham, H. and RStudio (2023) 'Tidyverse: Easily Install and Load the 'Tidyverse''. Available at:

https://cran.r-project.org/web/packages/tidyverse/index.html
(https://cran.rproject.org/web/packages/tidyverse/index.html) (Accessed: 14 February 2025).
Wickham, H., Software, P. and PBC (2023) 'Stringr: Simple, Consistent Wrappers for Common String Operations'. Available at: https://cran.r-project.org/web/packages/stringr/index.html (https://cran.rproject.org/web/packages/stringr/index.html) (Accessed: 28 April 2025).
Wilke, C.O. (2024) 'Cowplot: Streamlined Plot Theme and Plot Annotations for 'ggplot2''. Available at:
https://cran.r-project.org/web/packages/cowplot/index.html
(https://cran.rproject.org/web/packages/cowplot/index.html) (Accessed: 28 April 2025).
