# Supplementary material for: An open-source workflow for identifying hydrodynamic water quality events in rivers by continuous water quality monitoring and time-series data processing using R and US EPA CANARY
Source: MethodsX. 2025 Jul 27;15:103538. doi: 10.1016/j.mex.2025.103538 (PMC12347471; doi:10.1016/j.mex.2025.103538)
Supplement: Supplementary file 1 — Supplementary material and/or additional information The supplementary material for this research includes a detailed step by step user guide to the workflow for importing the data, running the R code, and for the output of the analysis provided as Supplementary Files S1-S4. S1: User Guide - Timeseries Water Quality data processing with R and CANARY for Hydrodynamic Event Monitoring. S2: Details for CANARY Output Files S3: Logic for identifying full and partial matches between manually verified events and CANARY events. S4: Example of match logic applied to _Dat_For_OW061024_051124C_LPCF_BED6_ET0.89063alg_1. S5: Supplementary References for non-peer reviewed publications. The source code used for visualisation and data analysis is publicly available on GitHub (Cronin and Taylor, 2025) along with a README file describing how to reproduce the results. The repository contains data, an interactive R markdown file with code and results of analysis, so users can apply the workflow directly to their own datasets. The repository is structured as per the nine-step protocol for data visualization and training of the CANARY event detection system detailed in Fig. 5 and detailed in the user guide. All materials are freely accessible under the MIT license. [file S2 Details for CANARY Output Files.pdf › S2 Details for CANARY Output Files.pdf]

Details for CANARY Output File

TIME_STEP	IDX	P_Event	Status	Clust_ID-1	Clust_Prob-1	Clust_ID-2	Clust_Prob-2	Clust_ID-3	Clust_Prob-3	Contrib_{Temp}	Contrib_{Specific_Conductance}	Contrib_{Turbidity}	Resid_{Temp}	Resid_{Specific_Conductance}	Resid_{Turbidity}	Comments
Date & Time	Index position	P_Event is the probability of an event for that algorithm and ranges from 0-1	0 is base behavior, no alarm,	Clustering Algorithm - rarely used and not used in this analysis so 'nan' values in this data set						Contrib_{XX} shows the fact that something is contributing to an alarm			For statuses of -1, 0, or 1, Resid will calculate the residual difference between the predicted behavior and measured value. This is then compared to the "outlier threshold".			
			, -1 is a reset signal related to the event timeout period							0, does not contribute						
			1 is actively alarming, which means P_event exceeds the "event threshold							1 or -1 is the direction of contribution associated with the Resid_{XX} value.			Outlier threshold - this parameter specifies the number of standard deviations of predicted error that must be met or exceeded before an outlier is declared (section 2.4 of user manual)			
			Status 3 - when all signals drop to zero the station is considered offline, the data is not analysed and these values are not shown in the plot<br><br>Status 3 - when all signals drop to zero the station is considered offline, the data is not analysed and these values are not shown in the plot													

References

Haxton, T. *et al*. (2013) 'Canary Training Tutorials'. USEPA/NHSRC. Available at: https://cfpub.epa.gov/si/si_public_file_download.cfm?p_download_id=516747.

Burkhardt, J. (2024) Email to Lisa Cronin, 6th December.
