# Supplementary material for: An open-source workflow for identifying hydrodynamic water quality events in rivers by continuous water quality monitoring and time-series data processing using R and US EPA CANARY
Source: MethodsX. 2025 Jul 27;15:103538. doi: 10.1016/j.mex.2025.103538 (PMC12347471; doi:10.1016/j.mex.2025.103538)
Supplement: Supplementary file 1 — Supplementary material and/or additional information The supplementary material for this research includes a detailed step by step user guide to the workflow for importing the data, running the R code, and for the output of the analysis provided as Supplementary Files S1-S4. S1: User Guide - Timeseries Water Quality data processing with R and CANARY for Hydrodynamic Event Monitoring. S2: Details for CANARY Output Files S3: Logic for identifying full and partial matches between manually verified events and CANARY events. S4: Example of match logic applied to _Dat_For_OW061024_051124C_LPCF_BED6_ET0.89063alg_1. S5: Supplementary References for non-peer reviewed publications. The source code used for visualisation and data analysis is publicly available on GitHub (Cronin and Taylor, 2025) along with a README file describing how to reproduce the results. The repository contains data, an interactive R markdown file with code and results of analysis, so users can apply the workflow directly to their own datasets. The repository is structured as per the nine-step protocol for data visualization and training of the CANARY event detection system detailed in Fig. 5 and detailed in the user guide. All materials are freely accessible under the MIT license. [file S3 Logic for identifying full & partial matches.pdf › S3 Logic for identifying full & partial matches.pdf]

Workflow for Identifying Water Quality Hydrodynamic Events in Time Series Data

Logic for identifying full match and partial match between manually verified events (identified by visual screening of the turbidity data) and events identified by EPA CANARY event detection software.

Take the following scenario:

- Alarm A: 13:00 – 17:00
    - Event start timestep – 13:00
    - Event end timestep – 17:00

- Alarm B: 15:00 – 22:00
    - Event start timestep – 15:00
    - Event end timestep – 22:00

Full Match

For a Full Match the timestep when the event starts and the timestep when the event ends must match in both the manually identified file and in the CANARY output file.

Partial Match

For a partial match, there mut be an overlap in either the start time or the end time of an event.

If Alarm B is from a "true" case i.e. is manually verified via visual inspection of the water quality trend, it is deemed a partial match if:

Case 1: A is completely in B

Case 2: B is completely in A

Case 3: start of A is within the start and end time of B

Case 4: end of A is within the start and end time of B

(Burkhardt, 2024)

Burkhardt, J. (2024) 'Event Matching for Near real time event detection for watershed monitoring with CANARY'. Email correspondence between Jonathan Burkhardt and Lisa Cronin.

Example of the Logic applied to dataset

(Step 8 of the workflow)

1. Code locates the start and end index at which each verified alarm occurs. In the example shown below, the *start index* is 3 and the *end index* is 9.

TIME_STEP	CANARY_Alarm	Verified_Alarm	Index
26/11/2022 00:00	0	0	1
26/11/2022 00:15	0	0	2
26/11/2022 00:30	1	1	**3**
26/11/2022 00:45	1	1	4
26/11/2022 01:00	1	1	5
26/11/2022 01:15	1	1	6
26/11/2022 01:30	1	1	7
26/11/2022 01:45	1	1	8
26/11/2022 02:00	1	1	**9**
26/11/2022 02:15	0	0	10
26/11/2022 02:30	0	0	11
26/11/2022 02:45	0	0	12
26/11/2022 03:00	0	0	13

2. The length of the Verified_Alarm is calculated as $(end\ index - start\ index) + 1$. In this example Verified_Alarm length is 7.
3. Code then looks at the "CANARY_Alarm for those same indices and creates a "test chunk" between them. The test chunk is shaded in orange in the example below. This test chunk is called "CANARY Chunk 1".

TIME_STEP	CANARY_Alarm	Verified_Alarm	Index
26/11/2022 00:00	0	0	1
26/11/2022 00:15	0	0	2
26/11/2022 00:30	1	1	**3**
26/11/2022 00:45	1	1	4
26/11/2022 01:00	1	1	5
26/11/2022 01:15	1	1	6
26/11/2022 01:30	1	1	7
26/11/2022 01:45	1	1	8
26/11/2022 02:00	1	1	**9**
26/11/2022 02:15	0	0	10
26/11/2022 02:30	0	0	11
26/11/2022 02:45	0	0	12
26/11/2022 03:00	0	0	13

4. The values in the test chunk are all added together.
5. If the test chunk values sum to '0'. This is a MISSED ALARM.
6. If the test chunk values sum to less than 7, this is a PARTIAL MATCH.
7. If the test chunk values sum is the same as Verified_Alarm length, this *may* indicate a FULL MATCH. In this example the values sum to give a value of 7 which is the same as the length of the Verified_Alarm.
8. So, the "test chunk" and Verified_Alarm, *may* be the same length.
9. One other possibility is that the CANARY test chunk is wider than the Verified Alarm. To see if this is the case, a 2nd test chunk is created by extending the original test chunk one step earlier and later. In this example, the 2nd test chunk is shown in blue below.

TIME_STEP	CANARY_Alarm	Verified_Alarm	Index
26/11/2022 00:00	0	0	1
26/11/2022 00:15	0	0	2
26/11/2022 00:30	1	1	**3**
26/11/2022 00:45	1	1	4
26/11/2022 01:00	1	1	5
26/11/2022 01:15	1	1	6
26/11/2022 01:30	1	1	7
26/11/2022 01:45	1	1	8
26/11/2022 02:00	1	1	**9**
26/11/2022 02:15	0	0	10
26/11/2022 02:30	0	0	11
26/11/2022 02:45	0	0	12
26/11/2022 03:00	0	0	13

10. Now the values in "test chunk" 2 are all added together. If the sum doesn't change (still 7 in this example), then the extra time steps which were incorporated both contain "0s". In other words the CANARY_Alarm and Verified_Alarm are a "FULL MATCH".
11. If the sum in "test chunk 2" does change (to 8 or 9 in this example), that indicates the CANARY_Alarm is wider than the verified alarm. This is a PARTIAL MATCH.
12. The code loops over every Verified alarm in the manner described above.
