# Supplementary material for: An open-source workflow for identifying hydrodynamic water quality events in rivers by continuous water quality monitoring and time-series data processing using R and US EPA CANARY
Source: MethodsX. 2025 Jul 27;15:103538. doi: 10.1016/j.mex.2025.103538 (PMC12347471; doi:10.1016/j.mex.2025.103538)
Supplement: Supplementary file 1 — Supplementary material and/or additional information The supplementary material for this research includes a detailed step by step user guide to the workflow for importing the data, running the R code, and for the output of the analysis provided as Supplementary Files S1-S4. S1: User Guide - Timeseries Water Quality data processing with R and CANARY for Hydrodynamic Event Monitoring. S2: Details for CANARY Output Files S3: Logic for identifying full and partial matches between manually verified events and CANARY events. S4: Example of match logic applied to _Dat_For_OW061024_051124C_LPCF_BED6_ET0.89063alg_1. S5: Supplementary References for non-peer reviewed publications. The source code used for visualisation and data analysis is publicly available on GitHub (Cronin and Taylor, 2025) along with a README file describing how to reproduce the results. The repository contains data, an interactive R markdown file with code and results of analysis, so users can apply the workflow directly to their own datasets. The repository is structured as per the nine-step protocol for data visualization and training of the CANARY event detection system detailed in Fig. 5 and detailed in the user guide. All materials are freely accessible under the MIT license. [file S4 Match logic applied to_Dat_ET0.89063alg_1.pdf › S4 Match logic applied to_Dat_ET0.89063alg_1.pdf]

TIME_STEP	P_Event	Turbidity	Verified_OW_Event_Alarm	Ver_Alarm_Start	Ver_Alarm_End	CANARYTurb_Alarm_Start	CANARYTurb_Alarm_End	FULL_MATCH	PARTIAL_MATCH
0	0	0	0	0	0	0	0	999	999
06/10/2024 13:45	0	3.2	0	0	0	0	0	999	999
06/10/2024 14:00	0	3.2	0	0	0	0	0	999	999
06/10/2024 14:15	0	3.2	0	0	0	0	0	999	999
06/10/2024 14:30	0	3.2	0	0	0	0	0	999	999
06/10/2024 14:45	0	2.8	0	0	0	0	0	999	999
06/10/2024 15:00	0	2.9	0	0	0	0	0	999	999
06/10/2024 15:15	0	3.2	0	0	0	0	0	999	999
06/10/2024 15:30	0	3.3	0	0	0	0	0	999	999
06/10/2024 15:45	0	3.5	0	0	0	0	0	999	999
06/10/2024 16:00	0	3.2	0	0	0	0	0	999	999
06/10/2024 16:15	0	3.2	0	0	0	0	0	999	999
06/10/2024 16:30	0	3.1	0	0	0	0	0	999	999
06/10/2024 16:45	0	3.5	0	0	0	0	0	999	999
06/10/2024 17:00	0	3.4	0	0	0	0	0	999	999
06/10/2024 17:15	0	2.9	0	0	0	0	0	999	999
06/10/2024 17:30	0	3	0	0	0	0	0	999	999
06/10/2024 17:45	0	2.9	0	0	0	0	0	999	999
06/10/2024 18:00	0	3.1	0	0	0	0	0	999	999
06/10/2024 18:15	0	3.1	0	0	0	0	0	999	999
06/10/2024 18:30	0	2.7	0	0	0	0	0	999	999
06/10/2024 18:45	0	3.1	0	0	0	0	0	999	999
06/10/2024 19:00	0	2.8	0	0	0	0	0	999	999
06/10/2024 19:15	0	2.9	0	0	0	0	0	999	999
06/10/2024 19:30	0	2.5	0	0	0	0	0	999	999
06/10/2024 19:45	0.003906	2.8	0	0	0	0	0	999	999
06/10/2024 20:00	0.035156	2.6	0	0	0	0	0	999	999
06/10/2024 20:15	0.144531	2.8	0	0	0	0	0	999	999
06/10/2024 20:30	0.363281	2.8	0	0	0	0	0	999	999
06/10/2024 20:45	0.636719	2.8	0	0	0	0	0	999	999
06/10/2024 21:00	0.855469	2.7	0	0	0	0	0	999	999
06/10/2024 21:15	0.964844	3	0	0	0	0	0	999	999
06/10/2024 21:30	0.996094	2.7	0	0	0	0	0	999	999
06/10/2024 21:45	1	2.7	0	0	0	1	0	999	999
06/10/2024 22:00	1	2.6	0	0	0	0	0	999	999

06/10/2024 22:15	1	2.6	0	0	0	0	0	999	999
06/10/2024 22:30	1	2.6	0	0	0	0	0	999	999
06/10/2024 22:45	1	2.3	0	0	0	0	0	999	999
06/10/2024 23:00	1	2.7	0	0	0	0	0	999	999
06/10/2024 23:15	1	2.7	0	0	0	0	0	999	999
06/10/2024 23:30	1	2.5	0	0	0	0	0	999	999
06/10/2024 23:45	1	2.6	0	0	0	0	0	999	999
07/10/2024 00:00	1	2.5	0	0	0	0	1	999	999
07/10/2024 00:15	0.035156	4.3	0	0	0	0	0	999	999
07/10/2024 00:30	0.035156	2.6	0	0	0	0	0	999	999
07/10/2024 00:45	0.035156	2.6	0	0	0	0	0	999	999
07/10/2024 01:00	0.035156	2.5	0	0	0	0	0	999	999
07/10/2024 01:15	0.035156	2.7	0	0	0	0	0	999	999
07/10/2024 01:30	0.035156	2.6	0	0	0	0	0	999	999
07/10/2024 01:45	0.035156	2.6	0	0	0	0	0	999	999
07/10/2024 02:00	0.035156	2.5	0	0	0	0	0	999	999
07/10/2024 02:15	0.035156	2.6	0	0	0	0	0	999	999
07/10/2024 02:30	0.144531	2.6	0	0	0	0	0	999	999
07/10/2024 02:45	0.363281	2.6	0	0	0	0	0	999	999
07/10/2024 03:00	0.636719	2.7	0	0	0	0	0	999	999
07/10/2024 03:15	0.855469	2.5	0	0	0	0	0	999	999
07/10/2024 03:30	0.964844	2.8	0	0	0	0	0	999	999
07/10/2024 03:45	0.996094	2.6	0	0	0	0	0	999	999
07/10/2024 04:00	1	2.8	0	0	0	1	0	999	999
07/10/2024 04:15	1	2.6	0	0	0	0	0	999	999
07/10/2024 04:30	1	2.6	0	0	0	0	0	999	999
07/10/2024 04:45	1	2.8	0	0	0	0	0	999	999
07/10/2024 05:00	1	2.8	0	0	0	0	0	999	999
07/10/2024 05:15	1	2.7	0	0	0	0	0	999	999
07/10/2024 05:30	1	2.8	0	0	0	0	0	999	999
07/10/2024 05:45	1	2.8	0	0	0	0	0	999	999
07/10/2024 06:00	1	2.8	0	0	0	0	0	999	999
07/10/2024 06:15	1	2.9	0	0	0	0	1	999	999
07/10/2024 06:30	0.035156	3.5	0	0	0	0	0	999	999
07/10/2024 06:45	0.035156	2.9	0	0	0	0	0	999	999
07/10/2024 07:00	0.035156	2.9	0	0	0	0	0	999	999

07/10/2024 07:15	0.035156	2.9	0	0	0	0	0	999	999
07/10/2024 07:30	0.035156	3.1	0	0	0	0	0	999	999
07/10/2024 07:45	0.035156	2.8	0	0	0	0	0	999	999
07/10/2024 08:00	0.035156	3	0	0	0	0	0	999	999
07/10/2024 08:15	0.144531	3.1	0	0	0	0	0	999	999
07/10/2024 08:30	0.035156	2.9	0	0	0	0	0	999	999
07/10/2024 08:45	0.035156	2.9	0	0	0	0	0	999	999
07/10/2024 09:00	0.035156	3	0	0	0	0	0	999	999
07/10/2024 09:15	0.035156	2.9	0	0	0	0	0	999	999
07/10/2024 09:30	0.144531	3.1	0	0	0	0	0	999	999
07/10/2024 09:45	0.363281	3.4	0	0	0	0	0	999	999
07/10/2024 10:00	0.636719	3.1	0	0	0	0	0	999	999
07/10/2024 10:15	0.636719	3	0	0	0	0	0	999	999
07/10/2024 10:30	0.855469	3.1	0	0	0	0	0	999	999
07/10/2024 10:45	0.964844	3.8	0	0	0	0	0	999	999
07/10/2024 11:00	0.996094	3.2	0	0	0	0	0	999	999
07/10/2024 11:15	1	3.1	0	0	0	1	0	999	999
07/10/2024 11:30	1	3.5	0	0	0	0	0	999	999
07/10/2024 11:45	1	3.2	0	0	0	0	0	999	999
07/10/2024 12:00	1	3.1	0	0	0	0	0	999	999
07/10/2024 12:15	1	3.8	0	0	0	0	0	999	999
07/10/2024 12:30	1	3.2	0	0	0	0	0	999	999
07/10/2024 12:45	1	3.2	0	0	0	0	0	999	999
07/10/2024 13:00	1	3.1	0	0	0	0	0	999	999
07/10/2024 13:15	1	2.9	0	0	0	0	0	999	999
07/10/2024 13:30	1	3.1	0	0	0	0	1	999	999
07/10/2024 13:45	0.035156	3.7	0	0	0	0	0	999	999
07/10/2024 14:00	0.144531	3.4	0	0	0	0	0	999	999
07/10/2024 14:15	0.363281	3	0	0	0	0	0	999	999
07/10/2024 14:30	0.636719	3.2	0	0	0	0	0	999	999
07/10/2024 14:45	0.855469	3.6	0	0	0	0	0	999	999
07/10/2024 15:00	0.964844	2.9	0	0	0	0	0	999	999
07/10/2024 15:15	0.996094	3	0	0	0	0	0	999	999
07/10/2024 15:30	1	3	0	0	0	1	0	999	999
07/10/2024 15:45	1	2.9	0	0	0	0	0	999	999
07/10/2024 16:00	1	3	0	0	0	0	0	999	999

07/10/2024 16:15	1	3	0	0	0	0	0	999	999
07/10/2024 16:30	1	2.9	0	0	0	0	0	999	999
07/10/2024 16:45	1	3.1	0	0	0	0	0	999	999
07/10/2024 17:00	1	3	0	0	0	0	0	999	999
07/10/2024 17:15	1	3.1	0	0	0	0	0	999	999
07/10/2024 17:30	1	3	0	0	0	0	0	999	999
07/10/2024 17:45	1	2.8	0	0	0	0	1	999	999
07/10/2024 18:00	0.003906	2.8	0	0	0	0	0	999	999
07/10/2024 18:15	0.003906	2.8	0	0	0	0	0	999	999
07/10/2024 18:30	0.003906	2.8	0	0	0	0	0	999	999
07/10/2024 18:45	0.003906	2.8	0	0	0	0	0	999	999
07/10/2024 19:00	0.003906	2.8	0	0	0	0	0	999	999
07/10/2024 19:15	0.035156	3.3	0	0	0	0	0	999	999
07/10/2024 19:30	0.144531	3.4	0	0	0	0	0	999	999
07/10/2024 19:45	0.363281	3.2	0	0	0	0	0	999	999
07/10/2024 20:00	0.363281	3.1	0	0	0	0	0	999	999
07/10/2024 20:15	0.363281	3.3	0	0	0	0	0	999	999
07/10/2024 20:30	0.363281	2.9	0	0	0	0	0	999	999
07/10/2024 20:45	0.636719	2.8	0	0	0	0	0	999	999
07/10/2024 21:00	0.636719	3.3	0	0	0	0	0	999	999
07/10/2024 21:15	0.363281	2.9	0	0	0	0	0	999	999
07/10/2024 21:30	0.363281	2.7	0	0	0	0	0	999	999
07/10/2024 21:45	0.363281	2.8	0	0	0	0	0	999	999
07/10/2024 22:00	0.636719	2.5	0	0	0	0	0	999	999
07/10/2024 22:15	0.855469	2.8	0	0	0	0	0	999	999
07/10/2024 22:30	0.964844	2.8	0	0	0	0	0	999	999
07/10/2024 22:45	0.964844	2.8	0	0	0	0	0	999	999
07/10/2024 23:00	0.996094	2.7	0	0	0	0	0	999	999
07/10/2024 23:15	1	150.5	1	1	0	1	0	999	999
07/10/2024 23:30	1	8.8	1	0	0	0	0	999	999
07/10/2024 23:45	1	5.2	1	0	0	0	0	999	999
08/10/2024 00:00	1	105.5	1	0	0	0	0	999	999
08/10/2024 00:15	1	89.2	1	0	0	0	0	999	999
08/10/2024 00:30	1	20	1	0	0	0	0	999	999
08/10/2024 00:45	1	11.2	1	0	0	0	0	999	999
08/10/2024 01:00	1	4.9	1	0	0	0	0	999	999

08/10/2024 01:15	1	542.6	1	0	0	0	1	999	999
08/10/2024 01:30	0.003906	6.8	1	0	0	0	0	999	999
08/10/2024 01:45	0.035156	5.6	1	0	0	0	0	999	999
08/10/2024 02:00	0.144531	5.4	1	0	0	0	0	999	999
08/10/2024 02:15	0.363281	79.8	1	0	0	0	0	999	999
08/10/2024 02:30	0.636719	6	1	0	0	0	0	999	999
08/10/2024 02:45	0.855469	110.1	1	0	1	0	0	999	TRUE
08/10/2024 03:00	0.964844	2.8	0	0	0	0	0	999	999
08/10/2024 03:15	0.996094	2.9	0	0	0	0	0	999	999
08/10/2024 03:30	1	2.8	0	0	0	1	0	999	999
08/10/2024 03:45	1	2.9	0	0	0	0	0	999	999
08/10/2024 04:00	1	2.6	0	0	0	0	0	999	999
08/10/2024 04:15	1	2.9	0	0	0	0	0	999	999
08/10/2024 04:30	1	2.8	0	0	0	0	0	999	999
08/10/2024 04:45	1	2.8	0	0	0	0	0	999	999
08/10/2024 05:00	1	2.8	0	0	0	0	0	999	999
08/10/2024 05:15	1	2.8	0	0	0	0	0	999	999
08/10/2024 05:30	1	2.9	0	0	0	0	0	999	999
08/10/2024 05:45	1	2.8	0	0	0	0	1	999	999
08/10/2024 06:00	0.035156	2.8	0	0	0	0	0	999	999
08/10/2024 06:15	0.144531	2.9	0	0	0	0	0	999	999
08/10/2024 06:30	0.363281	2.9	0	0	0	0	0	999	999
08/10/2024 06:45	0.636719	2.9	0	0	0	0	0	999	999
08/10/2024 07:00	0.855469	3	0	0	0	0	0	999	999
08/10/2024 07:15	0.964844	2.9	0	0	0	0	0	999	999
08/10/2024 07:30	0.996094	3	0	0	0	0	0	999	999
08/10/2024 07:45	1	3	0	0	0	1	0	999	999
08/10/2024 08:00	1	2.8	0	0	0	0	0	999	999
08/10/2024 08:15	1	3.2	0	0	0	0	0	999	999
08/10/2024 08:30	1	2.9	0	0	0	0	0	999	999
08/10/2024 08:45	1	2.9	0	0	0	0	0	999	999
08/10/2024 09:00	1	3.1	0	0	0	0	0	999	999
08/10/2024 09:15	1	3.4	0	0	0	0	0	999	999
08/10/2024 09:30	1	3.4	0	0	0	0	0	999	999
08/10/2024 09:45	1	3.3	0	0	0	0	0	999	999
08/10/2024 10:00	1	3.3	0	0	0	0	1	999	999

08/10/2024 10:15	0.035156	3.6	0	0	0	0	0	999	999
08/10/2024 10:30	0.035156	3.4	0	0	0	0	0	999	999
08/10/2024 10:45	0.035156	3.2	0	0	0	0	0	999	999
08/10/2024 11:00	0.144531	2.7	0	0	0	0	0	999	999
08/10/2024 11:15	0.144531	3.3	0	0	0	0	0	999	999
08/10/2024 11:30	0.144531	3.2	0	0	0	0	0	999	999
08/10/2024 11:45	0.144531	2.9	0	0	0	0	0	999	999
08/10/2024 12:00	0.144531	2.9	0	0	0	0	0	999	999
08/10/2024 12:15	0.035156	3.1	0	0	0	0	0	999	999
08/10/2024 12:30	0.144531	2.9	0	0	0	0	0	999	999
08/10/2024 12:45	0.363281	2.8	0	0	0	0	0	999	999
08/10/2024 13:00	0.363281	3.1	0	0	0	0	0	999	999
08/10/2024 13:15	0.363281	2.6	0	0	0	0	0	999	999
08/10/2024 13:30	0.636719	2.8	0	0	0	0	0	999	999
08/10/2024 13:45	0.855469	2.9	0	0	0	0	0	999	999
08/10/2024 14:00	0.964844	2.9	0	0	0	0	0	999	999
08/10/2024 14:15	0.996094	2.9	0	0	0	0	0	999	999
08/10/2024 14:30	1	2.9	0	0	0	1	0	999	999
08/10/2024 14:45	1	2.8	0	0	0	0	0	999	999
08/10/2024 15:00	1	2.9	0	0	0	0	0	999	999
08/10/2024 15:15	1	4.2	0	0	0	0	0	999	999
08/10/2024 15:30	1	2.9	0	0	0	0	0	999	999
08/10/2024 15:45	1	2.8	0	0	0	0	0	999	999
08/10/2024 16:00	1	2.8	0	0	0	0	0	999	999
08/10/2024 16:15	1	3	0	0	0	0	0	999	999
08/10/2024 16:30	1	2.9	0	0	0	0	0	999	999
08/10/2024 16:45	1	2.9	0	0	0	0	1	999	999
08/10/2024 17:00	0.035156	2.7	0	0	0	0	0	999	999
08/10/2024 17:15	0.144531	2.8	0	0	0	0	0	999	999
08/10/2024 17:30	0.363281	2.8	0	0	0	0	0	999	999
08/10/2024 17:45	0.636719	3.4	0	0	0	0	0	999	999
08/10/2024 18:00	0.855469	2.9	0	0	0	0	0	999	999
08/10/2024 18:15	0.964844	2.7	0	0	0	0	0	999	999
08/10/2024 18:30	0.996094	2.9	0	0	0	0	0	999	999
08/10/2024 18:45	1	2.8	0	0	0	1	0	999	999
08/10/2024 19:00	1	2.8	0	0	0	0	0	999	999

08/10/2024 19:15	1	2.8	0	0	0	0	0	999	999
08/10/2024 19:30	1	2.9	0	0	0	0	0	999	999
08/10/2024 19:45	1	2.6	0	0	0	0	0	999	999
08/10/2024 20:00	1	2.8	0	0	0	0	0	999	999
08/10/2024 20:15	1	3.1	0	0	0	0	0	999	999
08/10/2024 20:30	1	2.7	0	0	0	0	0	999	999
08/10/2024 20:45	1	2.7	0	0	0	0	0	999	999
08/10/2024 21:00	1	2.9	0	0	0	0	1	999	999
08/10/2024 21:15	0.035156	2.7	0	0	0	0	0	999	999
08/10/2024 21:30	0.144531	2.8	0	0	0	0	0	999	999
08/10/2024 21:45	0.363281	2.4	0	0	0	0	0	999	999
08/10/2024 22:00	0.636719	2.7	0	0	0	0	0	999	999
08/10/2024 22:15	0.855469	2.7	0	0	0	0	0	999	999
08/10/2024 22:30	0.964844	2.8	0	0	0	0	0	999	999
08/10/2024 22:45	0.996094	2.9	0	0	0	0	0	999	999
08/10/2024 23:00	1	2.8	0	0	0	1	0	999	999
08/10/2024 23:15	1	2.9	0	0	0	0	0	999	999
08/10/2024 23:30	1	2.7	0	0	0	0	0	999	999
08/10/2024 23:45	1	2.9	0	0	0	0	0	999	999
09/10/2024 00:00	1	2.8	0	0	0	0	0	999	999
09/10/2024 00:15	1	2.8	0	0	0	0	0	999	999
09/10/2024 00:30	1	3	0	0	0	0	0	999	999
09/10/2024 00:45	1	2.9	0	0	0	0	0	999	999
09/10/2024 01:00	1	2.9	0	0	0	0	0	999	999
09/10/2024 01:15	1	3.2	0	0	0	0	1	999	999
09/10/2024 01:30	0.035156	2.9	0	0	0	0	0	999	999
09/10/2024 01:45	0.144531	3	0	0	0	0	0	999	999
09/10/2024 02:00	0.363281	3.2	0	0	0	0	0	999	999
09/10/2024 02:15	0.636719	3.2	0	0	0	0	0	999	999
09/10/2024 02:30	0.855469	3	0	0	0	0	0	999	999
09/10/2024 02:45	0.964844	3.4	0	0	0	0	0	999	999
09/10/2024 03:00	0.996094	3.1	0	0	0	0	0	999	999
09/10/2024 03:15	1	3.3	0	0	0	1	0	999	999
09/10/2024 03:30	1	3.3	0	0	0	0	0	999	999
09/10/2024 03:45	1	3.2	0	0	0	0	0	999	999
09/10/2024 04:00	1	3.4	0	0	0	0	0	999	999

09/10/2024 04:15	1	3.6	0	0	0	0	0	999	999
09/10/2024 04:30	1	3.4	0	0	0	0	0	999	999
09/10/2024 04:45	1	3.4	0	0	0	0	0	999	999
09/10/2024 05:00	1	3.4	0	0	0	0	0	999	999
09/10/2024 05:15	1	3.9	0	0	0	0	0	999	999
09/10/2024 05:30	1	3.7	0	0	0	0	1	999	999
09/10/2024 05:45	0.003906	3.9	0	0	0	0	0	999	999
09/10/2024 06:00	0.003906	3.4	0	0	0	0	0	999	999
09/10/2024 06:15	0.035156	3.5	0	0	0	0	0	999	999
09/10/2024 06:30	0.144531	3.6	0	0	0	0	0	999	999
09/10/2024 06:45	0.363281	3.7	0	0	0	0	0	999	999
09/10/2024 07:00	0.636719	3.3	0	0	0	0	0	999	999
09/10/2024 07:15	0.855469	3.6	0	0	0	0	0	999	999
09/10/2024 07:30	0.964844	3.6	0	0	0	0	0	999	999
09/10/2024 07:45	0.996094	3.3	0	0	0	0	0	999	999
09/10/2024 08:00	1	3.5	0	0	0	1	0	999	999
09/10/2024 08:15	1	4	0	0	0	0	0	999	999
09/10/2024 08:30	1	3.5	0	0	0	0	0	999	999
09/10/2024 08:45	1	3.7	0	0	0	0	0	999	999
09/10/2024 09:00	1	4.2	0	0	0	0	0	999	999
09/10/2024 09:15	1	4.3	0	0	0	0	0	999	999
09/10/2024 09:30	1	3.9	0	0	0	0	0	999	999
09/10/2024 09:45	1	3.6	0	0	0	0	0	999	999
09/10/2024 10:00	1	4.2	0	0	0	0	0	999	999
09/10/2024 10:15	1	3.5	0	0	0	0	1	999	999
09/10/2024 10:30	0.003906	3.7	0	0	0	0	0	999	999
09/10/2024 10:45	0.003906	3.6	0	0	0	0	0	999	999
09/10/2024 11:00	0.003906	4.3	0	0	0	0	0	999	999
09/10/2024 11:15	0.035156	3.6	0	0	0	0	0	999	999
09/10/2024 11:30	0.144531	3.9	0	0	0	0	0	999	999
09/10/2024 11:45	0.363281	3.5	0	0	0	0	0	999	999
09/10/2024 12:00	0.636719	3.6	0	0	0	0	0	999	999
09/10/2024 12:15	0.855469	3.6	0	0	0	0	0	999	999
09/10/2024 12:30	0.964844	3.4	0	0	0	0	0	999	999
09/10/2024 12:45	0.996094	4.7	0	0	0	0	0	999	999
09/10/2024 13:00	1	3.9	0	0	0	1	0	999	999

09/10/2024 13:15	1	3.6	0	0	0	0	0	999	999
09/10/2024 13:30	1	4	0	0	0	0	0	999	999
09/10/2024 13:45	1	3.2	0	0	0	0	0	999	999
09/10/2024 14:00	1	3.4	0	0	0	0	0	999	999
09/10/2024 14:15	1	3.2	0	0	0	0	0	999	999
09/10/2024 14:30	1	3.2	0	0	0	0	0	999	999
09/10/2024 14:45	1	3.2	0	0	0	0	0	999	999
09/10/2024 15:00	1	3.2	0	0	0	0	0	999	999
09/10/2024 15:15	1	3.5	0	0	0	0	1	999	999
09/10/2024 15:30	0.003906	3.3	0	0	0	0	0	999	999
09/10/2024 15:45	0.035156	2.9	0	0	0	0	0	999	999
09/10/2024 16:00	0.144531	3.4	0	0	0	0	0	999	999
09/10/2024 16:15	0.363281	3.2	0	0	0	0	0	999	999
09/10/2024 16:30	0.636719	3.1	0	0	0	0	0	999	999
09/10/2024 16:45	0.855469	3.2	0	0	0	0	0	999	999
09/10/2024 17:00	0.964844	3.5	0	0	0	0	0	999	999
09/10/2024 17:15	0.964844	3.4	0	0	0	0	0	999	999
09/10/2024 17:30	0.964844	3.4	0	0	0	0	0	999	999
09/10/2024 17:45	0.855469	3	0	0	0	0	0	999	999
09/10/2024 18:00	0.636719	3.2	0	0	0	0	0	999	999
09/10/2024 18:15	0.363281	3.1	0	0	0	0	0	999	999
09/10/2024 18:30	0.144531	3.4	0	0	0	0	0	999	999
09/10/2024 18:45	0.035156	3.1	0	0	0	0	0	999	999
09/10/2024 19:00	0.003906	3	0	0	0	0	0	999	999
09/10/2024 19:15	0.003906	3.1	0	0	0	0	0	999	999
09/10/2024 19:30	0.035156	3.2	0	0	0	0	0	999	999
09/10/2024 19:45	0.144531	3.4	0	0	0	0	0	999	999
09/10/2024 20:00	0.363281	3.2	0	0	0	0	0	999	999
09/10/2024 20:15	0.636719	3.1	0	0	0	0	0	999	999
09/10/2024 20:30	0.855469	3.1	0	0	0	0	0	999	999
09/10/2024 20:45	0.964844	3.2	0	0	0	0	0	999	999
09/10/2024 21:00	0.996094	3.4	0	0	0	0	0	999	999
09/10/2024 21:15	1	3.2	0	0	0	1	0	999	999
09/10/2024 21:30	1	3.5	0	0	0	0	0	999	999
09/10/2024 21:45	1	3.2	0	0	0	0	0	999	999
09/10/2024 22:00	1	3.3	0	0	0	0	0	999	999

09/10/2024 22:15	1	3.2	0	0	0	0	0	999	999
09/10/2024 22:30	1	3.4	0	0	0	0	0	999	999
09/10/2024 22:45	1	3.3	0	0	0	0	0	999	999
09/10/2024 23:00	1	3.3	0	0	0	0	0	999	999
09/10/2024 23:15	1	3.2	0	0	0	0	0	999	999
09/10/2024 23:30	1	3.2	0	0	0	0	1	999	999
09/10/2024 23:45	0.003906	3.5	0	0	0	0	0	999	999
10/10/2024 00:00	0.035156	3.4	0	0	0	0	0	999	999
10/10/2024 00:15	0.035156	3.5	0	0	0	0	0	999	999
10/10/2024 00:30	0.144531	3.4	0	0	0	0	0	999	999
10/10/2024 00:45	0.144531	3.5	0	0	0	0	0	999	999
10/10/2024 01:00	0.363281	3.2	0	0	0	0	0	999	999
10/10/2024 01:15	0.363281	3.3	0	0	0	0	0	999	999
10/10/2024 01:30	0.363281	3.2	0	0	0	0	0	999	999
10/10/2024 01:45	0.363281	3.5	0	0	0	0	0	999	999
10/10/2024 02:00	0.144531	3.4	0	0	0	0	0	999	999
10/10/2024 02:15	0.144531	3.4	0	0	0	0	0	999	999
10/10/2024 02:30	0.035156	9.3	0	0	0	0	0	999	999
10/10/2024 02:45	0.144531	3.4	0	0	0	0	0	999	999
10/10/2024 03:00	0.035156	3.8	0	0	0	0	0	999	999
10/10/2024 03:15	0.144531	3.6	0	0	0	0	0	999	999
10/10/2024 03:30	0.363281	3.7	0	0	0	0	0	999	999
10/10/2024 03:45	0.636719	3.6	0	0	0	0	0	999	999
10/10/2024 04:00	0.855469	3.4	0	0	0	0	0	999	999
10/10/2024 04:15	0.855469	3.7	0	0	0	0	0	999	999
10/10/2024 04:30	0.964844	3.6	0	0	0	0	0	999	999
10/10/2024 04:45	0.964844	3.4	0	0	0	0	0	999	999
10/10/2024 05:00	0.964844	3.4	0	0	0	0	0	999	999
10/10/2024 05:15	0.855469	3.6	0	0	0	0	0	999	999
10/10/2024 05:30	0.855469	3.6	0	0	0	0	0	999	999
10/10/2024 05:45	0.855469	3.5	0	0	0	0	0	999	999
10/10/2024 06:00	0.855469	3.6	0	0	0	0	0	999	999
10/10/2024 06:15	0.964844	3.7	0	0	0	0	0	999	999
10/10/2024 06:30	0.964844	3.6	0	0	0	0	0	999	999
10/10/2024 06:45	0.964844	3.7	0	0	0	0	0	999	999
10/10/2024 07:00	0.996094	3.6	0	0	0	0	0	999	999

10/10/2024 07:15	1	3.7	0	0	0	1	0	999	999
10/10/2024 07:30	1	3.7	0	0	0	0	0	999	999
10/10/2024 07:45	1	3.6	0	0	0	0	0	999	999
10/10/2024 08:00	1	3.6	0	0	0	0	0	999	999
10/10/2024 08:15	1	3.9	0	0	0	0	1	999	999
10/10/2024 08:30	0.003906	7.5	0	0	0	0	0	999	999
10/10/2024 08:45	0.035156	3.7	0	0	0	0	0	999	999
10/10/2024 09:00	0.144531	3.6	0	0	0	0	0	999	999
10/10/2024 09:15	0.363281	3.7	0	0	0	0	0	999	999
10/10/2024 09:30	0.636719	3.9	0	0	0	0	0	999	999
10/10/2024 09:45	0.855469	3.9	0	0	0	0	0	999	999
10/10/2024 10:00	0.964844	3.6	0	0	0	0	0	999	999
10/10/2024 10:15	0.996094	3.6	0	0	0	0	0	999	999
10/10/2024 10:30	1	4.5	0	0	0	1	0	999	999
10/10/2024 10:45	1	3.6	0	0	0	0	0	999	999
10/10/2024 11:00	1	3.7	0	0	0	0	0	999	999
10/10/2024 11:15	1	3.6	0	0	0	0	0	999	999
10/10/2024 11:30	1	3.6	0	0	0	0	0	999	999
10/10/2024 11:45	1	4.6	0	0	0	0	0	999	999
10/10/2024 12:00	1	3.6	0	0	0	0	0	999	999
10/10/2024 12:15	1	3.7	0	0	0	0	0	999	999
10/10/2024 12:30	1	3.4	0	0	0	0	0	999	999
10/10/2024 12:45	1	3.4	0	0	0	0	1	999	999
10/10/2024 13:00	0.035156	3.1	0	0	0	0	0	999	999
10/10/2024 13:15	0.144531	8.7	0	0	0	0	0	999	999
10/10/2024 13:30	0.363281	3.2	0	0	0	0	0	999	999
10/10/2024 13:45	0.636719	3.3	0	0	0	0	0	999	999
10/10/2024 14:00	0.855469	3.2	0	0	0	0	0	999	999
10/10/2024 14:15	0.964844	3.4	0	0	0	0	0	999	999
10/10/2024 14:30	0.996094	3.3	0	0	0	0	0	999	999
10/10/2024 14:45	1	3.2	0	0	0	1	0	999	999
10/10/2024 15:00	1	3.1	0	0	0	0	0	999	999
10/10/2024 15:15	1	3.2	0	0	0	0	0	999	999
10/10/2024 15:30	1	3.1	0	0	0	0	0	999	999
10/10/2024 15:45	1	3.1	0	0	0	0	0	999	999
10/10/2024 16:00	1	3.3	0	0	0	0	0	999	999

10/10/2024 16:15	1	2.9	0	0	0	0	0	999	999
10/10/2024 16:30	1	3.1	0	0	0	0	0	999	999
10/10/2024 16:45	1	2.9	0	0	0	0	0	999	999
10/10/2024 17:00	1	3.6	0	0	0	0	1	999	999
10/10/2024 17:15	0.003906	3.1	0	0	0	0	0	999	999
10/10/2024 17:30	0.003906	3	0	0	0	0	0	999	999
10/10/2024 17:45	0.003906	3.7	0	0	0	0	0	999	999
10/10/2024 18:00	0.003906	3.2	0	0	0	0	0	999	999
10/10/2024 18:15	0.003906	3	0	0	0	0	0	999	999
10/10/2024 18:30	0.035156	3.4	0	0	0	0	0	999	999
10/10/2024 18:45	0.144531	3.1	0	0	0	0	0	999	999
10/10/2024 19:00	0.363281	2.9	0	0	0	0	0	999	999
10/10/2024 19:15	0.636719	2.9	0	0	0	0	0	999	999
10/10/2024 19:30	0.855469	3.1	0	0	0	0	0	999	999
10/10/2024 19:45	0.964844	3.1	0	0	0	0	0	999	999
10/10/2024 20:00	0.996094	6.9	0	0	0	0	0	999	999
10/10/2024 20:15	1	3.1	0	0	0	1	0	999	999
10/10/2024 20:30	1	3.2	0	0	0	0	0	999	999
10/10/2024 20:45	1	3.1	0	0	0	0	0	999	999
10/10/2024 21:00	1	3.2	0	0	0	0	0	999	999
10/10/2024 21:15	1	3	0	0	0	0	0	999	999
10/10/2024 21:30	1	5.3	0	0	0	0	0	999	999
10/10/2024 21:45	1	3.3	0	0	0	0	0	999	999
10/10/2024 22:00	1	3.1	0	0	0	0	0	999	999
10/10/2024 22:15	1	3.1	0	0	0	0	0	999	999
10/10/2024 22:30	1	3.1	0	0	0	0	1	999	999
10/10/2024 22:45	0.035156	3.2	0	0	0	0	0	999	999
10/10/2024 23:00	0.144531	3.2	0	0	0	0	0	999	999
10/10/2024 23:15	0.363281	3.2	0	0	0	0	0	999	999
10/10/2024 23:30	0.636719	3.7	0	0	0	0	0	999	999
10/10/2024 23:45	0.855469	3.2	0	0	0	0	0	999	999
11/10/2024 00:00	0.964844	3.6	0	0	0	0	0	999	999
11/10/2024 00:15	0.996094	6.2	0	0	0	0	0	999	999
11/10/2024 00:30	1	3.4	0	0	0	1	0	999	999
11/10/2024 00:45	1	5.1	0	0	0	0	0	999	999
11/10/2024 01:00	1	10	0	0	0	0	0	999	999

11/10/2024 01:15	1	24.5	1	1	0	0	0	999	999
11/10/2024 01:30	1	31.4	1	0	0	0	0	999	999
11/10/2024 01:45	1	20.3	1	0	0	0	0	999	999
11/10/2024 02:00	1	14.2	1	0	0	0	0	999	999
11/10/2024 02:15	1	6.2	1	0	0	0	0	999	999
11/10/2024 02:30	1	4.7	1	0	0	0	0	999	999
11/10/2024 02:45	1	8	1	0	0	0	1	999	999
11/10/2024 03:00	0.035156	4.7	1	0	0	0	0	999	999
11/10/2024 03:15	0.144531	6.6	1	0	0	0	0	999	999
11/10/2024 03:30	0.363281	15.6	1	0	0	0	0	999	999
11/10/2024 03:45	0.636719	162.3	1	0	0	0	0	999	999
11/10/2024 04:00	0.855469	33.9	1	0	0	0	0	999	999
11/10/2024 04:15	0.964844	134.1	1	0	0	0	0	999	999
11/10/2024 04:30	0.996094	133.8	1	0	0	0	0	999	999
11/10/2024 04:45	1	201.8	1	0	0	1	0	999	999
11/10/2024 05:00	1	202.8	1	0	0	0	0	999	999
11/10/2024 05:15	1	105.4	1	0	0	0	0	999	999
11/10/2024 05:30	1	211.1	1	0	0	0	0	999	999
11/10/2024 05:45	1	121.6	1	0	0	0	0	999	999
11/10/2024 06:00	1	152.5	1	0	0	0	0	999	999
11/10/2024 06:15	1	139.2	1	0	0	0	0	999	999
11/10/2024 06:30	1	77.3	1	0	0	0	0	999	999
11/10/2024 06:45	1	99.1	1	0	0	0	0	999	999
11/10/2024 07:00	1	68.5	1	0	0	0	1	999	999
11/10/2024 07:15	0.003906	54.9	1	0	0	0	0	999	999
11/10/2024 07:30	0.003906	104.6	1	0	0	0	0	999	999
11/10/2024 07:45	0.003906	72.3	1	0	0	0	0	999	999
11/10/2024 08:00	0.003906	74.4	1	0	0	0	0	999	999
11/10/2024 08:15	0.003906	62.4	1	0	0	0	0	999	999
11/10/2024 08:30	0.003906	181.2	1	0	0	0	0	999	999
11/10/2024 08:45	0.035156	219.3	1	0	0	0	0	999	999
11/10/2024 09:00	0.144531	211.9	1	0	0	0	0	999	999
11/10/2024 09:15	0.363281	175.3	1	0	0	0	0	999	999
11/10/2024 09:30	0.636719	91.5	1	0	0	0	0	999	999
11/10/2024 09:45	0.636719	212.3	1	0	0	0	0	999	999
11/10/2024 10:00	0.855469	212.9	1	0	0	0	0	999	999

11/10/2024 10:15	0.964844	125.3	1	0	0	0	0	999	999
11/10/2024 10:30	0.964844	98.7	1	0	0	0	0	999	999
11/10/2024 10:45	0.855469	52.4	1	0	0	0	0	999	999
11/10/2024 11:00	0.855469	47.1	1	0	0	0	0	999	999
11/10/2024 11:15	0.855469	97.1	1	0	0	0	0	999	999
11/10/2024 11:30	0.855469	117.3	1	0	0	0	0	999	999
11/10/2024 11:45	0.964844	118.3	1	0	0	0	0	999	999
11/10/2024 12:00	0.964844	103.1	1	0	0	0	0	999	999
11/10/2024 12:15	0.964844	87.2	1	0	0	0	0	999	999
11/10/2024 12:30	0.996094	136.7	1	0	0	0	0	999	999
11/10/2024 12:45	1	46.2	1	0	0	1	0	999	999
11/10/2024 13:00	1	137.6	1	0	0	0	0	999	999
11/10/2024 13:15	1	65	1	0	0	0	0	999	999
11/10/2024 13:30	1	144.4	1	0	0	0	0	999	999
11/10/2024 13:45	1	101.9	1	0	0	0	0	999	999
11/10/2024 14:00	1	133.1	1	0	0	0	1	999	999
11/10/2024 14:15	0.003906	83	1	0	0	0	0	999	999
11/10/2024 14:30	0.035156	170.3	1	0	0	0	0	999	999
11/10/2024 14:45	0.144531	31.8	1	0	0	0	0	999	999
11/10/2024 15:00	0.363281	101.8	1	0	0	0	0	999	999
11/10/2024 15:15	0.636719	64.2	1	0	0	0	0	999	999
11/10/2024 15:30	0.855469	56.9	1	0	0	0	0	999	999
11/10/2024 15:45	0.964844	141	1	0	0	0	0	999	999
11/10/2024 16:00	0.996094	85.1	1	0	0	0	0	999	999
11/10/2024 16:15	1	70	1	0	0	1	0	999	999
11/10/2024 16:30	1	98.8	1	0	0	0	0	999	999
11/10/2024 16:45	1	73.3	1	0	0	0	0	999	999
11/10/2024 17:00	1	58.1	1	0	0	0	0	999	999
11/10/2024 17:15	1	85	1	0	0	0	0	999	999
11/10/2024 17:30	1	87	1	0	0	0	0	999	999
11/10/2024 17:45	1	94.1	1	0	0	0	0	999	999
11/10/2024 18:00	1	75.5	1	0	0	0	0	999	999
11/10/2024 18:15	1	129.9	1	0	0	0	0	999	999
11/10/2024 18:30	1	73	1	0	0	0	1	999	999
11/10/2024 18:45	0.003906	22.1	1	0	0	0	0	999	999
11/10/2024 19:00	0.035156	79.9	1	0	0	0	0	999	999

11/10/2024 19:15	0.035156	64.5	1	0	0	0	0	999	999
11/10/2024 19:30	0.035156	38.4	1	0	0	0	0	999	999
11/10/2024 19:45	0.144531	63.1	1	0	0	0	0	999	999
11/10/2024 20:00	0.363281	104.4	1	0	0	0	0	999	999
11/10/2024 20:15	0.636719	97.6	1	0	0	0	0	999	999
11/10/2024 20:30	0.855469	108.5	1	0	0	0	0	999	999
11/10/2024 20:45	0.964844	113.4	1	0	0	0	0	999	999
11/10/2024 21:00	0.964844	66.3	1	0	0	0	0	999	999
11/10/2024 21:15	0.996094	112.4	1	0	0	0	0	999	999
11/10/2024 21:30	1	115.6	1	0	0	1	0	999	999
11/10/2024 21:45	1	152.2	1	0	0	0	0	999	999
11/10/2024 22:00	1	99.6	1	0	0	0	0	999	999
11/10/2024 22:15	1	164.5	1	0	0	0	0	999	999
11/10/2024 22:30	1	92.7	1	0	0	0	0	999	999
11/10/2024 22:45	1	77.1	1	0	0	0	0	999	999
11/10/2024 23:00	1	160.3	1	0	0	0	0	999	999
11/10/2024 23:15	1	117.4	1	0	0	0	0	999	999
11/10/2024 23:30	1	101.3	1	0	0	0	1	999	999
11/10/2024 23:45	0.003906	89.1	1	0	0	0	0	999	999
12/10/2024 00:00	0.003906	122.9	1	0	0	0	0	999	999
12/10/2024 00:15	0.003906	114.6	1	0	0	0	0	999	999
12/10/2024 00:30	0.035156	120.4	1	0	0	0	0	999	999
12/10/2024 00:45	0.144531	145.5	1	0	0	0	0	999	999
12/10/2024 01:00	0.363281	138	1	0	0	0	0	999	999
12/10/2024 01:15	0.636719	70.5	1	0	0	0	0	999	999
12/10/2024 01:30	0.855469	115.6	1	0	0	0	0	999	999
12/10/2024 01:45	0.964844	92.2	1	0	0	0	0	999	999
12/10/2024 02:00	0.996094	161.3	1	0	0	0	0	999	999
12/10/2024 02:15	1	180.3	1	0	0	1	0	999	999
12/10/2024 02:30	1	127.8	1	0	0	0	0	999	999
12/10/2024 02:45	1	179.7	1	0	0	0	0	999	999
12/10/2024 03:00	1	149	1	0	0	0	0	999	999
12/10/2024 03:15	1	210.9	1	0	0	0	0	999	999
12/10/2024 03:30	1	262	1	0	0	0	0	999	999
12/10/2024 03:45	1	188.7	1	0	0	0	0	999	999
12/10/2024 04:00	1	214.4	1	0	0	0	0	999	999

12/10/2024 04:15	1	245.4	1	0	0	0	0	999	999
12/10/2024 04:30	1	146.6	1	0	0	0	1	999	999
12/10/2024 04:45	0.035156	292.2	1	0	0	0	0	999	999
12/10/2024 05:00	0.144531	251.7	1	0	0	0	0	999	999
12/10/2024 05:15	0.363281	291.2	1	0	0	0	0	999	999
12/10/2024 05:30	0.636719	195.3	1	0	0	0	0	999	999
12/10/2024 05:45	0.855469	176.2	1	0	0	0	0	999	999
12/10/2024 06:00	0.964844	225.5	1	0	0	0	0	999	999
12/10/2024 06:15	0.996094	341.7	1	0	0	0	0	999	999
12/10/2024 06:30	1	309.3	1	0	0	1	0	999	999
12/10/2024 06:45	1	307.9	1	0	0	0	0	999	999
12/10/2024 07:00	1	301.5	1	0	0	0	0	999	999
12/10/2024 07:15	1	334.4	1	0	0	0	0	999	999
12/10/2024 07:30	1	265.6	1	0	0	0	0	999	999
12/10/2024 07:45	1	266.1	1	0	0	0	0	999	999
12/10/2024 08:00	1	268.9	1	0	0	0	0	999	999
12/10/2024 08:15	1	239.7	1	0	0	0	0	999	999
12/10/2024 08:30	1	209.5	1	0	0	0	0	999	999
12/10/2024 08:45	1	223.3	1	0	0	0	1	999	999
12/10/2024 09:00	0.003906	222.2	1	0	0	0	0	999	999
12/10/2024 09:15	0.003906	240.6	1	0	0	0	0	999	999
12/10/2024 09:30	0.003906	325.3	1	0	0	0	0	999	999
12/10/2024 09:45	0.035156	341.6	1	0	0	0	0	999	999
12/10/2024 10:00	0.144531	199.7	1	0	0	0	0	999	999
12/10/2024 10:15	0.363281	267.6	1	0	0	0	0	999	999
12/10/2024 10:30	0.636719	223.7	1	0	0	0	0	999	999
12/10/2024 10:45	0.855469	188	1	0	0	0	0	999	999
12/10/2024 11:00	0.964844	269.1	1	0	0	0	0	999	999
12/10/2024 11:15	0.996094	206.1	1	0	0	0	0	999	999
12/10/2024 11:30	1	170.9	1	0	0	1	0	999	999
12/10/2024 11:45	1	143.9	1	0	0	0	0	999	999
12/10/2024 12:00	1	173.8	1	0	0	0	0	999	999
12/10/2024 12:15	1	147	1	0	0	0	0	999	999
12/10/2024 12:30	1	196.7	1	0	0	0	0	999	999
12/10/2024 12:45	1	160.8	1	0	0	0	0	999	999
12/10/2024 13:00	1	204.7	1	0	0	0	0	999	999

12/10/2024 13:15	1	153.2	1	0	0	0	0	999	999
12/10/2024 13:30	1	118.2	1	0	0	0	0	999	999
12/10/2024 13:45	1	180.6	1	0	0	0	1	999	999
12/10/2024 14:00	0.003906	175.9	1	0	0	0	0	999	999
12/10/2024 14:15	0.003906	153.9	1	0	0	0	0	999	999
12/10/2024 14:30	0.003906	170.5	1	0	0	0	0	999	999
12/10/2024 14:45	0.035156	172.9	1	0	0	0	0	999	999
12/10/2024 15:00	0.144531	239.6	1	0	0	0	0	999	999
12/10/2024 15:15	0.363281	249.4	1	0	0	0	0	999	999
12/10/2024 15:30	0.636719	247.6	1	0	0	0	0	999	999
12/10/2024 15:45	0.855469	184.5	1	0	0	0	0	999	999
12/10/2024 16:00	0.964844	194.8	1	0	0	0	0	999	999
12/10/2024 16:15	0.996094	200.7	1	0	0	0	0	999	999
12/10/2024 16:30	1	218.3	1	0	0	1	0	999	999
12/10/2024 16:45	1	172.1	1	0	0	0	0	999	999
12/10/2024 17:00	1	132.8	1	0	0	0	0	999	999
12/10/2024 17:15	1	307	1	0	0	0	0	999	999
12/10/2024 17:30	1	209.2	1	0	0	0	0	999	999
12/10/2024 17:45	1	151.7	1	0	0	0	0	999	999
12/10/2024 18:00	1	174	1	0	0	0	0	999	999
12/10/2024 18:15	1	101.4	1	0	0	0	0	999	999
12/10/2024 18:30	1	116.2	1	0	0	0	0	999	999
12/10/2024 18:45	1	198.2	1	0	0	0	1	999	999
12/10/2024 19:00	0.003906	131.4	1	0	0	0	0	999	999
12/10/2024 19:15	0.003906	207.7	1	0	0	0	0	999	999
12/10/2024 19:30	0.035156	143.9	1	0	0	0	0	999	999
12/10/2024 19:45	0.144531	159.1	1	0	0	0	0	999	999
12/10/2024 20:00	0.363281	197.9	1	0	0	0	0	999	999
12/10/2024 20:15	0.636719	161.3	1	0	0	0	0	999	999
12/10/2024 20:30	0.855469	178.1	1	0	0	0	0	999	999
12/10/2024 20:45	0.964844	219.5	1	0	0	0	0	999	999
12/10/2024 21:00	0.996094	202.6	1	0	0	0	0	999	999
12/10/2024 21:15	1	222.1	1	0	0	1	0	999	999
12/10/2024 21:30	1	373.4	1	0	0	0	0	999	999
12/10/2024 21:45	1	238.5	1	0	0	0	0	999	999
12/10/2024 22:00	1	287.9	1	0	0	0	0	999	999

12/10/2024 22:15	1	280.7	1	0	0	0	0	999	999
12/10/2024 22:30	1	195.8	1	0	0	0	0	999	999
12/10/2024 22:45	1	126.8	1	0	0	0	0	999	999
12/10/2024 23:00	1	158.7	1	0	0	0	0	999	999
12/10/2024 23:15	1	151.7	1	0	0	0	0	999	999
12/10/2024 23:30	1	241.2	1	0	0	0	1	999	999
12/10/2024 23:45	0.035156	91	1	0	0	0	0	999	999
13/10/2024 00:00	0.144531	132.2	1	0	0	0	0	999	999
13/10/2024 00:15	0.363281	150.3	1	0	0	0	0	999	999
13/10/2024 00:30	0.636719	174.6	1	0	0	0	0	999	999
13/10/2024 00:45	0.855469	227.2	1	0	0	0	0	999	999
13/10/2024 01:00	0.964844	143.9	1	0	0	0	0	999	999
13/10/2024 01:15	0.996094	156.9	1	0	0	0	0	999	999
13/10/2024 01:30	1	232.3	1	0	0	1	0	999	999
13/10/2024 01:45	1	203.9	1	0	0	0	0	999	999
13/10/2024 02:00	1	179.2	1	0	0	0	0	999	999
13/10/2024 02:15	1	123.8	1	0	0	0	0	999	999
13/10/2024 02:30	1	193.8	1	0	0	0	0	999	999
13/10/2024 02:45	1	213.5	1	0	0	0	0	999	999
13/10/2024 03:00	1	210.9	1	0	0	0	0	999	999
13/10/2024 03:15	1	289.7	1	0	0	0	0	999	999
13/10/2024 03:30	1	210.4	1	0	0	0	0	999	999
13/10/2024 03:45	1	186	1	0	0	0	1	999	999
13/10/2024 04:00	0.035156	201.3	1	0	0	0	0	999	999
13/10/2024 04:15	0.144531	200.9	1	0	0	0	0	999	999
13/10/2024 04:30	0.363281	398.2	1	0	0	0	0	999	999
13/10/2024 04:45	0.636719	222.9	1	0	0	0	0	999	999
13/10/2024 05:00	0.855469	160.7	1	0	0	0	0	999	999
13/10/2024 05:15	0.964844	200.1	1	0	0	0	0	999	999
13/10/2024 05:30	0.996094	234.4	1	0	0	0	0	999	999
13/10/2024 05:45	1	279.2	1	0	0	1	0	999	999
13/10/2024 06:00	1	249.4	1	0	0	0	0	999	999
13/10/2024 06:15	1	189.8	1	0	0	0	0	999	999
13/10/2024 06:30	1	264.7	1	0	0	0	0	999	999
13/10/2024 06:45	1	222.2	1	0	0	0	0	999	999
13/10/2024 07:00	1	281.9	1	0	0	0	0	999	999

13/10/2024 07:15	1	251.4	1	0	0	0	0	999	999
13/10/2024 07:30	1	303.8	1	0	0	0	0	999	999
13/10/2024 07:45	1	269.1	1	0	0	0	0	999	999
13/10/2024 08:00	1	243.5	1	0	0	0	1	999	999
13/10/2024 08:15	0.035156	360.3	1	0	0	0	0	999	999
13/10/2024 08:30	0.144531	310.5	1	0	0	0	0	999	999
13/10/2024 08:45	0.363281	230.5	1	0	0	0	0	999	999
13/10/2024 09:00	0.636719	231.9	1	0	0	0	0	999	999
13/10/2024 09:15	0.855469	265.8	1	0	0	0	0	999	999
13/10/2024 09:30	0.964844	244.1	1	0	0	0	0	999	999
13/10/2024 09:45	0.996094	232.6	1	0	0	0	0	999	999
13/10/2024 10:00	1	215.7	1	0	0	1	0	999	999
13/10/2024 10:15	1	237	1	0	0	0	0	999	999
13/10/2024 10:30	1	166	1	0	0	0	0	999	999
13/10/2024 10:45	1	214.9	1	0	0	0	0	999	999
13/10/2024 11:00	1	99.6	1	0	0	0	0	999	999
13/10/2024 11:15	1	210.9	1	0	0	0	0	999	999
13/10/2024 11:30	1	177.9	1	0	0	0	0	999	999
13/10/2024 11:45	1	183.5	1	0	0	0	0	999	999
13/10/2024 12:00	1	160.8	1	0	0	0	0	999	999
13/10/2024 12:15	1	156.1	1	0	0	0	1	999	999
13/10/2024 12:30	0.003906	215.2	1	0	0	0	0	999	999
13/10/2024 12:45	0.003906	166.6	1	0	0	0	0	999	999
13/10/2024 13:00	0.003906	170.8	1	0	0	0	0	999	999
13/10/2024 13:15	0.003906	213.3	1	0	0	0	0	999	999
13/10/2024 13:30	0.003906	214.6	1	0	0	0	0	999	999
13/10/2024 13:45	0.003906	268.8	1	0	0	0	0	999	999
13/10/2024 14:00	0.003906	241.6	1	0	0	0	0	999	999
13/10/2024 14:15	0.003906	236.5	1	0	0	0	0	999	999
13/10/2024 14:30	0.003906	260.8	1	0	0	0	0	999	999
13/10/2024 14:45	0.035156	257.9	1	0	0	0	0	999	999
13/10/2024 15:00	0.144531	359.9	1	0	0	0	0	999	999
13/10/2024 15:15	0.363281	343.8	1	0	0	0	0	999	999
13/10/2024 15:30	0.636719	276.7	1	0	0	0	0	999	999
13/10/2024 15:45	0.855469	260.6	1	0	0	0	0	999	999
13/10/2024 16:00	0.964844	314.7	1	0	0	0	0	999	999

13/10/2024 16:15	0.996094	293.3	1	0	0	0	0	999	999
13/10/2024 16:30	1	258.7	1	0	0	1	0	999	999
13/10/2024 16:45	1	270.7	1	0	0	0	0	999	999
13/10/2024 17:00	1	277.1	1	0	0	0	0	999	999
13/10/2024 17:15	1	235.7	1	0	0	0	0	999	999
13/10/2024 17:30	1	200	1	0	0	0	0	999	999
13/10/2024 17:45	1	222.7	1	0	0	0	1	999	999
13/10/2024 18:00	0.996094	232.1	1	0	0	0	0	999	999
13/10/2024 18:15	0.964844	205.4	1	0	0	0	0	999	999
13/10/2024 18:30	0.855469	190.7	1	0	0	0	0	999	999
13/10/2024 18:45	0.636719	202.4	1	0	0	0	0	999	999
13/10/2024 19:00	0.363281	272	1	0	0	0	0	999	999
13/10/2024 19:15	0.363281	301.8	1	0	0	0	0	999	999
13/10/2024 19:30	0.363281	175.1	1	0	0	0	0	999	999
13/10/2024 19:45	0.144531	273.7	1	0	0	0	0	999	999
13/10/2024 20:00	0.363281	302.7	1	0	0	0	0	999	999
13/10/2024 20:15	0.636719	335.5	1	0	0	0	0	999	999
13/10/2024 20:30	0.855469	363.7	1	0	0	0	0	999	999
13/10/2024 20:45	0.964844	289.7	1	0	0	0	0	999	999
13/10/2024 21:00	0.996094	375.6	1	0	0	0	0	999	999
13/10/2024 21:15	0.996094	377.5	1	0	0	0	0	999	999
13/10/2024 21:30	0.996094	381.4	1	0	0	0	0	999	999
13/10/2024 21:45	1	377.1	1	0	0	1	0	999	999
13/10/2024 22:00	1	421.7	1	0	0	0	0	999	999
13/10/2024 22:15	1	438.7	1	0	0	0	0	999	999
13/10/2024 22:30	1	379.5	1	0	0	0	0	999	999
13/10/2024 22:45	1	462.9	1	0	0	0	0	999	999
13/10/2024 23:00	1	457.4	1	0	0	0	0	999	999
13/10/2024 23:15	1	439.6	1	0	0	0	0	999	999
13/10/2024 23:30	1	628.9	1	0	0	0	1	999	999
13/10/2024 23:45	0.035156	412.7	1	0	0	0	0	999	999
14/10/2024 00:00	0.144531	480.2	1	0	0	0	0	999	999
14/10/2024 00:15	0.363281	476.8	1	0	0	0	0	999	999
14/10/2024 00:30	0.636719	487.7	1	0	0	0	0	999	999
14/10/2024 00:45	0.855469	420.2	1	0	0	0	0	999	999
14/10/2024 01:00	0.964844	557.9	1	0	0	0	0	999	999

14/10/2024 01:15	0.996094	591.6	1	0	0	0	0	999	999
14/10/2024 01:30	1	563	1	0	0	1	0	999	999
14/10/2024 01:45	1	667.8	1	0	0	0	0	999	999
14/10/2024 02:00	1	549	1	0	0	0	0	999	999
14/10/2024 02:15	1	557.9	1	0	0	0	0	999	999
14/10/2024 02:30	1	731.8	1	0	0	0	0	999	999
14/10/2024 02:45	1	641.1	1	0	0	0	0	999	999
14/10/2024 03:00	1	493.9	1	0	0	0	0	999	999
14/10/2024 03:15	1	755.1	1	0	0	0	0	999	999
14/10/2024 03:30	1	554.5	1	0	0	0	0	999	999
14/10/2024 03:45	1	709.7	1	0	0	0	1	999	999
14/10/2024 04:00	0.035156	537.7	1	0	0	0	0	999	999
14/10/2024 04:15	0.035156	744.8	1	0	0	0	0	999	999
14/10/2024 04:30	0.035156	721.6	1	0	0	0	0	999	999
14/10/2024 04:45	0.144531	457	1	0	0	0	0	999	999
14/10/2024 05:00	0.363281	523.4	1	0	0	0	0	999	999
14/10/2024 05:15	0.636719	547.3	1	0	0	0	0	999	999
14/10/2024 05:30	0.855469	555.5	1	0	0	0	0	999	999
14/10/2024 05:45	0.964844	592.2	1	0	0	0	0	999	999
14/10/2024 06:00	0.964844	697.2	1	0	0	0	0	999	999
14/10/2024 06:15	0.996094	751.2	1	0	0	0	0	999	999
14/10/2024 06:30	1	509.8	1	0	0	1	0	999	999
14/10/2024 06:45	1	452	1	0	0	0	0	999	999
14/10/2024 07:00	1	547.4	1	0	0	0	0	999	999
14/10/2024 07:15	1	613	1	0	0	0	0	999	999
14/10/2024 07:30	1	644.6	1	0	0	0	0	999	999
14/10/2024 07:45	1	500.2	1	0	0	0	0	999	999
14/10/2024 08:00	1	489.3	1	0	0	0	0	999	999
14/10/2024 08:15	1	539.6	1	0	0	0	0	999	999
14/10/2024 08:30	1	448	1	0	0	0	1	999	999
14/10/2024 08:45	0.035156	479.4	1	0	0	0	0	999	999
14/10/2024 09:00	0.144531	512	1	0	0	0	0	999	999
14/10/2024 09:15	0.363281	564.2	1	0	0	0	0	999	999
14/10/2024 09:30	0.636719	518.4	1	0	0	0	0	999	999
14/10/2024 09:45	0.855469	608.3	1	0	0	0	0	999	999
14/10/2024 10:00	0.964844	467.4	1	0	0	0	0	999	999

14/10/2024 10:15	0.996094	422.3	1	0	0	0	0	999	999
14/10/2024 10:30	1	652.8	1	0	0	1	0	999	999
14/10/2024 10:45	1	576.6	1	0	0	0	0	999	999
14/10/2024 11:00	1	741.8	1	0	0	0	0	999	999
14/10/2024 11:15	1	519.4	1	0	0	0	0	999	999
14/10/2024 11:30	1	456.1	1	0	0	0	0	999	999
14/10/2024 11:45	1	529.9	1	0	0	0	0	999	999
14/10/2024 12:00	1	556.3	1	0	0	0	0	999	999
14/10/2024 12:15	1	557.2	1	0	0	0	0	999	999
14/10/2024 12:30	1	577.5	1	0	0	0	0	999	999
14/10/2024 12:45	1	416.1	1	0	0	0	1	999	999
14/10/2024 13:00	0.035156	278.3	1	0	0	0	0	999	999
14/10/2024 13:15	0.144531	620.9	1	0	0	0	0	999	999
14/10/2024 13:30	0.363281	304.8	1	0	0	0	0	999	999
14/10/2024 13:45	0.636719	338.6	1	0	0	0	0	999	999
14/10/2024 14:00	0.855469	375.1	1	0	0	0	0	999	999
14/10/2024 14:15	0.964844	321.9	1	0	0	0	0	999	999
14/10/2024 14:30	0.996094	343.5	1	0	0	0	0	999	999
14/10/2024 14:45	1	320.6	1	0	0	1	0	999	999
14/10/2024 15:00	1	362.8	1	0	0	0	0	999	999
14/10/2024 15:15	1	473.8	1	0	0	0	0	999	999
14/10/2024 15:30	1	357.7	1	0	0	0	0	999	999
14/10/2024 15:45	1	246	1	0	0	0	0	999	999
14/10/2024 16:00	1	295.2	1	0	0	0	0	999	999
14/10/2024 16:15	1	265.3	1	0	0	0	0	999	999
14/10/2024 16:30	1	427.6	1	0	0	0	0	999	999
14/10/2024 16:45	1	381.3	1	0	0	0	0	999	999
14/10/2024 17:00	1	429.2	1	0	0	0	1	999	999
14/10/2024 17:15	0.035156	353.5	1	0	0	0	0	999	999
14/10/2024 17:30	0.144531	509.4	1	0	0	0	0	999	999
14/10/2024 17:45	0.363281	411	1	0	0	0	0	999	999
14/10/2024 18:00	0.636719	422.7	1	0	0	0	0	999	999
14/10/2024 18:15	0.855469	372.3	1	0	0	0	0	999	999
14/10/2024 18:30	0.964844	383.8	1	0	0	0	0	999	999
14/10/2024 18:45	0.996094	429.8	1	0	0	0	0	999	999
14/10/2024 19:00	1	385.6	1	0	0	1	0	999	999

14/10/2024 19:15	1	426.8	1	0	0	0	0	999	999
14/10/2024 19:30	1	342.1	1	0	0	0	0	999	999
14/10/2024 19:45	1	531.3	1	0	0	0	0	999	999
14/10/2024 20:00	1	461.9	1	0	0	0	0	999	999
14/10/2024 20:15	1	408.8	1	0	0	0	0	999	999
14/10/2024 20:30	1	334.5	1	0	0	0	0	999	999
14/10/2024 20:45	1	476.5	1	0	0	0	0	999	999
14/10/2024 21:00	1	494.1	1	0	0	0	0	999	999
14/10/2024 21:15	1	652.1	1	0	0	0	1	999	999
14/10/2024 21:30	0.035156	442	1	0	0	0	0	999	999
14/10/2024 21:45	0.144531	361.2	1	0	0	0	0	999	999
14/10/2024 22:00	0.363281	484.3	1	0	0	0	0	999	999
14/10/2024 22:15	0.636719	432.9	1	0	0	0	0	999	999
14/10/2024 22:30	0.855469	472.3	1	0	0	0	0	999	999
14/10/2024 22:45	0.964844	519.1	1	0	0	0	0	999	999
14/10/2024 23:00	0.996094	720	1	0	0	0	0	999	999
14/10/2024 23:15	1	571.1	1	0	0	1	0	999	999
14/10/2024 23:30	1	426.6	1	0	0	0	0	999	999
14/10/2024 23:45	1	331.8	1	0	0	0	0	999	999
15/10/2024 00:00	1	606.6	1	0	0	0	0	999	999
15/10/2024 00:15	1	445.5	1	0	0	0	0	999	999
15/10/2024 00:30	1	457.7	1	0	0	0	0	999	999
15/10/2024 00:45	1	528.3	1	0	0	0	0	999	999
15/10/2024 01:00	1	643.4	1	0	0	0	0	999	999
15/10/2024 01:15	1	489.8	1	0	0	0	0	999	999
15/10/2024 01:30	1	818.9	1	0	0	0	1	999	999
15/10/2024 01:45	0.035156	585.3	1	0	0	0	0	999	999
15/10/2024 02:00	0.144531	498.4	1	0	0	0	0	999	999
15/10/2024 02:15	0.363281	350.6	1	0	0	0	0	999	999
15/10/2024 02:30	0.363281	409.2	1	0	0	0	0	999	999
15/10/2024 02:45	0.363281	364.6	1	0	0	0	0	999	999
15/10/2024 03:00	0.636719	438.9	1	0	0	0	0	999	999
15/10/2024 03:15	0.636719	709	1	0	0	0	0	999	999
15/10/2024 03:30	0.855469	592.7	1	0	0	0	0	999	999
15/10/2024 03:45	0.855469	567.6	1	0	0	0	0	999	999
15/10/2024 04:00	0.855469	342	1	0	0	0	0	999	999

15/10/2024 04:15	0.855469	642.5	1	0	0	0	0	999	999
15/10/2024 04:30	0.964844	945.8	1	0	0	0	0	999	999
15/10/2024 04:45	0.996094	452.5	1	0	0	0	0	999	999
15/10/2024 05:00	0.964844	344.1	1	0	0	0	0	999	999
15/10/2024 05:15	0.964844	518.4	1	0	0	0	0	999	999
15/10/2024 05:30	0.855469	753.1	1	0	0	0	0	999	999
15/10/2024 05:45	0.855469	900.2	1	0	0	0	0	999	999
15/10/2024 06:00	0.855469	547.3	1	0	0	0	0	999	999
15/10/2024 06:15	0.636719	583.8	1	0	0	0	0	999	999
15/10/2024 06:30	0.363281	607.9	1	0	0	0	0	999	999
15/10/2024 06:45	0.363281	658.3	1	0	0	0	0	999	999
15/10/2024 07:00	0.636719	650.1	1	0	0	0	0	999	999
15/10/2024 07:15	0.855469	671.3	1	0	0	0	0	999	999
15/10/2024 07:30	0.964844	518.3	1	0	0	0	0	999	999
15/10/2024 07:45	0.855469	679	1	0	0	0	0	999	999
15/10/2024 08:00	0.855469	489.8	1	0	0	0	0	999	999
15/10/2024 08:15	0.855469	662	1	0	0	0	0	999	999
15/10/2024 08:30	0.964844	305.7	1	0	0	0	0	999	999
15/10/2024 08:45	0.964844	586.8	1	0	0	0	0	999	999
15/10/2024 09:00	0.964844	339.8	1	0	0	0	0	999	999
15/10/2024 09:15	0.964844	371	1	0	0	0	0	999	999
15/10/2024 09:30	0.855469	202.5	1	0	0	0	0	999	999
15/10/2024 09:45	0.964844	890.9	1	0	0	0	0	999	999
15/10/2024 10:00	0.964844	266	1	0	0	0	0	999	999
15/10/2024 10:15	0.996094	506.7	1	0	0	0	0	999	999
15/10/2024 10:30	0.996094	350.5	1	0	0	0	0	999	999
15/10/2024 10:45	0.996094	309	1	0	0	0	0	999	999
15/10/2024 11:00	0.996094	218.3	1	0	0	0	0	999	999
15/10/2024 11:15	0.996094	270.8	1	0	0	0	0	999	999
15/10/2024 11:30	0.035156	267.6	1	0	0	0	0	999	999
15/10/2024 11:45	0.144531	207.1	1	0	0	0	0	999	999
15/10/2024 12:00	0.363281	185.1	1	0	0	0	0	999	999
15/10/2024 12:15	0.636719	136.3	1	0	0	0	0	999	999
15/10/2024 12:30	0.855469	517.9	1	0	0	0	0	999	999
15/10/2024 12:45	0.964844	328.7	1	0	0	0	0	999	999
15/10/2024 13:00	0.996094	324.2	1	0	0	0	0	999	999

15/10/2024 13:15	1	433	1	0	0	1	0	999	999
15/10/2024 13:30	1	226.3	1	0	0	0	0	999	999
15/10/2024 13:45	1	583.3	1	0	0	0	0	999	999
15/10/2024 14:00	1	169.9	1	0	0	0	0	999	999
15/10/2024 14:15	1	147.3	1	0	0	0	0	999	999
15/10/2024 14:30	1	170.8	1	0	0	0	0	999	999
15/10/2024 14:45	1	274	1	0	0	0	0	999	999
15/10/2024 15:00	1	196.7	1	0	0	0	0	999	999
15/10/2024 15:15	1	175.9	1	0	0	0	0	999	999
15/10/2024 15:30	1	168.9	1	0	0	0	1	999	999
15/10/2024 15:45	0.003906	216.1	1	0	0	0	0	999	999
15/10/2024 16:00	0.003906	208.1	1	0	0	0	0	999	999
15/10/2024 16:15	0.035156	226.4	1	0	0	0	0	999	999
15/10/2024 16:30	0.144531	218.3	1	0	0	0	0	999	999
15/10/2024 16:45	0.363281	286.3	1	0	0	0	0	999	999
15/10/2024 17:00	0.636719	263.5	1	0	0	0	0	999	999
15/10/2024 17:15	0.855469	268	1	0	0	0	0	999	999
15/10/2024 17:30	0.964844	227.7	1	0	0	0	0	999	999
15/10/2024 17:45	0.996094	315	1	0	0	0	0	999	999
15/10/2024 18:00	1	359.3	1	0	0	1	0	999	999
15/10/2024 18:15	1	325.1	1	0	0	0	0	999	999
15/10/2024 18:30	1	352.6	1	0	0	0	0	999	999
15/10/2024 18:45	1	308.4	1	0	0	0	0	999	999
15/10/2024 19:00	1	262.2	1	0	0	0	0	999	999
15/10/2024 19:15	1	316	1	0	0	0	0	999	999
15/10/2024 19:30	1	374.6	1	0	0	0	0	999	999
15/10/2024 19:45	1	315.6	1	0	0	0	0	999	999
15/10/2024 20:00	1	294.1	1	0	0	0	0	999	999
15/10/2024 20:15	1	344.9	1	0	0	0	1	999	999
15/10/2024 20:30	0.035156	322.7	1	0	0	0	0	999	999
15/10/2024 20:45	0.144531	370.3	1	0	0	0	0	999	999
15/10/2024 21:00	0.363281	339.1	1	0	0	0	0	999	999
15/10/2024 21:15	0.636719	377.3	1	0	0	0	0	999	999
15/10/2024 21:30	0.855469	376.7	1	0	0	0	0	999	999
15/10/2024 21:45	0.964844	394.8	1	0	0	0	0	999	999
15/10/2024 22:00	0.996094	374	1	0	0	0	0	999	999

15/10/2024 22:15	1	412.4	1	0	0	1	0	999	999
15/10/2024 22:30	1	439.5	1	0	0	0	0	999	999
15/10/2024 22:45	1	467.2	1	0	0	0	0	999	999
15/10/2024 23:00	1	440.6	1	0	0	0	0	999	999
15/10/2024 23:15	1	498.2	1	0	0	0	0	999	999
15/10/2024 23:30	1	408.7	1	0	0	0	0	999	999
15/10/2024 23:45	1	380	1	0	0	0	0	999	999
16/10/2024 00:00	1	416	1	0	0	0	0	999	999
16/10/2024 00:15	1	399.4	1	0	0	0	0	999	999
16/10/2024 00:30	1	375	1	0	0	0	1	999	999
16/10/2024 00:45	0.003906	440.9	1	0	0	0	0	999	999
16/10/2024 01:00	0.035156	465.2	1	0	0	0	0	999	999
16/10/2024 01:15	0.144531	445.4	1	0	0	0	0	999	999
16/10/2024 01:30	0.363281	505.3	1	0	0	0	0	999	999
16/10/2024 01:45	0.636719	520.1	1	0	0	0	0	999	999
16/10/2024 02:00	0.855469	528.2	1	0	0	0	0	999	999
16/10/2024 02:15	0.964844	560.4	1	0	0	0	0	999	999
16/10/2024 02:30	0.996094	473.8	1	0	0	0	0	999	999
16/10/2024 02:45	1	480.2	1	0	0	1	0	999	999
16/10/2024 03:00	1	484.8	1	0	0	0	0	999	999
16/10/2024 03:15	1	472.8	1	0	0	0	0	999	999
16/10/2024 03:30	1	480.9	1	0	0	0	0	999	999
16/10/2024 03:45	1	462.7	1	0	0	0	0	999	999
16/10/2024 04:00	1	603.2	1	0	0	0	0	999	999
16/10/2024 04:15	1	625.5	1	0	0	0	0	999	999
16/10/2024 04:30	1	567.9	1	0	0	0	0	999	999
16/10/2024 04:45	1	577.8	1	0	0	0	0	999	999
16/10/2024 05:00	1	515.4	1	0	0	0	1	999	999
16/10/2024 05:15	0.003906	556.8	1	0	0	0	0	999	999
16/10/2024 05:30	0.035156	442.2	1	0	0	0	0	999	999
16/10/2024 05:45	0.035156	499.3	1	0	0	0	0	999	999
16/10/2024 06:00	0.144531	541.2	1	0	0	0	0	999	999
16/10/2024 06:15	0.363281	535.4	1	0	0	0	0	999	999
16/10/2024 06:30	0.636719	471.5	1	0	0	0	0	999	999
16/10/2024 06:45	0.855469	551.6	1	0	0	0	0	999	999
16/10/2024 07:00	0.964844	628.9	1	0	0	0	0	999	999

16/10/2024 07:15	0.996094	541.9	1	0	0	0	0	999	999
16/10/2024 07:30	0.996094	499	1	0	0	0	0	999	999
16/10/2024 07:45	1	797.3	1	0	0	1	0	999	999
16/10/2024 08:00	1	559.3	1	0	0	0	0	999	999
16/10/2024 08:15	1	655.2	1	0	0	0	0	999	999
16/10/2024 08:30	1	636.2	1	0	0	0	0	999	999
16/10/2024 08:45	1	763.7	1	0	0	0	0	999	999
16/10/2024 09:00	1	608.6	1	0	0	0	0	999	999
16/10/2024 09:15	1	806.3	1	0	0	0	0	999	999
16/10/2024 09:30	1	640.8	1	0	0	0	0	999	999
16/10/2024 09:45	1	673.6	1	0	0	0	1	999	999
16/10/2024 10:00	0.003906	568.9	1	0	0	0	0	999	999
16/10/2024 10:15	0.035156	726.6	1	0	0	0	0	999	999
16/10/2024 10:30	0.144531	671.9	1	0	0	0	0	999	999
16/10/2024 10:45	0.363281	371.2	1	0	0	0	0	999	999
16/10/2024 11:00	0.636719	316.7	1	0	0	0	0	999	999
16/10/2024 11:15	0.855469	234.8	1	0	0	0	0	999	999
16/10/2024 11:30	0.964844	157.5	1	0	0	0	0	999	999
16/10/2024 11:45	0.996094	308.7	1	0	0	0	0	999	999
16/10/2024 12:00	1	195.2	1	0	0	1	0	999	999
16/10/2024 12:15	1	233.3	1	0	0	0	0	999	999
16/10/2024 12:30	1	202.1	1	0	0	0	0	999	999
16/10/2024 12:45	1	228.2	1	0	0	0	0	999	999
16/10/2024 13:00	1	163.2	1	0	0	0	0	999	999
16/10/2024 13:15	1	110.1	1	0	0	0	0	999	999
16/10/2024 13:30	1	149.9	1	0	0	0	0	999	999
16/10/2024 13:45	1	161.3	1	0	0	0	0	999	999
16/10/2024 14:00	1	156.3	1	0	0	0	0	999	999
16/10/2024 14:15	1	140	1	0	0	0	1	999	999
16/10/2024 14:30	0.003906	196.2	1	0	0	0	0	999	999
16/10/2024 14:45	0.003906	268.4	1	0	0	0	0	999	999
16/10/2024 15:00	0.003906	253.5	1	0	0	0	0	999	999
16/10/2024 15:15	0.003906	276.5	1	0	0	0	0	999	999
16/10/2024 15:30	0.035156	237	1	0	0	0	0	999	999
16/10/2024 15:45	0.144531	323.6	1	0	0	0	0	999	999
16/10/2024 16:00	0.363281	334.9	1	0	0	0	0	999	999

16/10/2024 16:15	0.636719	415.3	1	0	0	0	0	999	999
16/10/2024 16:30	0.855469	172.2	1	0	0	0	0	999	999
16/10/2024 16:45	0.964844	304	1	0	0	0	0	999	999
16/10/2024 17:00	0.996094	342.4	1	0	0	0	0	999	999
16/10/2024 17:15	1	264.7	1	0	0	1	0	999	999
16/10/2024 17:30	1	186.2	1	0	0	0	0	999	999
16/10/2024 17:45	1	354	1	0	0	0	0	999	999
16/10/2024 18:00	1	332.9	1	0	0	0	0	999	999
16/10/2024 18:15	1	257.7	1	0	0	0	0	999	999
16/10/2024 18:30	1	221.9	1	0	0	0	0	999	999
16/10/2024 18:45	1	201.2	1	0	0	0	1	999	999
16/10/2024 19:00	0.996094	272.3	1	0	0	0	0	999	999
16/10/2024 19:15	0.964844	236.8	1	0	0	0	0	999	999
16/10/2024 19:30	0.855469	271.9	1	0	0	0	0	999	999
16/10/2024 19:45	0.636719	269.1	1	0	0	0	0	999	999
16/10/2024 20:00	0.363281	197.5	1	0	0	0	0	999	999
16/10/2024 20:15	0.363281	199	1	0	0	0	0	999	999
16/10/2024 20:30	0.363281	164.9	1	0	0	0	0	999	999
16/10/2024 20:45	0.363281	149.2	1	0	0	0	0	999	999
16/10/2024 21:00	0.636719	152.1	1	0	0	0	0	999	999
16/10/2024 21:15	0.855469	196.6	1	0	0	0	0	999	999
16/10/2024 21:30	0.964844	160.4	1	0	0	0	0	999	999
16/10/2024 21:45	0.996094	175.2	1	0	0	0	0	999	999
16/10/2024 22:00	1	151.7	1	0	0	1	0	999	999
16/10/2024 22:15	1	160.9	1	0	0	0	0	999	999
16/10/2024 22:30	1	170.4	1	0	0	0	0	999	999
16/10/2024 22:45	1	227.5	1	0	0	0	0	999	999
16/10/2024 23:00	1	181.6	1	0	0	0	0	999	999
16/10/2024 23:15	1	181.9	1	0	0	0	0	999	999
16/10/2024 23:30	1	146.3	1	0	0	0	0	999	999
16/10/2024 23:45	1	214.5	1	0	0	0	0	999	999
17/10/2024 00:00	1	211	1	0	0	0	0	999	999
17/10/2024 00:15	1	216.3	1	0	0	0	1	999	999
17/10/2024 00:30	0.003906	176.2	1	0	0	0	0	999	999
17/10/2024 00:45	0.035156	180.8	1	0	0	0	0	999	999
17/10/2024 01:00	0.144531	169.6	1	0	0	0	0	999	999

17/10/2024 01:15	0.363281	194.8	1	0	0	0	0	999	999
17/10/2024 01:30	0.636719	173.5	1	0	0	0	0	999	999
17/10/2024 01:45	0.855469	189.2	1	0	0	0	0	999	999
17/10/2024 02:00	0.964844	388.4	1	0	0	0	0	999	999
17/10/2024 02:15	0.996094	212.6	1	0	0	0	0	999	999
17/10/2024 02:30	1	193.9	1	0	0	1	0	999	999
17/10/2024 02:45	1	171.6	1	0	0	0	0	999	999
17/10/2024 03:00	1	179.8	1	0	0	0	0	999	999
17/10/2024 03:15	1	251.1	1	0	0	0	0	999	999
17/10/2024 03:30	1	197.1	1	0	0	0	0	999	999
17/10/2024 03:45	1	215.8	1	0	0	0	0	999	999
17/10/2024 04:00	1	251.4	1	0	0	0	0	999	999
17/10/2024 04:15	1	350.6	1	0	0	0	0	999	999
17/10/2024 04:30	1	216.5	1	0	0	0	0	999	999
17/10/2024 04:45	1	333.1	1	0	0	0	1	999	999
17/10/2024 05:00	0.035156	283.5	1	0	0	0	0	999	999
17/10/2024 05:15	0.144531	296.5	1	0	0	0	0	999	999
17/10/2024 05:30	0.363281	400	1	0	0	0	0	999	999
17/10/2024 05:45	0.636719	280	1	0	0	0	0	999	999
17/10/2024 06:00	0.855469	393.7	1	0	0	0	0	999	999
17/10/2024 06:15	0.964844	505.1	1	0	0	0	0	999	999
17/10/2024 06:30	0.996094	351.5	1	0	0	0	0	999	999
17/10/2024 06:45	1	384.4	1	0	0	1	0	999	999
17/10/2024 07:00	1	433.2	1	0	0	0	0	999	999
17/10/2024 07:15	1	438.1	1	0	0	0	0	999	999
17/10/2024 07:30	1	407	1	0	0	0	0	999	999
17/10/2024 07:45	1	487.1	1	0	0	0	0	999	999
17/10/2024 08:00	1	355.1	1	0	0	0	0	999	999
17/10/2024 08:15	1	367.4	1	0	0	0	0	999	999
17/10/2024 08:30	1	294.9	1	0	0	0	0	999	999
17/10/2024 08:45	1	377.8	1	0	0	0	0	999	999
17/10/2024 09:00	1	415.4	1	0	0	0	1	999	999
17/10/2024 09:15	0.035156	410.4	1	0	0	0	0	999	999
17/10/2024 09:30	0.144531	581.3	1	0	0	0	0	999	999
17/10/2024 09:45	0.363281	482.3	1	0	0	0	0	999	999
17/10/2024 10:00	0.636719	453	1	0	0	0	0	999	999

17/10/2024 10:15	0.855469	474.1	1	0	0	0	0	999	999
17/10/2024 10:30	0.964844	426.9	1	0	0	0	0	999	999
17/10/2024 10:45	0.996094	470.2	1	0	0	0	0	999	999
17/10/2024 11:00	1	508.7	1	0	0	1	0	999	999
17/10/2024 11:15	1	482.8	1	0	0	0	0	999	999
17/10/2024 11:30	1	467.2	1	0	0	0	0	999	999
17/10/2024 11:45	1	375.9	1	0	0	0	0	999	999
17/10/2024 12:00	1	380.6	1	0	0	0	0	999	999
17/10/2024 12:15	1	242.3	1	0	0	0	0	999	999
17/10/2024 12:30	1	234.3	1	0	0	0	0	999	999
17/10/2024 12:45	1	130.1	1	0	0	0	0	999	999
17/10/2024 13:00	1	332.6	1	0	0	0	0	999	999
17/10/2024 13:15	1	293.7	1	0	0	0	1	999	999
17/10/2024 13:30	0.035156	221.8	1	0	0	0	0	999	999
17/10/2024 13:45	0.144531	249	1	0	0	0	0	999	999
17/10/2024 14:00	0.363281	321.1	1	0	0	0	0	999	999
17/10/2024 14:15	0.636719	100.2	1	0	0	0	0	999	999
17/10/2024 14:30	0.855469	115.4	1	0	0	0	0	999	999
17/10/2024 14:45	0.964844	141.6	1	0	0	0	0	999	999
17/10/2024 15:00	0.996094	233.8	1	0	0	0	0	999	999
17/10/2024 15:15	1	205.9	1	0	0	1	0	999	999
17/10/2024 15:30	1	349.3	1	0	0	0	0	999	999
17/10/2024 15:45	1	314.5	1	0	0	0	0	999	999
17/10/2024 16:00	1	58.3	1	0	0	0	0	999	999
17/10/2024 16:15	1	161.4	1	0	0	0	0	999	999
17/10/2024 16:30	1	148.3	1	0	0	0	0	999	999
17/10/2024 16:45	1	123.8	1	0	0	0	0	999	999
17/10/2024 17:00	1	73	1	0	0	0	0	999	999
17/10/2024 17:15	1	34.7	1	0	0	0	0	999	999
17/10/2024 17:30	1	105.9	1	0	0	0	1	999	999
17/10/2024 17:45	0.003906	62.6	1	0	0	0	0	999	999
17/10/2024 18:00	0.003906	30.1	1	0	0	0	0	999	999
17/10/2024 18:15	0.003906	107.8	1	0	1	0	0	999	TRUE
17/10/2024 18:30	0.003906	15.7	0	0	0	0	0	999	999
17/10/2024 18:45	0.003906	23.5	0	0	0	0	0	999	999
17/10/2024 19:00	0.003906	38.2	0	0	0	0	0	999	999

17/10/2024 19:15	0.003906	49.8	0	0	0	0	0	999	999
17/10/2024 19:30	0.003906	10.3	0	0	0	0	0	999	999
17/10/2024 19:45	0.003906	8.9	0	0	0	0	0	999	999
17/10/2024 20:00	0.035156	12.2	0	0	0	0	0	999	999
17/10/2024 20:15	0.144531	12.1	0	0	0	0	0	999	999
17/10/2024 20:30	0.363281	50.9	0	0	0	0	0	999	999
17/10/2024 20:45	0.636719	14.5	0	0	0	0	0	999	999
17/10/2024 21:00	0.855469	23.2	0	0	0	0	0	999	999
17/10/2024 21:15	0.964844	39.4	0	0	0	0	0	999	999
17/10/2024 21:30	0.996094	24.9	0	0	0	0	0	999	999
17/10/2024 21:45	1	22.2	0	0	0	1	0	999	999
17/10/2024 22:00	1	17.3	0	0	0	0	0	999	999
17/10/2024 22:15	1	24.1	0	0	0	0	0	999	999
17/10/2024 22:30	1	13.7	0	0	0	0	0	999	999
17/10/2024 22:45	1	22.8	0	0	0	0	0	999	999
17/10/2024 23:00	1	22.7	0	0	0	0	0	999	999
17/10/2024 23:15	1	11.4	0	0	0	0	0	999	999
17/10/2024 23:30	1	18.1	0	0	0	0	0	999	999
17/10/2024 23:45	1	12.3	0	0	0	0	0	999	999
18/10/2024 00:00	1	8.4	0	0	0	0	1	999	999
18/10/2024 00:15	0.003906	8.7	0	0	0	0	0	999	999
18/10/2024 00:30	0.003906	20	0	0	0	0	0	999	999
18/10/2024 00:45	0.003906	14.5	0	0	0	0	0	999	999
18/10/2024 01:00	0.003906	36.5	0	0	0	0	0	999	999
18/10/2024 01:15	0.003906	22.3	0	0	0	0	0	999	999
18/10/2024 01:30	0.003906	19.2	0	0	0	0	0	999	999
18/10/2024 01:45	0.003906	38.3	0	0	0	0	0	999	999
18/10/2024 02:00	0.003906	41.6	0	0	0	0	0	999	999
18/10/2024 02:15	0.035156	9.7	0	0	0	0	0	999	999
18/10/2024 02:30	0.144531	33.5	0	0	0	0	0	999	999
18/10/2024 02:45	0.363281	14.1	0	0	0	0	0	999	999
18/10/2024 03:00	0.636719	23.9	0	0	0	0	0	999	999
18/10/2024 03:15	0.855469	13.1	0	0	0	0	0	999	999
18/10/2024 03:30	0.964844	27.8	0	0	0	0	0	999	999
18/10/2024 03:45	0.996094	24.5	0	0	0	0	0	999	999
18/10/2024 04:00	1	7.9	0	0	0	1	0	999	999

18/10/2024 04:15	1	38.2	0	0	0	0	0	999	999
18/10/2024 04:30	1	26.1	0	0	0	0	0	999	999
18/10/2024 04:45	1	17.4	0	0	0	0	0	999	999
18/10/2024 05:00	1	15.4	0	0	0	0	0	999	999
18/10/2024 05:15	1	15.1	0	0	0	0	0	999	999
18/10/2024 05:30	1	16.8	0	0	0	0	0	999	999
18/10/2024 05:45	1	12.8	0	0	0	0	0	999	999
18/10/2024 06:00	1	8.3	0	0	0	0	0	999	999
18/10/2024 06:15	1	14.1	0	0	0	0	1	999	999
18/10/2024 06:30	0.003906	46.6	0	0	0	0	0	999	999
18/10/2024 06:45	0.035156	8.2	0	0	0	0	0	999	999
18/10/2024 07:00	0.144531	16	0	0	0	0	0	999	999
18/10/2024 07:15	0.363281	60.7	0	0	0	0	0	999	999
18/10/2024 07:30	0.636719	25.8	0	0	0	0	0	999	999
18/10/2024 07:45	0.855469	20.5	0	0	0	0	0	999	999
18/10/2024 08:00	0.964844	8.9	0	0	0	0	0	999	999
18/10/2024 08:15	0.996094	20	0	0	0	0	0	999	999
18/10/2024 08:30	1	8	0	0	0	1	0	999	999
18/10/2024 08:45	1	22.2	0	0	0	0	0	999	999
18/10/2024 09:00	1	22.7	0	0	0	0	0	999	999
18/10/2024 09:15	1	41.2	0	0	0	0	0	999	999
18/10/2024 09:30	1	29.6	0	0	0	0	0	999	999
18/10/2024 09:45	1	28.3	0	0	0	0	0	999	999
18/10/2024 10:00	1	14.2	0	0	0	0	0	999	999
18/10/2024 10:15	1	42.3	0	0	0	0	0	999	999
18/10/2024 10:30	1	20.6	0	0	0	0	0	999	999
18/10/2024 10:45	1	26.7	0	0	0	0	1	999	999
18/10/2024 11:00	0.035156	48	0	0	0	0	0	999	999
18/10/2024 11:15	0.144531	28.4	0	0	0	0	0	999	999
18/10/2024 11:30	0.363281	90	0	0	0	0	0	999	999
18/10/2024 11:45	0.636719	24.2	0	0	0	0	0	999	999
18/10/2024 12:00	0.855469	27.3	0	0	0	0	0	999	999
18/10/2024 12:15	0.964844	52.4	0	0	0	0	0	999	999
18/10/2024 12:30	0.996094	40	0	0	0	0	0	999	999
18/10/2024 12:45	1	109.1	1	1	0	1	0	999	999
18/10/2024 13:00	1	112.5	1	0	0	0	0	999	999

18/10/2024 13:15	1	123.7	1	0	0	0	0	999	999
18/10/2024 13:30	1	115.9	1	0	0	0	0	999	999
18/10/2024 13:45	1	60.4	1	0	0	0	0	999	999
18/10/2024 14:00	1	136.5	1	0	0	0	0	999	999
18/10/2024 14:15	1	90.5	1	0	0	0	0	999	999
18/10/2024 14:30	1	85.4	1	0	0	0	0	999	999
18/10/2024 14:45	1	56.7	1	0	0	0	0	999	999
18/10/2024 15:00	1	54.2	1	0	0	0	1	999	999
18/10/2024 15:15	0.003906	118.1	1	0	0	0	0	999	999
18/10/2024 15:30	0.035156	25.1	1	0	0	0	0	999	999
18/10/2024 15:45	0.144531	15.1	1	0	0	0	0	999	999
18/10/2024 16:00	0.363281	15.3	1	0	0	0	0	999	999
18/10/2024 16:15	0.636719	23.3	1	0	0	0	0	999	999
18/10/2024 16:30	0.855469	12.6	1	0	0	0	0	999	999
18/10/2024 16:45	0.964844	25.8	1	0	0	0	0	999	999
18/10/2024 17:00	0.996094	174.1	1	0	0	0	0	999	999
18/10/2024 17:15	1	83.1	1	0	0	1	0	999	999
18/10/2024 17:30	1	68.6	1	0	0	0	0	999	999
18/10/2024 17:45	1	226	1	0	0	0	0	999	999
18/10/2024 18:00	1	197.5	1	0	0	0	0	999	999
18/10/2024 18:15	1	250.1	1	0	0	0	0	999	999
18/10/2024 18:30	1	206.4	1	0	0	0	0	999	999
18/10/2024 18:45	1	211.4	1	0	0	0	0	999	999
18/10/2024 19:00	1	161.5	1	0	0	0	0	999	999
18/10/2024 19:15	1	127.7	1	0	0	0	0	999	999
18/10/2024 19:30	1	142.5	1	0	0	0	1	999	999
18/10/2024 19:45	0.003906	245.4	1	0	0	0	0	999	999
18/10/2024 20:00	0.035156	156.8	1	0	0	0	0	999	999
18/10/2024 20:15	0.144531	175.2	1	0	0	0	0	999	999
18/10/2024 20:30	0.363281	273.1	1	0	0	0	0	999	999
18/10/2024 20:45	0.636719	267.3	1	0	0	0	0	999	999
18/10/2024 21:00	0.855469	339.4	1	0	0	0	0	999	999
18/10/2024 21:15	0.964844	298	1	0	0	0	0	999	999
18/10/2024 21:30	0.996094	325.1	1	0	0	0	0	999	999
18/10/2024 21:45	1	293.6	1	0	0	1	0	999	999
18/10/2024 22:00	1	588.9	1	0	0	0	0	999	999

18/10/2024 22:15	1	342.7	1	0	0	0	0	999	999
18/10/2024 22:30	1	303.8	1	0	0	0	0	999	999
18/10/2024 22:45	1	461.3	1	0	0	0	0	999	999
18/10/2024 23:00	1	794.7	1	0	0	0	0	999	999
18/10/2024 23:15	1	180.5	1	0	0	0	0	999	999
18/10/2024 23:30	1	394.7	1	0	0	0	0	999	999
18/10/2024 23:45	1	332.8	1	0	0	0	0	999	999
19/10/2024 00:00	1	247.1	1	0	0	0	1	999	999
19/10/2024 00:15	0.035156	16.9	1	0	0	0	0	999	999
19/10/2024 00:30	0.144531	443.7	1	0	0	0	0	999	999
19/10/2024 00:45	0.363281	216.3	1	0	0	0	0	999	999
19/10/2024 01:00	0.636719	337	1	0	0	0	0	999	999
19/10/2024 01:15	0.855469	147.1	1	0	0	0	0	999	999
19/10/2024 01:30	0.964844	162.6	1	0	0	0	0	999	999
19/10/2024 01:45	0.996094	202.3	1	0	0	0	0	999	999
19/10/2024 02:00	1	241.9	1	0	0	1	0	999	999
19/10/2024 02:15	1	162.6	1	0	0	0	0	999	999
19/10/2024 02:30	1	200.8	1	0	0	0	0	999	999
19/10/2024 02:45	1	116.7	1	0	0	0	0	999	999
19/10/2024 03:00	1	142.2	1	0	0	0	0	999	999
19/10/2024 03:15	1	252.8	1	0	0	0	0	999	999
19/10/2024 03:30	1	265.7	1	0	0	0	0	999	999
19/10/2024 03:45	1	250.6	1	0	0	0	0	999	999
19/10/2024 04:00	1	317.9	1	0	0	0	0	999	999
19/10/2024 04:15	1	240.9	1	0	0	0	1	999	999
19/10/2024 04:30	0.003906	215.5	1	0	0	0	0	999	999
19/10/2024 04:45	0.003906	350.1	1	0	0	0	0	999	999
19/10/2024 05:00	0.035156	162.4	1	0	0	0	0	999	999
19/10/2024 05:15	0.144531	88.8	1	0	0	0	0	999	999
19/10/2024 05:30	0.363281	111	1	0	0	0	0	999	999
19/10/2024 05:45	0.636719	619.7	1	0	0	0	0	999	999
19/10/2024 06:00	0.855469	529.6	1	0	0	0	0	999	999
19/10/2024 06:15	0.964844	217.8	1	0	0	0	0	999	999
19/10/2024 06:30	0.996094	186.7	1	0	0	0	0	999	999
19/10/2024 06:45	1	579.6	1	0	0	1	0	999	999
19/10/2024 07:00	1	298.8	1	0	0	0	0	999	999

19/10/2024 07:15	1	121.2	1	0	0	0	0	999	999
19/10/2024 07:30	1	238	1	0	0	0	0	999	999
19/10/2024 07:45	1	69.7	1	0	0	0	0	999	999
19/10/2024 08:00	1	94.3	1	0	0	0	0	999	999
19/10/2024 08:15	1	529.7	1	0	0	0	0	999	999
19/10/2024 08:30	1	548.7	1	0	0	0	0	999	999
19/10/2024 08:45	1	487.5	1	0	0	0	0	999	999
19/10/2024 09:00	1	189.2	1	0	0	0	1	999	999
19/10/2024 09:15	0.003906	142.5	1	0	0	0	0	999	999
19/10/2024 09:30	0.003906	319.5	1	0	0	0	0	999	999
19/10/2024 09:45	0.035156	258.3	1	0	0	0	0	999	999
19/10/2024 10:00	0.144531	203	1	0	0	0	0	999	999
19/10/2024 10:15	0.144531	213	1	0	0	0	0	999	999
19/10/2024 10:30	0.144531	434.4	1	0	0	0	0	999	999
19/10/2024 10:45	0.363281	374.2	1	0	0	0	0	999	999
19/10/2024 11:00	0.636719	272.2	1	0	0	0	0	999	999
19/10/2024 11:15	0.636719	313.9	1	0	0	0	0	999	999
19/10/2024 11:30	0.636719	612	1	0	0	0	0	999	999
19/10/2024 11:45	0.636719	173.7	1	0	0	0	0	999	999
19/10/2024 12:00	0.363281	331.9	1	0	0	0	0	999	999
19/10/2024 12:15	0.363281	440.1	1	0	0	0	0	999	999
19/10/2024 12:30	0.363281	500.3	1	0	0	0	0	999	999
19/10/2024 12:45	0.144531	430.4	1	0	0	0	0	999	999
19/10/2024 13:00	0.144531	390.6	1	0	0	0	0	999	999
19/10/2024 13:15	0.363281	232.9	1	0	0	0	0	999	999
19/10/2024 13:30	0.636719	174.6	1	0	0	0	0	999	999
19/10/2024 13:45	0.636719	194.1	1	0	0	0	0	999	999
19/10/2024 14:00	0.855469	69.7	1	0	0	0	0	999	999
19/10/2024 14:15	0.964844	80.1	1	0	0	0	0	999	999
19/10/2024 14:30	0.996094	86.3	1	0	0	0	0	999	999
19/10/2024 14:45	1	221.8	1	0	0	1	0	999	999
19/10/2024 15:00	1	105.3	1	0	0	0	0	999	999
19/10/2024 15:15	1	126.7	1	0	0	0	0	999	999
19/10/2024 15:30	1	133.6	1	0	0	0	0	999	999
19/10/2024 15:45	1	101.9	1	0	0	0	0	999	999
19/10/2024 16:00	1	95	1	0	0	0	0	999	999

19/10/2024 16:15	1	265.6	1	0	0	0	0	999	999
19/10/2024 16:30	1	265.8	1	0	0	0	0	999	999
19/10/2024 16:45	1	84.7	1	0	0	0	0	999	999
19/10/2024 17:00	1	115.9	1	0	0	0	1	999	999
19/10/2024 17:15	0.003906	128.7	1	0	0	0	0	999	999
19/10/2024 17:30	0.003906	92	1	0	0	0	0	999	999
19/10/2024 17:45	0.003906	88	1	0	0	0	0	999	999
19/10/2024 18:00	0.003906	58.5	1	0	0	0	0	999	999
19/10/2024 18:15	0.035156	59.3	1	0	0	0	0	999	999
19/10/2024 18:30	0.144531	94.9	1	0	0	0	0	999	999
19/10/2024 18:45	0.363281	71.7	1	0	0	0	0	999	999
19/10/2024 19:00	0.636719	57.3	1	0	0	0	0	999	999
19/10/2024 19:15	0.855469	106.5	1	0	0	0	0	999	999
19/10/2024 19:30	0.964844	87.9	1	0	0	0	0	999	999
19/10/2024 19:45	0.996094	121.7	1	0	0	0	0	999	999
19/10/2024 20:00	1	69.9	1	0	0	1	0	999	999
19/10/2024 20:15	1	87	1	0	0	0	0	999	999
19/10/2024 20:30	1	61.9	1	0	0	0	0	999	999
19/10/2024 20:45	1	49.8	1	0	0	0	0	999	999
19/10/2024 21:00	1	107.9	1	0	0	0	0	999	999
19/10/2024 21:15	1	150	1	0	0	0	0	999	999
19/10/2024 21:30	1	188.9	1	0	0	0	0	999	999
19/10/2024 21:45	1	171.8	1	0	0	0	0	999	999
19/10/2024 22:00	1	227.8	1	0	0	0	0	999	999
19/10/2024 22:15	1	245.3	1	0	0	0	1	999	999
19/10/2024 22:30	0.003906	100.7	1	0	0	0	0	999	999
19/10/2024 22:45	0.035156	103.8	1	0	0	0	0	999	999
19/10/2024 23:00	0.144531	152.2	1	0	0	0	0	999	999
19/10/2024 23:15	0.144531	130.5	1	0	0	0	0	999	999
19/10/2024 23:30	0.144531	179.9	1	0	0	0	0	999	999
19/10/2024 23:45	0.363281	79.7	1	0	0	0	0	999	999
20/10/2024 00:00	0.636719	179.9	1	0	0	0	0	999	999
20/10/2024 00:15	0.855469	240.9	1	0	0	0	0	999	999
20/10/2024 00:30	0.964844	154.2	1	0	0	0	0	999	999
20/10/2024 00:45	0.964844	165.5	1	0	0	0	0	999	999
20/10/2024 01:00	0.964844	57.9	1	0	0	0	0	999	999

20/10/2024 01:15	0.996094	168.4	1	0	0	0	0	999	999
20/10/2024 01:30	1	250.8	1	0	0	1	0	999	999
20/10/2024 01:45	1	211.4	1	0	0	0	0	999	999
20/10/2024 02:00	1	146.9	1	0	0	0	0	999	999
20/10/2024 02:15	1	180.4	1	0	0	0	0	999	999
20/10/2024 02:30	1	195.3	1	0	0	0	0	999	999
20/10/2024 02:45	1	167	1	0	0	0	0	999	999
20/10/2024 03:00	1	271.3	1	0	0	0	0	999	999
20/10/2024 03:15	1	246.1	1	0	0	0	1	999	999
20/10/2024 03:30	0.035156	251	1	0	0	0	0	999	999
20/10/2024 03:45	0.144531	190	1	0	0	0	0	999	999
20/10/2024 04:00	0.363281	97.1	1	0	0	0	0	999	999
20/10/2024 04:15	0.636719	284.8	1	0	0	0	0	999	999
20/10/2024 04:30	0.855469	171.5	1	0	0	0	0	999	999
20/10/2024 04:45	0.964844	162.1	1	0	0	0	0	999	999
20/10/2024 05:00	0.996094	126.9	1	0	0	0	0	999	999
20/10/2024 05:15	1	48.2	1	0	0	1	0	999	999
20/10/2024 05:30	1	241.5	1	0	0	0	0	999	999
20/10/2024 05:45	1	165.5	1	0	0	0	0	999	999
20/10/2024 06:00	1	169.6	1	0	0	0	0	999	999
20/10/2024 06:15	1	191.4	1	0	0	0	0	999	999
20/10/2024 06:30	1	122.1	1	0	0	0	0	999	999
20/10/2024 06:45	1	101	1	0	0	0	0	999	999
20/10/2024 07:00	1	131.1	1	0	0	0	0	999	999
20/10/2024 07:15	1	107.6	1	0	0	0	0	999	999
20/10/2024 07:30	1	174	1	0	0	0	1	999	999
20/10/2024 07:45	0.003906	203.6	1	0	0	0	0	999	999
20/10/2024 08:00	0.003906	132.5	1	0	0	0	0	999	999
20/10/2024 08:15	0.003906	87.3	1	0	0	0	0	999	999
20/10/2024 08:30	0.035156	159.5	1	0	0	0	0	999	999
20/10/2024 08:45	0.144531	89.6	1	0	0	0	0	999	999
20/10/2024 09:00	0.363281	91.9	1	0	0	0	0	999	999
20/10/2024 09:15	0.636719	123.7	1	0	0	0	0	999	999
20/10/2024 09:30	0.855469	166.4	1	0	0	0	0	999	999
20/10/2024 09:45	0.964844	157.5	1	0	0	0	0	999	999
20/10/2024 10:00	0.996094	107.2	1	0	0	0	0	999	999

20/10/2024 10:15	1	75.7	1	0	0	1	0	999	999
20/10/2024 10:30	1	127.8	1	0	0	0	0	999	999
20/10/2024 10:45	1	75.8	1	0	0	0	0	999	999
20/10/2024 11:00	1	126.8	1	0	0	0	0	999	999
20/10/2024 11:15	1	72.8	1	0	0	0	0	999	999
20/10/2024 11:30	1	47.2	1	0	0	0	0	999	999
20/10/2024 11:45	1	45.5	1	0	0	0	0	999	999
20/10/2024 12:00	1	93.9	1	0	0	0	0	999	999
20/10/2024 12:15	1	72.5	1	0	0	0	0	999	999
20/10/2024 12:30	1	68.7	1	0	0	0	1	999	999
20/10/2024 12:45	0.035156	96.1	1	0	0	0	0	999	999
20/10/2024 13:00	0.144531	70.7	1	0	0	0	0	999	999
20/10/2024 13:15	0.363281	43.2	1	0	0	0	0	999	999
20/10/2024 13:30	0.636719	266.7	1	0	0	0	0	999	999
20/10/2024 13:45	0.855469	290.2	1	0	0	0	0	999	999
20/10/2024 14:00	0.964844	186	1	0	0	0	0	999	999
20/10/2024 14:15	0.996094	154.8	1	0	0	0	0	999	999
20/10/2024 14:30	1	70.2	1	0	0	1	0	999	999
20/10/2024 14:45	1	78.9	1	0	0	0	0	999	999
20/10/2024 15:00	1	153.3	1	0	0	0	0	999	999
20/10/2024 15:15	1	22.8	1	0	0	0	0	999	999
20/10/2024 15:30	1	107.1	1	0	0	0	0	999	999
20/10/2024 15:45	1	70.6	1	0	0	0	0	999	999
20/10/2024 16:00	1	109.1	1	0	0	0	0	999	999
20/10/2024 16:15	1	96.5	1	0	0	0	0	999	999
20/10/2024 16:30	1	192	1	0	0	0	0	999	999
20/10/2024 16:45	1	174.3	1	0	0	0	1	999	999
20/10/2024 17:00	0.003906	54.3	1	0	0	0	0	999	999
20/10/2024 17:15	0.003906	139.8	1	0	0	0	0	999	999
20/10/2024 17:30	0.003906	25.3	1	0	0	0	0	999	999
20/10/2024 17:45	0.003906	101.1	1	0	0	0	0	999	999
20/10/2024 18:00	0.003906	65.4	1	0	0	0	0	999	999
20/10/2024 18:15	0.003906	74.1	1	0	0	0	0	999	999
20/10/2024 18:30	0.003906	131.7	1	0	0	0	0	999	999
20/10/2024 18:45	0.003906	59.6	1	0	0	0	0	999	999
20/10/2024 19:00	0.003906	72.3	1	0	0	0	0	999	999

20/10/2024 19:15	0.003906	143.9	1	0	0	0	0	999	999
20/10/2024 19:30	0.003906	66.9	1	0	0	0	0	999	999
20/10/2024 19:45	0.035156	90.8	1	0	0	0	0	999	999
20/10/2024 20:00	0.144531	137.5	1	0	0	0	0	999	999
20/10/2024 20:15	0.363281	99	1	0	0	0	0	999	999
20/10/2024 20:30	0.636719	119.8	1	0	0	0	0	999	999
20/10/2024 20:45	0.855469	87.6	1	0	0	0	0	999	999
20/10/2024 21:00	0.964844	84.2	1	0	0	0	0	999	999
20/10/2024 21:15	0.996094	107.7	1	0	0	0	0	999	999
20/10/2024 21:30	1	52.8	1	0	0	1	0	999	999
20/10/2024 21:45	1	338	1	0	0	0	0	999	999
20/10/2024 22:00	1	87.2	1	0	0	0	0	999	999
20/10/2024 22:15	1	82.8	1	0	0	0	0	999	999
20/10/2024 22:30	1	6.8	1	0	0	0	0	999	999
20/10/2024 22:45	1	55.1	1	0	0	0	0	999	999
20/10/2024 23:00	1	61.5	1	0	0	0	0	999	999
20/10/2024 23:15	1	189.3	1	0	0	0	0	999	999
20/10/2024 23:30	1	113.8	1	0	0	0	0	999	999
20/10/2024 23:45	1	69.3	1	0	0	0	1	999	999
21/10/2024 00:00	0.003906	57	1	0	0	0	0	999	999
21/10/2024 00:15	0.003906	51.2	1	0	0	0	0	999	999
21/10/2024 00:30	0.035156	104.6	1	0	0	0	0	999	999
21/10/2024 00:45	0.144531	49.8	1	0	0	0	0	999	999
21/10/2024 01:00	0.363281	107.3	1	0	0	0	0	999	999
21/10/2024 01:15	0.636719	119.6	1	0	1	0	0	999	TRUE
21/10/2024 01:30	0.855469	193.6	0	0	0	0	0	999	999
21/10/2024 01:45	0.964844	13	0	0	0	0	0	999	999
21/10/2024 02:00	0.996094	19.5	0	0	0	0	0	999	999
21/10/2024 02:15	1	21	0	0	0	1	0	999	999
21/10/2024 02:30	1	22.5	0	0	0	0	0	999	999
21/10/2024 02:45	1	21	0	0	0	0	0	999	999
21/10/2024 03:00	1	22.5	0	0	0	0	0	999	999
21/10/2024 03:15	1	24.5	0	0	0	0	0	999	999
21/10/2024 03:30	1	6.8	0	0	0	0	0	999	999
21/10/2024 03:45	1	9.4	0	0	0	0	0	999	999
21/10/2024 04:00	1	28.6	0	0	0	0	0	999	999

21/10/2024 04:15	1	21.6	0	0	0	0	0	999	999
21/10/2024 04:30	1	42.2	0	0	0	0	1	999	999
21/10/2024 04:45	0.035156	23	0	0	0	0	0	999	999
21/10/2024 05:00	0.144531	24.8	0	0	0	0	0	999	999
21/10/2024 05:15	0.363281	13.3	0	0	0	0	0	999	999
21/10/2024 05:30	0.636719	28.1	0	0	0	0	0	999	999
21/10/2024 05:45	0.855469	17	0	0	0	0	0	999	999
21/10/2024 06:00	0.964844	23.5	0	0	0	0	0	999	999
21/10/2024 06:15	0.996094	19.5	0	0	0	0	0	999	999
21/10/2024 06:30	1	26.4	0	0	0	1	0	999	999
21/10/2024 06:45	1	24.4	0	0	0	0	0	999	999
21/10/2024 07:00	1	20	0	0	0	0	0	999	999
21/10/2024 07:15	1	19.5	0	0	0	0	0	999	999
21/10/2024 07:30	1	70.2	0	0	0	0	0	999	999
21/10/2024 07:45	1	35.8	0	0	0	0	0	999	999
21/10/2024 08:00	1	48.7	0	0	0	0	0	999	999
21/10/2024 08:15	1	406.6	1	1	0	0	0	999	999
21/10/2024 08:30	1	37	1	0	0	0	0	999	999
21/10/2024 08:45	1	169.6	1	0	0	0	1	999	999
21/10/2024 09:00	0.003906	131.5	1	0	0	0	0	999	999
21/10/2024 09:15	0.003906	269.7	1	0	0	0	0	999	999
21/10/2024 09:30	0.035156	86.7	1	0	0	0	0	999	999
21/10/2024 09:45	0.144531	185.1	1	0	0	0	0	999	999
21/10/2024 10:00	0.363281	174.4	1	0	0	0	0	999	999
21/10/2024 10:15	0.636719	99	1	0	0	0	0	999	999
21/10/2024 10:30	0.855469	140.1	1	0	0	0	0	999	999
21/10/2024 10:45	0.964844	186.2	1	0	0	0	0	999	999
21/10/2024 11:00	0.996094	133.1	1	0	0	0	0	999	999
21/10/2024 11:15	1	313.4	1	0	0	1	0	999	999
21/10/2024 11:30	1	128.7	1	0	0	0	0	999	999
21/10/2024 11:45	1	138.4	1	0	0	0	0	999	999
21/10/2024 12:00	1	175	1	0	0	0	0	999	999
21/10/2024 12:15	1	175.7	1	0	0	0	0	999	999
21/10/2024 12:30	1	351.2	1	0	0	0	0	999	999
21/10/2024 12:45	1	134.9	1	0	0	0	0	999	999
21/10/2024 13:00	1	477.4	1	0	0	0	0	999	999

21/10/2024 13:15	1	52.3	1	0	0	0	0	999	999
21/10/2024 13:30	1	138.7	1	0	0	0	1	999	999
21/10/2024 13:45	0.035156	202.9	1	0	0	0	0	999	999
21/10/2024 14:00	0.144531	296.5	1	0	0	0	0	999	999
21/10/2024 14:15	0.363281	130.6	1	0	0	0	0	999	999
21/10/2024 14:30	0.636719	70.6	1	0	0	0	0	999	999
21/10/2024 14:45	0.855469	371.7	1	0	0	0	0	999	999
21/10/2024 15:00	0.964844	404.7	1	0	0	0	0	999	999
21/10/2024 15:15	0.996094	316.8	1	0	0	0	0	999	999
21/10/2024 15:30	1	181.1	1	0	0	1	0	999	999
21/10/2024 15:45	1	149.5	1	0	0	0	0	999	999
21/10/2024 16:00	1	188.9	1	0	0	0	0	999	999
21/10/2024 16:15	1	116.1	1	0	0	0	0	999	999
21/10/2024 16:30	1	75.6	1	0	0	0	0	999	999
21/10/2024 16:45	1	116.5	1	0	0	0	0	999	999
21/10/2024 17:00	1	124.7	1	0	0	0	0	999	999
21/10/2024 17:15	1	113.7	1	0	1	0	1	999	TRUE
22/10/2024 18:00	0	11.9	0	0	0	0	0	999	999
22/10/2024 18:15	0.996094	11.4	0	0	0	0	0	999	999
22/10/2024 18:30	0.035156	11.5	0	0	0	0	0	999	999
22/10/2024 18:45	0.144531	11.5	0	0	0	0	0	999	999
22/10/2024 19:00	0.363281	11.7	0	0	0	0	0	999	999
22/10/2024 19:15	0.636719	11.8	0	0	0	0	0	999	999
22/10/2024 19:30	0.855469	12	0	0	0	0	0	999	999
22/10/2024 19:45	0.964844	12	0	0	0	0	0	999	999
22/10/2024 20:00	0.996094	11.9	0	0	0	0	0	999	999
22/10/2024 20:15	1	12.1	0	0	0	1	0	999	999
22/10/2024 20:30	1	11.7	0	0	0	0	0	999	999
22/10/2024 20:45	1	12.3	0	0	0	0	0	999	999
22/10/2024 21:00	1	12	0	0	0	0	0	999	999
22/10/2024 21:15	1	12	0	0	0	0	0	999	999
22/10/2024 21:30	1	11.7	0	0	0	0	0	999	999
22/10/2024 21:45	1	11.8	0	0	0	0	0	999	999
22/10/2024 22:00	1	11.5	0	0	0	0	0	999	999
22/10/2024 22:15	1	11.5	0	0	0	0	0	999	999
22/10/2024 22:30	1	12.5	0	0	0	0	1	999	999

22/10/2024 22:45	0.035156	11.5	0	0	0	0	0	999	999
22/10/2024 23:00	0.035156	11.4	0	0	0	0	0	999	999
22/10/2024 23:15	0.035156	12.1	0	0	0	0	0	999	999
22/10/2024 23:30	0.144531	11.2	0	0	0	0	0	999	999
22/10/2024 23:45	0.363281	11	0	0	0	0	0	999	999
23/10/2024 00:00	0.636719	11.1	0	0	0	0	0	999	999
23/10/2024 00:15	0.855469	11.2	0	0	0	0	0	999	999
23/10/2024 00:30	0.964844	10.8	0	0	0	0	0	999	999
23/10/2024 00:45	0.964844	10.8	0	0	0	0	0	999	999
23/10/2024 01:00	0.996094	10.1	0	0	0	0	0	999	999
23/10/2024 01:15	1	10.4	0	0	0	1	0	999	999
23/10/2024 01:30	1	10.6	0	0	0	0	0	999	999
23/10/2024 01:45	1	10.4	0	0	0	0	0	999	999
23/10/2024 02:00	1	10.4	0	0	0	0	0	999	999
23/10/2024 02:15	1	10.1	0	0	0	0	0	999	999
23/10/2024 02:30	1	9.9	0	0	0	0	0	999	999
23/10/2024 02:45	1	10	0	0	0	0	0	999	999
23/10/2024 03:00	1	9.7	0	0	0	0	0	999	999
23/10/2024 03:15	1	10.1	0	0	0	0	1	999	999
23/10/2024 03:30	0.003906	9.8	0	0	0	0	0	999	999
23/10/2024 03:45	0.035156	9.3	0	0	0	0	0	999	999
23/10/2024 04:00	0.144531	9.2	0	0	0	0	0	999	999
23/10/2024 04:15	0.363281	9.6	0	0	0	0	0	999	999
23/10/2024 04:30	0.636719	8.9	0	0	0	0	0	999	999
23/10/2024 04:45	0.855469	9.3	0	0	0	0	0	999	999
23/10/2024 05:00	0.964844	9	0	0	0	0	0	999	999
23/10/2024 05:15	0.996094	9.1	0	0	0	0	0	999	999
23/10/2024 05:30	1	8.9	0	0	0	1	0	999	999
23/10/2024 05:45	1	8.9	0	0	0	0	0	999	999
23/10/2024 06:00	1	8.9	0	0	0	0	0	999	999
23/10/2024 06:15	1	8.6	0	0	0	0	0	999	999
23/10/2024 06:30	1	8.6	0	0	0	0	0	999	999
23/10/2024 06:45	1	8.8	0	0	0	0	0	999	999
23/10/2024 07:00	1	8.6	0	0	0	0	0	999	999
23/10/2024 07:15	1	8.4	0	0	0	0	0	999	999
23/10/2024 07:30	1	8.5	0	0	0	0	0	999	999

23/10/2024 07:45	1	8.5	0	0	0	0	1	999	999
23/10/2024 08:00	0.035156	8.3	0	0	0	0	0	999	999
23/10/2024 08:15	0.144531	8.4	0	0	0	0	0	999	999
23/10/2024 08:30	0.363281	8.4	0	0	0	0	0	999	999
23/10/2024 08:45	0.636719	8.3	0	0	0	0	0	999	999
23/10/2024 09:00	0.855469	8.3	0	0	0	0	0	999	999
23/10/2024 09:15	0.964844	8.2	0	0	0	0	0	999	999
23/10/2024 09:30	0.996094	8.2	0	0	0	0	0	999	999
23/10/2024 09:45	1	8.1	0	0	0	1	0	999	999
23/10/2024 10:00	1	8.1	0	0	0	0	0	999	999
23/10/2024 10:15	1	7.9	0	0	0	0	0	999	999
23/10/2024 10:30	1	8.1	0	0	0	0	0	999	999
23/10/2024 10:45	1	8.1	0	0	0	0	0	999	999
23/10/2024 11:00	1	7.8	0	0	0	0	0	999	999
23/10/2024 11:15	1	7.6	0	0	0	0	0	999	999
23/10/2024 11:30	1	7.6	0	0	0	0	0	999	999
23/10/2024 11:45	1	7.8	0	0	0	0	0	999	999
23/10/2024 12:00	1	7.9	0	0	0	0	1	999	999
23/10/2024 12:15	0.035156	7.8	0	0	0	0	0	999	999
23/10/2024 12:30	0.144531	8.3	0	0	0	0	0	999	999
23/10/2024 12:45	0.363281	8.1	0	0	0	0	0	999	999
23/10/2024 13:00	0.636719	7.9	0	0	0	0	0	999	999
23/10/2024 13:15	0.855469	7.9	0	0	0	0	0	999	999
23/10/2024 13:30	0.964844	7.8	0	0	0	0	0	999	999
23/10/2024 13:45	0.996094	8	0	0	0	0	0	999	999
23/10/2024 14:00	1	7.8	0	0	0	1	0	999	999
23/10/2024 14:15	1	7.9	0	0	0	0	0	999	999
23/10/2024 14:30	1	7.9	0	0	0	0	0	999	999
23/10/2024 14:45	1	7.5	0	0	0	0	0	999	999
23/10/2024 15:00	1	7.3	0	0	0	0	0	999	999
23/10/2024 15:15	1	7.5	0	0	0	0	0	999	999
23/10/2024 15:30	1	7.3	0	0	0	0	0	999	999
23/10/2024 15:45	1	7.3	0	0	0	0	0	999	999
23/10/2024 16:00	1	7.5	0	0	0	0	0	999	999
23/10/2024 16:15	1	7.6	0	0	0	0	1	999	999
23/10/2024 16:30	0.035156	7.6	0	0	0	0	0	999	999

23/10/2024 16:45	0.144531	7.6	0	0	0	0	0	999	999
23/10/2024 17:00	0.363281	7.3	0	0	0	0	0	999	999
23/10/2024 17:15	0.636719	7.4	0	0	0	0	0	999	999
23/10/2024 17:30	0.855469	7.5	0	0	0	0	0	999	999
23/10/2024 17:45	0.964844	7.4	0	0	0	0	0	999	999
23/10/2024 18:00	0.996094	7.3	0	0	0	0	0	999	999
23/10/2024 18:15	1	7.3	0	0	0	1	0	999	999
23/10/2024 18:30	1	7	0	0	0	0	0	999	999
23/10/2024 18:45	1	7.2	0	0	0	0	0	999	999
23/10/2024 19:00	1	7.2	0	0	0	0	0	999	999
23/10/2024 19:15	1	7.1	0	0	0	0	0	999	999
23/10/2024 19:30	1	6.7	0	0	0	0	0	999	999
23/10/2024 19:45	1	7	0	0	0	0	0	999	999
23/10/2024 20:00	1	7.3	0	0	0	0	0	999	999
23/10/2024 20:15	1	7.3	0	0	0	0	0	999	999
23/10/2024 20:30	1	6.9	0	0	0	0	1	999	999
23/10/2024 20:45	0.035156	7.2	0	0	0	0	0	999	999
23/10/2024 21:00	0.144531	7.2	0	0	0	0	0	999	999
23/10/2024 21:15	0.363281	7.1	0	0	0	0	0	999	999
23/10/2024 21:30	0.636719	7.2	0	0	0	0	0	999	999
23/10/2024 21:45	0.855469	7	0	0	0	0	0	999	999
23/10/2024 22:00	0.964844	6.7	0	0	0	0	0	999	999
23/10/2024 22:15	0.996094	6.7	0	0	0	0	0	999	999
23/10/2024 22:30	1	7.1	0	0	0	1	0	999	999
23/10/2024 22:45	1	7	0	0	0	0	0	999	999
23/10/2024 23:00	1	7	0	0	0	0	0	999	999
23/10/2024 23:15	1	6.8	0	0	0	0	0	999	999
23/10/2024 23:30	1	6.8	0	0	0	0	0	999	999
23/10/2024 23:45	1	6.9	0	0	0	0	0	999	999
24/10/2024 00:00	1	6.8	0	0	0	0	0	999	999
24/10/2024 00:15	1	6.8	0	0	0	0	0	999	999
24/10/2024 00:30	1	6.4	0	0	0	0	0	999	999
24/10/2024 00:45	1	6.9	0	0	0	0	1	999	999
24/10/2024 01:00	0.003906	6.9	0	0	0	0	0	999	999
24/10/2024 01:15	0.003906	6.9	0	0	0	0	0	999	999
24/10/2024 01:30	0.003906	6.5	0	0	0	0	0	999	999

24/10/2024 01:45	0.003906	6.4	0	0	0	0	0	999	999
24/10/2024 02:00	0.003906	6.3	0	0	0	0	0	999	999
24/10/2024 02:15	0.035156	6.7	0	0	0	0	0	999	999
24/10/2024 02:30	0.035156	6.3	0	0	0	0	0	999	999
24/10/2024 02:45	0.144531	6.3	0	0	0	0	0	999	999
24/10/2024 03:00	0.363281	6.6	0	0	0	0	0	999	999
24/10/2024 03:15	0.636719	6.3	0	0	0	0	0	999	999
24/10/2024 03:30	0.855469	6.4	0	0	0	0	0	999	999
24/10/2024 03:45	0.964844	8	0	0	0	0	0	999	999
24/10/2024 04:00	0.996094	6.3	0	0	0	0	0	999	999
24/10/2024 04:15	0.996094	6.2	0	0	0	0	0	999	999
24/10/2024 04:30	1	6.2	0	0	0	1	0	999	999
24/10/2024 04:45	1	6.1	0	0	0	0	0	999	999
24/10/2024 05:00	1	6.2	0	0	0	0	0	999	999
24/10/2024 05:15	1	6.3	0	0	0	0	0	999	999
24/10/2024 05:30	1	6.1	0	0	0	0	0	999	999
24/10/2024 05:45	1	6.3	0	0	0	0	0	999	999
24/10/2024 06:00	1	6.5	0	0	0	0	0	999	999
24/10/2024 06:15	1	6.9	0	0	0	0	0	999	999
24/10/2024 06:30	1	6.4	0	0	0	0	1	999	999
24/10/2024 06:45	0.003906	6.2	0	0	0	0	0	999	999
24/10/2024 07:00	0.035156	6.4	0	0	0	0	0	999	999
24/10/2024 07:15	0.144531	6.5	0	0	0	0	0	999	999
24/10/2024 07:30	0.363281	6.2	0	0	0	0	0	999	999
24/10/2024 07:45	0.636719	6.4	0	0	0	0	0	999	999
24/10/2024 08:00	0.855469	6.5	0	0	0	0	0	999	999
24/10/2024 08:15	0.964844	6.5	0	0	0	0	0	999	999
24/10/2024 08:30	0.996094	6.3	0	0	0	0	0	999	999
24/10/2024 08:45	1	6.3	0	0	0	1	0	999	999
24/10/2024 09:00	1	6.3	0	0	0	0	0	999	999
24/10/2024 09:15	1	6.2	0	0	0	0	0	999	999
24/10/2024 09:30	1	6.2	0	0	0	0	0	999	999
24/10/2024 09:45	1	6.2	0	0	0	0	0	999	999
24/10/2024 10:00	1	5.5	0	0	0	0	0	999	999
24/10/2024 10:15	1	5.9	0	0	0	0	0	999	999
24/10/2024 10:30	1	5.9	0	0	0	0	0	999	999

24/10/2024 10:45	1	6.1	0	0	0	0	0	999	999
24/10/2024 11:00	1	8.6	0	0	0	0	1	999	999
24/10/2024 11:15	0.035156	6.1	0	0	0	0	0	999	999
24/10/2024 11:30	0.144531	5.8	0	0	0	0	0	999	999
24/10/2024 11:45	0.363281	5.7	0	0	0	0	0	999	999
24/10/2024 12:00	0.636719	5.6	0	0	0	0	0	999	999
24/10/2024 12:15	0.855469	5.5	0	0	0	0	0	999	999
24/10/2024 12:30	0.964844	5.9	0	0	0	0	0	999	999
24/10/2024 12:45	0.996094	5.7	0	0	0	0	0	999	999
24/10/2024 13:00	1	5.9	0	0	0	1	0	999	999
24/10/2024 13:15	1	5.5	0	0	0	0	0	999	999
24/10/2024 13:30	1	5.7	0	0	0	0	0	999	999
24/10/2024 13:45	1	5.6	0	0	0	0	0	999	999
24/10/2024 14:00	1	5.4	0	0	0	0	0	999	999
24/10/2024 14:15	1	5.3	0	0	0	0	0	999	999
24/10/2024 14:30	1	5.6	0	0	0	0	0	999	999
24/10/2024 14:45	1	5.3	0	0	0	0	0	999	999
24/10/2024 15:00	1	5.2	0	0	0	0	0	999	999
24/10/2024 15:15	1	5.4	0	0	0	0	1	999	999
24/10/2024 15:30	0.003906	5.4	0	0	0	0	0	999	999
24/10/2024 15:45	0.003906	5.4	0	0	0	0	0	999	999
24/10/2024 16:00	0.003906	5.8	0	0	0	0	0	999	999
24/10/2024 16:15	0.003906	5.3	0	0	0	0	0	999	999
24/10/2024 16:30	0.003906	5.6	0	0	0	0	0	999	999
24/10/2024 16:45	0.003906	5.3	0	0	0	0	0	999	999
24/10/2024 17:00	0.003906	5.2	0	0	0	0	0	999	999
24/10/2024 17:15	0.003906	5.3	0	0	0	0	0	999	999
24/10/2024 17:30	0.003906	5.1	0	0	0	0	0	999	999
24/10/2024 17:45	0.035156	5.3	0	0	0	0	0	999	999
24/10/2024 18:00	0.144531	6.4	0	0	0	0	0	999	999
24/10/2024 18:15	0.363281	5.2	0	0	0	0	0	999	999
24/10/2024 18:30	0.636719	6.3	0	0	0	0	0	999	999
24/10/2024 18:45	0.855469	5.4	0	0	0	0	0	999	999
24/10/2024 19:00	0.855469	5.3	0	0	0	0	0	999	999
24/10/2024 19:15	0.855469	5.3	0	0	0	0	0	999	999
24/10/2024 19:30	0.855469	5.4	0	0	0	0	0	999	999

24/10/2024 19:45	0.636719	7.4	0	0	0	0	0	999	999
24/10/2024 20:00	0.636719	5.4	0	0	0	0	0	999	999
24/10/2024 20:15	0.363281	5.9	0	0	0	0	0	999	999
24/10/2024 20:30	0.363281	5.3	0	0	0	0	0	999	999
24/10/2024 20:45	0.363281	5.3	0	0	0	0	0	999	999
24/10/2024 21:00	0.636719	5.4	0	0	0	0	0	999	999
24/10/2024 21:15	0.855469	5.4	0	0	0	0	0	999	999
24/10/2024 21:30	0.964844	5.6	0	0	0	0	0	999	999
24/10/2024 21:45	0.996094	5.4	0	0	0	0	0	999	999
24/10/2024 22:00	0.996094	6.6	0	0	0	0	0	999	999
24/10/2024 22:15	1	5.9	0	0	0	1	0	999	999
24/10/2024 22:30	1	5.5	0	0	0	0	0	999	999
24/10/2024 22:45	1	5.7	0	0	0	0	0	999	999
24/10/2024 23:00	1	6.4	0	0	0	0	0	999	999
24/10/2024 23:15	1	5.4	0	0	0	0	0	999	999
24/10/2024 23:30	1	5.4	0	0	0	0	0	999	999
24/10/2024 23:45	1	7.4	0	0	0	0	0	999	999
25/10/2024 00:00	1	5.8	0	0	0	0	0	999	999
25/10/2024 00:15	1	6.1	0	0	0	0	1	999	999
25/10/2024 00:30	0.003906	6.4	0	0	0	0	0	999	999
25/10/2024 00:45	0.003906	6.2	0	0	0	0	0	999	999
25/10/2024 01:00	0.003906	7.3	0	0	0	0	0	999	999
25/10/2024 01:15	0.035156	6.7	0	0	0	0	0	999	999
25/10/2024 01:30	0.144531	9.8	0	0	0	0	0	999	999
25/10/2024 01:45	0.363281	6.6	0	0	0	0	0	999	999
25/10/2024 02:00	0.636719	6.5	0	0	0	0	0	999	999
25/10/2024 02:15	0.855469	7.1	0	0	0	0	0	999	999
25/10/2024 02:30	0.964844	12.7	0	0	0	0	0	999	999
25/10/2024 02:45	0.996094	6.8	0	0	0	0	0	999	999
25/10/2024 03:00	1	7.3	0	0	0	1	0	999	999
25/10/2024 03:15	1	7.4	0	0	0	0	0	999	999
25/10/2024 03:30	1	7.4	0	0	0	0	0	999	999
25/10/2024 03:45	1	8.7	0	0	0	0	0	999	999
25/10/2024 04:00	1	7.7	0	0	0	0	0	999	999
25/10/2024 04:15	1	8.1	0	0	0	0	0	999	999
25/10/2024 04:30	1	8.1	0	0	0	0	0	999	999

25/10/2024 04:45	1	9.1	0	0	0	0	0	999	999
25/10/2024 05:00	1	9.8	0	0	0	0	0	999	999
25/10/2024 05:15	1	9.3	0	0	0	0	1	999	999
25/10/2024 05:30	0.035156	9.8	0	0	0	0	0	999	999
25/10/2024 05:45	0.144531	9.1	0	0	0	0	0	999	999
25/10/2024 06:00	0.363281	10.8	0	0	0	0	0	999	999
25/10/2024 06:15	0.636719	11.9	0	0	0	0	0	999	999
25/10/2024 06:30	0.855469	12.5	0	0	0	0	0	999	999
25/10/2024 06:45	0.964844	9.4	0	0	0	0	0	999	999
25/10/2024 07:00	0.996094	10.8	0	0	0	0	0	999	999
25/10/2024 07:15	1	10.3	0	0	0	1	0	999	999
25/10/2024 07:30	1	14.1	0	0	0	0	0	999	999
25/10/2024 07:45	1	9.4	0	0	0	0	0	999	999
25/10/2024 08:00	1	12.6	0	0	0	0	0	999	999
25/10/2024 08:15	1	10.1	0	0	0	0	0	999	999
25/10/2024 08:30	1	9.1	0	0	0	0	0	999	999
25/10/2024 08:45	1	18.4	0	0	0	0	0	999	999
25/10/2024 09:00	1	8.7	0	0	0	0	0	999	999
25/10/2024 09:15	1	13.6	0	0	0	0	0	999	999
25/10/2024 09:30	1	13.1	0	0	0	0	1	999	999
25/10/2024 09:45	0.035156	12	0	0	0	0	0	999	999
25/10/2024 10:00	0.035156	11.4	0	0	0	0	0	999	999
25/10/2024 10:15	0.035156	9.7	0	0	0	0	0	999	999
25/10/2024 10:30	0.144531	7.5	0	0	0	0	0	999	999
25/10/2024 10:45	0.363281	14.3	0	0	0	0	0	999	999
25/10/2024 11:00	0.636719	18.1	0	0	0	0	0	999	999
25/10/2024 11:15	0.855469	7.3	0	0	0	0	0	999	999
25/10/2024 11:30	0.964844	7.3	0	0	0	0	0	999	999
25/10/2024 11:45	0.964844	7.6	0	0	0	0	0	999	999
25/10/2024 12:00	0.996094	12.5	0	0	0	0	0	999	999
25/10/2024 12:15	1	7.3	0	0	0	1	0	999	999
25/10/2024 12:30	1	7.2	0	0	0	0	0	999	999
25/10/2024 12:45	1	7.5	0	0	0	0	0	999	999
25/10/2024 13:00	1	7.2	0	0	0	0	0	999	999
25/10/2024 13:15	1	7.3	0	0	0	0	0	999	999
25/10/2024 13:30	1	7.3	0	0	0	0	0	999	999

25/10/2024 13:45	1	7	0	0	0	0	0	999	999
25/10/2024 14:00	1	7	0	0	0	0	0	999	999
25/10/2024 14:15	1	6.8	0	0	0	0	1	999	999
25/10/2024 14:30	0.035156	6.8	0	0	0	0	0	999	999
25/10/2024 14:45	0.144531	7.6	0	0	0	0	0	999	999
25/10/2024 15:00	0.363281	7.2	0	0	0	0	0	999	999
25/10/2024 15:15	0.636719	7.3	0	0	0	0	0	999	999
25/10/2024 15:30	0.855469	7.2	0	0	0	0	0	999	999
25/10/2024 15:45	0.964844	9.2	0	0	0	0	0	999	999
25/10/2024 16:00	0.996094	11.4	0	0	0	0	0	999	999
25/10/2024 16:15	1	8.9	0	0	0	1	0	999	999
25/10/2024 16:30	1	7.8	0	0	0	0	0	999	999
25/10/2024 16:45	1	6.5	0	0	0	0	0	999	999
25/10/2024 17:00	1	6.7	0	0	0	0	0	999	999
25/10/2024 17:15	1	6.7	0	0	0	0	0	999	999
25/10/2024 17:30	1	6.2	0	0	0	0	0	999	999
25/10/2024 17:45	1	6.6	0	0	0	0	0	999	999
25/10/2024 18:00	1	6.2	0	0	0	0	0	999	999
25/10/2024 18:15	1	6.2	0	0	0	0	0	999	999
25/10/2024 18:30	1	6.8	0	0	0	0	1	999	999
25/10/2024 18:45	0.035156	7.2	0	0	0	0	0	999	999
25/10/2024 19:00	0.144531	6.4	0	0	0	0	0	999	999
25/10/2024 19:15	0.363281	6	0	0	0	0	0	999	999
25/10/2024 19:30	0.636719	6.3	0	0	0	0	0	999	999
25/10/2024 19:45	0.855469	6.3	0	0	0	0	0	999	999
25/10/2024 20:00	0.964844	6.1	0	0	0	0	0	999	999
25/10/2024 20:15	0.996094	5.9	0	0	0	0	0	999	999
25/10/2024 20:30	1	6.2	0	0	0	1	0	999	999
25/10/2024 20:45	1	6.2	0	0	0	0	0	999	999
25/10/2024 21:00	1	6.4	0	0	0	0	0	999	999
25/10/2024 21:15	1	6	0	0	0	0	0	999	999
25/10/2024 21:30	1	6.4	0	0	0	0	0	999	999
25/10/2024 21:45	1	6.3	0	0	0	0	0	999	999
25/10/2024 22:00	1	6.3	0	0	0	0	0	999	999
25/10/2024 22:15	1	6.2	0	0	0	0	0	999	999
25/10/2024 22:30	1	6.4	0	0	0	0	0	999	999

25/10/2024 22:45	1	6.4	0	0	0	0	1	999	999
25/10/2024 23:00	0.035156	6.3	0	0	0	0	0	999	999
25/10/2024 23:15	0.144531	6.2	0	0	0	0	0	999	999
25/10/2024 23:30	0.363281	6.4	0	0	0	0	0	999	999
25/10/2024 23:45	0.636719	6.2	0	0	0	0	0	999	999
26/10/2024 00:00	0.855469	6.1	0	0	0	0	0	999	999
26/10/2024 00:15	0.964844	6.5	0	0	0	0	0	999	999
26/10/2024 00:30	0.996094	6.1	0	0	0	0	0	999	999
26/10/2024 00:45	1	6.1	0	0	0	1	0	999	999
26/10/2024 01:00	1	6.2	0	0	0	0	0	999	999
26/10/2024 01:15	1	6.1	0	0	0	0	0	999	999
26/10/2024 01:30	1	6.4	0	0	0	0	0	999	999
26/10/2024 01:45	1	6.4	0	0	0	0	0	999	999
26/10/2024 02:00	1	6.2	0	0	0	0	0	999	999
26/10/2024 02:15	1	6.5	0	0	0	0	0	999	999
26/10/2024 02:30	1	6.7	0	0	0	0	0	999	999
26/10/2024 02:45	1	6.1	0	0	0	0	0	999	999
26/10/2024 03:00	1	6.4	0	0	0	0	1	999	999
26/10/2024 03:15	0.003906	6.2	0	0	0	0	0	999	999
26/10/2024 03:30	0.003906	6.1	0	0	0	0	0	999	999
26/10/2024 03:45	0.003906	6.3	0	0	0	0	0	999	999
26/10/2024 04:00	0.003906	5.9	0	0	0	0	0	999	999
26/10/2024 04:15	0.003906	6.1	0	0	0	0	0	999	999
26/10/2024 04:30	0.003906	6.4	0	0	0	0	0	999	999
26/10/2024 04:45	0.035156	6	0	0	0	0	0	999	999
26/10/2024 05:00	0.144531	6.1	0	0	0	0	0	999	999
26/10/2024 05:15	0.363281	6.1	0	0	0	0	0	999	999
26/10/2024 05:30	0.636719	6.4	0	0	0	0	0	999	999
26/10/2024 05:45	0.855469	6.1	0	0	0	0	0	999	999
26/10/2024 06:00	0.964844	6.2	0	0	0	0	0	999	999
26/10/2024 06:15	0.996094	6.7	0	0	0	0	0	999	999
26/10/2024 06:30	1	6.1	0	0	0	1	0	999	999
26/10/2024 06:45	1	6.3	0	0	0	0	0	999	999
26/10/2024 07:00	1	6.4	0	0	0	0	0	999	999
26/10/2024 07:15	1	6.2	0	0	0	0	0	999	999
26/10/2024 07:30	1	6.3	0	0	0	0	0	999	999

26/10/2024 07:45	1	6.1	0	0	0	0	0	999	999
26/10/2024 08:00	1	6.5	0	0	0	0	0	999	999
26/10/2024 08:15	1	6.6	0	0	0	0	0	999	999
26/10/2024 08:30	1	6.3	0	0	0	0	0	999	999
26/10/2024 08:45	1	6.4	0	0	0	0	1	999	999
26/10/2024 09:00	0.003906	6.9	0	0	0	0	0	999	999
26/10/2024 09:15	0.035156	6.7	0	0	0	0	0	999	999
26/10/2024 09:30	0.144531	6.3	0	0	0	0	0	999	999
26/10/2024 09:45	0.363281	6.5	0	0	0	0	0	999	999
26/10/2024 10:00	0.636719	6.7	0	0	0	0	0	999	999
26/10/2024 10:15	0.855469	6.2	0	0	0	0	0	999	999
26/10/2024 10:30	0.964844	6.4	0	0	0	0	0	999	999
26/10/2024 10:45	0.996094	6.2	0	0	0	0	0	999	999
26/10/2024 11:00	1	6.2	0	0	0	1	0	999	999
26/10/2024 11:15	1	6.1	0	0	0	0	0	999	999
26/10/2024 11:30	1	6.2	0	0	0	0	1	999	999
26/10/2024 11:45	0.996094	5.9	0	0	0	0	0	999	999
26/10/2024 12:00	0.996094	6.2	0	0	0	0	0	999	999
26/10/2024 12:15	0.964844	6.2	0	0	0	0	0	999	999
26/10/2024 12:30	0.855469	6.2	0	0	0	0	0	999	999
26/10/2024 12:45	0.636719	6	0	0	0	0	0	999	999
26/10/2024 13:00	0.636719	6.2	0	0	0	0	0	999	999
26/10/2024 13:15	0.363281	6.6	0	0	0	0	0	999	999
26/10/2024 13:30	0.363281	6.5	0	0	0	0	0	999	999
26/10/2024 13:45	0.363281	6.2	0	0	0	0	0	999	999
26/10/2024 14:00	0.144531	6.5	0	0	0	0	0	999	999
26/10/2024 14:15	0.363281	6.2	0	0	0	0	0	999	999
26/10/2024 14:30	0.636719	6.5	0	0	0	0	0	999	999
26/10/2024 14:45	0.855469	6.7	0	0	0	0	0	999	999
26/10/2024 15:00	0.855469	6.6	0	0	0	0	0	999	999
26/10/2024 15:15	0.964844	6.8	0	0	0	0	0	999	999
26/10/2024 15:30	0.964844	6.4	0	0	0	0	0	999	999
26/10/2024 15:45	0.996094	6.7	0	0	0	0	0	999	999
26/10/2024 16:00	1	7.2	0	0	0	1	0	999	999
26/10/2024 16:15	1	6.8	0	0	0	0	0	999	999
26/10/2024 16:30	1	7.4	0	0	0	0	0	999	999

26/10/2024 16:45	1	6.8	0	0	0	0	0	999	999
26/10/2024 17:00	1	7.2	0	0	0	0	0	999	999
26/10/2024 17:15	1	7.4	0	0	0	0	0	999	999
26/10/2024 17:30	1	6.8	0	0	0	0	0	999	999
26/10/2024 17:45	1	7	0	0	0	0	0	999	999
26/10/2024 18:00	1	6.8	0	0	0	0	1	999	999
26/10/2024 18:15	0.003906	7.2	0	0	0	0	0	999	999
26/10/2024 18:30	0.035156	7.2	0	0	0	0	0	999	999
26/10/2024 18:45	0.144531	7.7	0	0	0	0	0	999	999
26/10/2024 19:00	0.363281	7.4	0	0	0	0	0	999	999
26/10/2024 19:15	0.636719	7.6	0	0	0	0	0	999	999
26/10/2024 19:30	0.855469	7.6	0	0	0	0	0	999	999
26/10/2024 19:45	0.964844	7.6	0	0	0	0	0	999	999
26/10/2024 20:00	0.996094	7.8	0	0	0	0	0	999	999
26/10/2024 20:15	1	7.6	0	0	0	1	0	999	999
26/10/2024 20:30	1	8.2	0	0	0	0	0	999	999
26/10/2024 20:45	1	8.6	0	0	0	0	0	999	999
26/10/2024 21:00	1	8.6	0	0	0	0	0	999	999
26/10/2024 21:15	1	8.3	0	0	0	0	0	999	999
26/10/2024 21:30	1	8	0	0	0	0	0	999	999
26/10/2024 21:45	1	8.3	0	0	0	0	0	999	999
26/10/2024 22:00	1	8.3	0	0	0	0	0	999	999
26/10/2024 22:15	1	8.4	0	0	0	0	0	999	999
26/10/2024 22:30	1	8.6	0	0	0	0	1	999	999
26/10/2024 22:45	0.003906	8.4	0	0	0	0	0	999	999
26/10/2024 23:00	0.003906	8.5	0	0	0	0	0	999	999
26/10/2024 23:15	0.003906	8.6	0	0	0	0	0	999	999
26/10/2024 23:30	0.003906	8.6	0	0	0	0	0	999	999
26/10/2024 23:45	0.035156	8.6	0	0	0	0	0	999	999
27/10/2024 00:00	0.144531	8.3	0	0	0	0	0	999	999
27/10/2024 00:15	0.363281	8.4	0	0	0	0	0	999	999
27/10/2024 00:30	0.636719	8.6	0	0	0	0	0	999	999
27/10/2024 00:45	0.855469	8.6	0	0	0	0	0	999	999
27/10/2024 01:00	0.964844	9	0	0	0	0	0	999	999
27/10/2024 01:15	0.996094	8.4	0	0	0	0	0	999	999
27/10/2024 01:30	1	8.4	0	0	0	1	0	999	999

27/10/2024 01:45	1	8.7	0	0	0	0	0	999	999
27/10/2024 02:00	1	8.4	0	0	0	0	0	999	999
27/10/2024 02:15	1	8.5	0	0	0	0	0	999	999
27/10/2024 02:30	1	8.6	0	0	0	0	0	999	999
27/10/2024 02:45	1	8.7	0	0	0	0	0	999	999
27/10/2024 03:00	1	8.6	0	0	0	0	0	999	999
27/10/2024 03:15	1	8.6	0	0	0	0	0	999	999
27/10/2024 03:30	1	8.6	0	0	0	0	0	999	999
27/10/2024 03:45	1	8.5	0	0	0	0	1	999	999
27/10/2024 04:00	0.035156	8.6	0	0	0	0	0	999	999
27/10/2024 04:15	0.144531	8.8	0	0	0	0	0	999	999
27/10/2024 04:30	0.363281	8.8	0	0	0	0	0	999	999
27/10/2024 04:45	0.636719	8.7	0	0	0	0	0	999	999
27/10/2024 05:00	0.855469	8.7	0	0	0	0	0	999	999
27/10/2024 05:15	0.964844	8.1	0	0	0	0	0	999	999
27/10/2024 05:30	0.996094	8.5	0	0	0	0	0	999	999
27/10/2024 05:45	1	8.6	0	0	0	1	0	999	999
27/10/2024 06:00	1	8.5	0	0	0	0	0	999	999
27/10/2024 06:15	1	8.7	0	0	0	0	0	999	999
27/10/2024 06:30	1	8.5	0	0	0	0	0	999	999
27/10/2024 06:45	1	9.1	0	0	0	0	0	999	999
27/10/2024 07:00	1	8.7	0	0	0	0	0	999	999
27/10/2024 07:15	1	8.7	0	0	0	0	0	999	999
27/10/2024 07:30	1	8.6	0	0	0	0	0	999	999
27/10/2024 07:45	1	8.5	0	0	0	0	0	999	999
27/10/2024 08:00	1	8.7	0	0	0	0	1	999	999
27/10/2024 08:15	0.035156	9	0	0	0	0	0	999	999
27/10/2024 08:30	0.144531	8.7	0	0	0	0	0	999	999
27/10/2024 08:45	0.363281	9	0	0	0	0	0	999	999
27/10/2024 09:00	0.636719	8.8	0	0	0	0	0	999	999
27/10/2024 09:15	0.855469	8.7	0	0	0	0	0	999	999
27/10/2024 09:30	0.964844	8.9	0	0	0	0	0	999	999
27/10/2024 09:45	0.996094	8.9	0	0	0	0	0	999	999
27/10/2024 10:00	1	8.9	0	0	0	1	0	999	999
27/10/2024 10:15	1	9.1	0	0	0	0	0	999	999
27/10/2024 10:30	1	8.7	0	0	0	0	0	999	999

27/10/2024 10:45	1	8.7	0	0	0	0	0	999	999
27/10/2024 11:00	1	9	0	0	0	0	0	999	999
27/10/2024 11:15	1	8.8	0	0	0	0	0	999	999
27/10/2024 11:30	1	8.7	0	0	0	0	0	999	999
27/10/2024 11:45	1	8.8	0	0	0	0	0	999	999
27/10/2024 12:00	1	8.8	0	0	0	0	0	999	999
27/10/2024 12:15	1	8.1	0	0	0	0	1	999	999
27/10/2024 12:30	0.035156	8.9	0	0	0	0	0	999	999
27/10/2024 12:45	0.144531	8.6	0	0	0	0	0	999	999
27/10/2024 13:00	0.363281	8.6	0	0	0	0	0	999	999
27/10/2024 13:15	0.636719	8.6	0	0	0	0	0	999	999
27/10/2024 13:30	0.855469	8.7	0	0	0	0	0	999	999
27/10/2024 13:45	0.964844	8.6	0	0	0	0	0	999	999
27/10/2024 14:00	0.996094	8.6	0	0	0	0	0	999	999
27/10/2024 14:15	1	10	0	0	0	1	0	999	999
27/10/2024 14:30	1	8.7	0	0	0	0	0	999	999
27/10/2024 14:45	1	8.4	0	0	0	0	0	999	999
27/10/2024 15:00	1	8.9	0	0	0	0	0	999	999
27/10/2024 15:15	1	8.6	0	0	0	0	0	999	999
27/10/2024 15:30	1	8.7	0	0	0	0	0	999	999
27/10/2024 15:45	1	8.6	0	0	0	0	0	999	999
27/10/2024 16:00	1	8.6	0	0	0	0	0	999	999
27/10/2024 16:15	1	8.5	0	0	0	0	0	999	999
27/10/2024 16:30	1	8.5	0	0	0	0	1	999	999
27/10/2024 16:45	0.003906	8.6	0	0	0	0	0	999	999
27/10/2024 17:00	0.035156	8.5	0	0	0	0	0	999	999
27/10/2024 17:15	0.144531	8.1	0	0	0	0	0	999	999
27/10/2024 17:30	0.363281	8.7	0	0	0	0	0	999	999
27/10/2024 17:45	0.636719	8.5	0	0	0	0	0	999	999
27/10/2024 18:00	0.855469	8.5	0	0	0	0	0	999	999
27/10/2024 18:15	0.964844	8.5	0	0	0	0	0	999	999
27/10/2024 18:30	0.996094	8.4	0	0	0	0	0	999	999
27/10/2024 18:45	1	8.6	0	0	0	1	0	999	999
27/10/2024 19:00	1	8.2	0	0	0	0	0	999	999
27/10/2024 19:15	1	8.3	0	0	0	0	0	999	999
27/10/2024 19:30	1	8.1	0	0	0	0	0	999	999

27/10/2024 19:45	1	8.3	0	0	0	0	0	999	999
27/10/2024 20:00	1	8.6	0	0	0	0	0	999	999
27/10/2024 20:15	1	8.6	0	0	0	0	0	999	999
27/10/2024 20:30	1	8.6	0	0	0	0	0	999	999
27/10/2024 20:45	1	8.1	0	0	0	0	0	999	999
27/10/2024 21:00	1	8.5	0	0	0	0	1	999	999
27/10/2024 21:15	0.003906	8.3	0	0	0	0	0	999	999
27/10/2024 21:30	0.003906	8.3	0	0	0	0	0	999	999
27/10/2024 21:45	0.003906	8.7	0	0	0	0	0	999	999
27/10/2024 22:00	0.035156	8.8	0	0	0	0	0	999	999
27/10/2024 22:15	0.144531	8.7	0	0	0	0	0	999	999
27/10/2024 22:30	0.363281	8.5	0	0	0	0	0	999	999
27/10/2024 22:45	0.363281	8.3	0	0	0	0	0	999	999
27/10/2024 23:00	0.363281	8.3	0	0	0	0	0	999	999
27/10/2024 23:15	0.363281	8.4	0	0	0	0	0	999	999
27/10/2024 23:30	0.363281	8.1	0	0	0	0	0	999	999
27/10/2024 23:45	0.636719	8.2	0	0	0	0	0	999	999
28/10/2024 00:00	0.636719	8.4	0	0	0	0	0	999	999
28/10/2024 00:15	0.636719	7.8	0	0	0	0	0	999	999
28/10/2024 00:30	0.636719	8.1	0	0	0	0	0	999	999
28/10/2024 00:45	0.855469	8.2	0	0	0	0	0	999	999
28/10/2024 01:00	0.964844	8.1	0	0	0	0	0	999	999
28/10/2024 01:15	0.996094	7.8	0	0	0	0	0	999	999
28/10/2024 01:30	1	7.8	0	0	0	1	0	999	999
28/10/2024 01:45	1	7.8	0	0	0	0	0	999	999
28/10/2024 02:00	1	7.6	0	0	0	0	0	999	999
28/10/2024 02:15	1	7.7	0	0	0	0	0	999	999
28/10/2024 02:30	1	7.8	0	0	0	0	0	999	999
28/10/2024 02:45	1	7.8	0	0	0	0	0	999	999
28/10/2024 03:00	1	7.6	0	0	0	0	0	999	999
28/10/2024 03:15	1	7.5	0	0	0	0	0	999	999
28/10/2024 03:30	1	7.4	0	0	0	0	0	999	999
28/10/2024 03:45	1	7.6	0	0	0	0	1	999	999
28/10/2024 04:00	0.003906	7.6	0	0	0	0	0	999	999
28/10/2024 04:15	0.003906	7.9	0	0	0	0	0	999	999
28/10/2024 04:30	0.003906	7.6	0	0	0	0	0	999	999

28/10/2024 04:45	0.003906	7.5	0	0	0	0	0	999	999
28/10/2024 05:00	0.003906	7.8	0	0	0	0	0	999	999
28/10/2024 05:15	0.003906	7.8	0	0	0	0	0	999	999
28/10/2024 05:30	0.035156	7.5	0	0	0	0	0	999	999
28/10/2024 05:45	0.144531	7.5	0	0	0	0	0	999	999
28/10/2024 06:00	0.363281	7.8	0	0	0	0	0	999	999
28/10/2024 06:15	0.636719	7.8	0	0	0	0	0	999	999
28/10/2024 06:30	0.855469	7.8	0	0	0	0	0	999	999
28/10/2024 06:45	0.964844	7.8	0	0	0	0	0	999	999
28/10/2024 07:00	0.996094	7.7	0	0	0	0	0	999	999
28/10/2024 07:15	1	8.3	0	0	0	1	0	999	999
28/10/2024 07:30	1	7.5	0	0	0	0	0	999	999
28/10/2024 07:45	1	7.6	0	0	0	0	0	999	999
28/10/2024 08:00	1	7.4	0	0	0	0	0	999	999
28/10/2024 08:15	1	7.8	0	0	0	0	0	999	999
28/10/2024 08:30	1	7.6	0	0	0	0	0	999	999
28/10/2024 08:45	1	7.8	0	0	0	0	0	999	999
28/10/2024 09:00	1	7.9	0	0	0	0	0	999	999
28/10/2024 09:15	1	7.8	0	0	0	0	0	999	999
28/10/2024 09:30	1	7.9	0	0	0	0	1	999	999
28/10/2024 09:45	0.003906	7.9	0	0	0	0	0	999	999
28/10/2024 10:00	0.035156	7.6	0	0	0	0	0	999	999
28/10/2024 10:15	0.144531	7.4	0	0	0	0	0	999	999
28/10/2024 10:30	0.363281	7.2	0	0	0	0	0	999	999
28/10/2024 10:45	0.636719	7.4	0	0	0	0	0	999	999
28/10/2024 11:00	0.855469	7.6	0	0	0	0	0	999	999
28/10/2024 11:15	0.964844	7.2	0	0	0	0	0	999	999
28/10/2024 11:30	0.996094	7.4	0	0	0	0	0	999	999
28/10/2024 11:45	1	7.3	0	0	0	1	0	999	999
28/10/2024 12:00	1	7.5	0	0	0	0	0	999	999
28/10/2024 12:15	1	7.2	0	0	0	0	0	999	999
28/10/2024 12:30	1	7.3	0	0	0	0	0	999	999
28/10/2024 12:45	1	7.2	0	0	0	0	0	999	999
28/10/2024 13:00	1	7.1	0	0	0	0	0	999	999
28/10/2024 13:15	1	7.2	0	0	0	0	0	999	999
28/10/2024 13:30	1	6.9	0	0	0	0	0	999	999

28/10/2024 13:45	1	7.3	0	0	0	0	0	999	999
28/10/2024 14:00	1	6.8	0	0	0	0	1	999	999
28/10/2024 14:15	0.035156	6.8	0	0	0	0	0	999	999
28/10/2024 14:30	0.144531	6.9	0	0	0	0	0	999	999
28/10/2024 14:45	0.144531	6.7	0	0	0	0	0	999	999
28/10/2024 15:00	0.363281	7	0	0	0	0	0	999	999
28/10/2024 15:15	0.363281	6.8	0	0	0	0	0	999	999
28/10/2024 15:30	0.363281	6.7	0	0	0	0	0	999	999
28/10/2024 15:45	0.636719	6.5	0	0	0	0	0	999	999
28/10/2024 16:00	0.855469	6.6	0	0	0	0	0	999	999
28/10/2024 16:15	0.855469	6.8	0	0	0	0	0	999	999
28/10/2024 16:30	0.855469	6.3	0	0	0	0	0	999	999
28/10/2024 16:45	0.964844	6.4	0	0	0	0	0	999	999
28/10/2024 17:00	0.964844	6.3	0	0	0	0	0	999	999
28/10/2024 17:15	0.996094	6.5	0	0	0	0	0	999	999
28/10/2024 17:30	1	6.6	0	0	0	1	0	999	999
28/10/2024 17:45	1	6.4	0	0	0	0	0	999	999
28/10/2024 18:00	1	6.4	0	0	0	0	0	999	999
28/10/2024 18:15	1	6.5	0	0	0	0	0	999	999
28/10/2024 18:30	1	6.1	0	0	0	0	0	999	999
28/10/2024 18:45	1	6.2	0	0	0	0	0	999	999
28/10/2024 19:00	1	6.2	0	0	0	0	0	999	999
28/10/2024 19:15	1	6.5	0	0	0	0	0	999	999
28/10/2024 19:30	1	6.2	0	0	0	0	1	999	999
28/10/2024 19:45	0.003906	6.4	0	0	0	0	0	999	999
28/10/2024 20:00	0.003906	6.9	0	0	0	0	0	999	999
28/10/2024 20:15	0.035156	6.4	0	0	0	0	0	999	999
28/10/2024 20:30	0.144531	6.1	0	0	0	0	0	999	999
28/10/2024 20:45	0.363281	6.2	0	0	0	0	0	999	999
28/10/2024 21:00	0.636719	6.4	0	0	0	0	0	999	999
28/10/2024 21:15	0.855469	6.2	0	0	0	0	0	999	999
28/10/2024 21:30	0.964844	6.4	0	0	0	0	0	999	999
28/10/2024 21:45	0.996094	6.3	0	0	0	0	0	999	999
28/10/2024 22:00	1	6.4	0	0	0	1	0	999	999
28/10/2024 22:15	1	6.5	0	0	0	0	0	999	999
28/10/2024 22:30	1	6.2	0	0	0	0	0	999	999

28/10/2024 22:45	1	6.4	0	0	0	0	0	999	999
28/10/2024 23:00	1	6.2	0	0	0	0	0	999	999
28/10/2024 23:15	1	6.1	0	0	0	0	0	999	999
28/10/2024 23:30	1	6.2	0	0	0	0	0	999	999
28/10/2024 23:45	1	6.2	0	0	0	0	0	999	999
29/10/2024 00:00	1	6.2	0	0	0	0	0	999	999
29/10/2024 00:15	1	6.1	0	0	0	0	1	999	999
29/10/2024 00:30	0.035156	5.7	0	0	0	0	0	999	999
29/10/2024 00:45	0.144531	6.2	0	0	0	0	0	999	999
29/10/2024 01:00	0.144531	6.2	0	0	0	0	0	999	999
29/10/2024 01:15	0.363281	6	0	0	0	0	0	999	999
29/10/2024 01:30	0.636719	5.9	0	0	0	0	0	999	999
29/10/2024 01:45	0.855469	5.9	0	0	0	0	0	999	999
29/10/2024 02:00	0.964844	5.7	0	0	0	0	0	999	999
29/10/2024 02:15	0.996094	6.1	0	0	0	0	0	999	999
29/10/2024 02:30	0.996094	6.1	0	0	0	0	0	999	999
29/10/2024 02:45	0.996094	6.1	0	0	0	0	0	999	999
29/10/2024 03:00	1	6.1	0	0	0	1	0	999	999
29/10/2024 03:15	1	6.1	0	0	0	0	0	999	999
29/10/2024 03:30	1	5.7	0	0	0	0	0	999	999
29/10/2024 03:45	1	5.9	0	0	0	0	0	999	999
29/10/2024 04:00	1	5.8	0	0	0	0	0	999	999
29/10/2024 04:15	1	6	0	0	0	0	0	999	999
29/10/2024 04:30	1	5.7	0	0	0	0	0	999	999
29/10/2024 04:45	1	5.7	0	0	0	0	1	999	999
29/10/2024 05:00	0.035156	5.7	0	0	0	0	0	999	999
29/10/2024 05:15	0.144531	5.6	0	0	0	0	0	999	999
29/10/2024 05:30	0.363281	5.7	0	0	0	0	0	999	999
29/10/2024 05:45	0.636719	6.1	0	0	0	0	0	999	999
29/10/2024 06:00	0.855469	5.7	0	0	0	0	0	999	999
29/10/2024 06:15	0.964844	5.5	0	0	0	0	0	999	999
29/10/2024 06:30	0.996094	5.9	0	0	0	0	0	999	999
29/10/2024 06:45	1	5.6	0	0	0	1	0	999	999
29/10/2024 07:00	1	5.6	0	0	0	0	0	999	999
29/10/2024 07:15	1	5.7	0	0	0	0	0	999	999
29/10/2024 07:30	1	5.7	0	0	0	0	0	999	999

29/10/2024 07:45	1	5.6	0	0	0	0	0	999	999
29/10/2024 08:00	1	5.7	0	0	0	0	0	999	999
29/10/2024 08:15	1	5.7	0	0	0	0	0	999	999
29/10/2024 08:30	1	5.5	0	0	0	0	0	999	999
29/10/2024 08:45	1	5.7	0	0	0	0	0	999	999
29/10/2024 09:00	1	5.9	0	0	0	0	1	999	999
29/10/2024 09:15	0.003906	5.7	0	0	0	0	0	999	999
29/10/2024 09:30	0.003906	5.7	0	0	0	0	0	999	999
29/10/2024 09:45	0.003906	5.7	0	0	0	0	0	999	999
29/10/2024 10:00	0.003906	5.6	0	0	0	0	0	999	999
29/10/2024 10:15	0.003906	5.5	0	0	0	0	0	999	999
29/10/2024 10:30	0.003906	5.7	0	0	0	0	0	999	999
29/10/2024 10:45	0.003906	5.5	0	0	0	0	0	999	999
29/10/2024 11:00	0.035156	5.6	0	0	0	0	0	999	999
29/10/2024 11:15	0.144531	5.4	0	0	0	0	0	999	999
29/10/2024 11:30	0.363281	5.6	0	0	0	0	0	999	999
29/10/2024 11:45	0.636719	5.6	0	0	0	0	0	999	999
29/10/2024 12:00	0.855469	5.4	0	0	0	0	0	999	999
29/10/2024 12:15	0.964844	5.4	0	0	0	0	0	999	999
29/10/2024 12:30	0.996094	5.4	0	0	0	0	0	999	999
29/10/2024 12:45	1	5.4	0	0	0	1	0	999	999
29/10/2024 13:00	1	5.3	0	0	0	0	0	999	999
29/10/2024 13:15	1	5.4	0	0	0	0	0	999	999
29/10/2024 13:30	1	5.1	0	0	0	0	0	999	999
29/10/2024 13:45	1	5.6	0	0	0	0	0	999	999
29/10/2024 14:00	1	5.4	0	0	0	0	0	999	999
29/10/2024 14:15	1	5.1	0	0	0	0	0	999	999
29/10/2024 14:30	1	5.1	0	0	0	0	0	999	999
29/10/2024 14:45	1	5	0	0	0	0	0	999	999
29/10/2024 15:00	1	4.8	0	0	0	0	1	999	999
29/10/2024 15:15	0.003906	5.5	0	0	0	0	0	999	999
29/10/2024 15:30	0.035156	5.3	0	0	0	0	0	999	999
29/10/2024 15:45	0.144531	5.1	0	0	0	0	0	999	999
29/10/2024 16:00	0.144531	4.7	0	0	0	0	0	999	999
29/10/2024 16:15	0.144531	5	0	0	0	0	0	999	999
29/10/2024 16:30	0.144531	4.8	0	0	0	0	0	999	999

29/10/2024 16:45	0.144531	4.8	0	0	0	0	0	999	999
29/10/2024 17:00	0.363281	4.8	0	0	0	0	0	999	999
29/10/2024 17:15	0.636719	5	0	0	0	0	0	999	999
29/10/2024 17:30	0.636719	4.9	0	0	0	0	0	999	999
29/10/2024 17:45	0.636719	0.3	0	0	0	0	0	999	999
29/10/2024 18:00	0.855469	4.9	0	0	0	0	0	999	999
29/10/2024 18:15	0.964844	4.7	0	0	0	0	0	999	999
29/10/2024 18:30	0.996094	4.7	0	0	0	0	0	999	999
29/10/2024 18:45	1	4.5	0	0	0	1	0	999	999
29/10/2024 19:00	1	4.7	0	0	0	0	0	999	999
29/10/2024 19:15	1	5	0	0	0	0	0	999	999
29/10/2024 19:30	1	5.2	0	0	0	0	0	999	999
29/10/2024 19:45	1	4.8	0	0	0	0	0	999	999
29/10/2024 20:00	1	5.1	0	0	0	0	0	999	999
29/10/2024 20:15	1	4.7	0	0	0	0	0	999	999
29/10/2024 20:30	1	4.8	0	0	0	0	0	999	999
29/10/2024 20:45	1	5	0	0	0	0	0	999	999
29/10/2024 21:00	1	4.8	0	0	0	0	1	999	999
29/10/2024 21:15	0.003906	5	0	0	0	0	0	999	999
29/10/2024 21:30	0.003906	4.9	0	0	0	0	0	999	999
29/10/2024 21:45	0.003906	4.7	0	0	0	0	0	999	999
29/10/2024 22:00	0.003906	4.8	0	0	0	0	0	999	999
29/10/2024 22:15	0.003906	4.7	0	0	0	0	0	999	999
29/10/2024 22:30	0.003906	4.8	0	0	0	0	0	999	999
29/10/2024 22:45	0.003906	5.1	0	0	0	0	0	999	999
29/10/2024 23:00	0.003906	5.5	0	0	0	0	0	999	999
29/10/2024 23:15	0.003906	4.6	0	0	0	0	0	999	999
29/10/2024 23:30	0.003906	5	0	0	0	0	0	999	999
29/10/2024 23:45	0.003906	5.1	0	0	0	0	0	999	999
30/10/2024 00:00	0.003906	5	0	0	0	0	0	999	999
30/10/2024 00:15	0.035156	5.1	0	0	0	0	0	999	999
30/10/2024 00:30	0.144531	5	0	0	0	0	0	999	999
30/10/2024 00:45	0.363281	4.9	0	0	0	0	0	999	999
30/10/2024 01:00	0.636719	4.9	0	0	0	0	0	999	999
30/10/2024 01:15	0.855469	4.8	0	0	0	0	0	999	999
30/10/2024 01:30	0.964844	4.8	0	0	0	0	0	999	999

30/10/2024 01:45	0.996094	4.3	0	0	0	0	0	999	999
30/10/2024 02:00	1	4.8	0	0	0	1	0	999	999
30/10/2024 02:15	1	5.2	0	0	0	0	0	999	999
30/10/2024 02:30	1	4.8	0	0	0	0	0	999	999
30/10/2024 02:45	1	4.8	0	0	0	0	0	999	999
30/10/2024 03:00	1	4.6	0	0	0	0	0	999	999
30/10/2024 03:15	1	4.8	0	0	0	0	0	999	999
30/10/2024 03:30	1	4.7	0	0	0	0	0	999	999
30/10/2024 03:45	1	4.8	0	0	0	0	0	999	999
30/10/2024 04:00	1	4.9	0	0	0	0	0	999	999
30/10/2024 04:15	1	4.6	0	0	0	0	1	999	999
30/10/2024 04:30	0.035156	4.6	0	0	0	0	0	999	999
30/10/2024 04:45	0.144531	4.8	0	0	0	0	0	999	999
30/10/2024 05:00	0.144531	4.5	0	0	0	0	0	999	999
30/10/2024 05:15	0.363281	4.6	0	0	0	0	0	999	999
30/10/2024 05:30	0.636719	4.6	0	0	0	0	0	999	999
30/10/2024 05:45	0.855469	4.9	0	0	0	0	0	999	999
30/10/2024 06:00	0.855469	4.6	0	0	0	0	0	999	999
30/10/2024 06:15	0.964844	4.6	0	0	0	0	0	999	999
30/10/2024 06:30	0.964844	4.5	0	0	0	0	0	999	999
30/10/2024 06:45	0.964844	4.5	0	0	0	0	0	999	999
30/10/2024 07:00	0.996094	4.8	0	0	0	0	0	999	999
30/10/2024 07:15	0.996094	4.9	0	0	0	0	0	999	999
30/10/2024 07:30	0.964844	4.8	0	0	0	0	0	999	999
30/10/2024 07:45	0.855469	4.7	0	0	0	0	0	999	999
30/10/2024 08:00	0.855469	4.7	0	0	0	0	0	999	999
30/10/2024 08:15	0.636719	4.8	0	0	0	0	0	999	999
30/10/2024 08:30	0.363281	4.8	0	0	0	0	0	999	999
30/10/2024 08:45	0.144531	4.9	0	0	0	0	0	999	999
30/10/2024 09:00	0.035156	5	0	0	0	0	0	999	999
30/10/2024 09:15	0.003906	4.5	0	0	0	0	0	999	999
30/10/2024 09:30	0.035156	4.8	0	0	0	0	0	999	999
30/10/2024 09:45	0.035156	4.8	0	0	0	0	0	999	999
30/10/2024 10:00	0.035156	4.8	0	0	0	0	0	999	999
30/10/2024 10:15	0.035156	4.5	0	0	0	0	0	999	999
30/10/2024 10:30	0.035156	4.4	0	0	0	0	0	999	999

30/10/2024 10:45	0.035156	4.9	0	0	0	0	0	999	999
30/10/2024 11:00	0.144531	4.7	0	0	0	0	0	999	999
30/10/2024 11:15	0.363281	4.8	0	0	0	0	0	999	999
30/10/2024 11:30	0.363281	4.6	0	0	0	0	0	999	999
30/10/2024 11:45	0.636719	4.6	0	0	0	0	0	999	999
30/10/2024 12:00	0.855469	4.8	0	0	0	0	0	999	999
30/10/2024 12:15	0.964844	4.6	0	0	0	0	0	999	999
30/10/2024 12:30	0.996094	4.5	0	0	0	0	0	999	999
30/10/2024 12:45	1	4.5	0	0	0	1	0	999	999
30/10/2024 13:00	1	4.4	0	0	0	0	0	999	999
30/10/2024 13:15	1	4.2	0	0	0	0	0	999	999
30/10/2024 13:30	1	4.3	0	0	0	0	0	999	999
30/10/2024 13:45	1	4.5	0	0	0	0	0	999	999
30/10/2024 14:00	1	4.5	0	0	0	0	0	999	999
30/10/2024 14:15	1	4.3	0	0	0	0	0	999	999
30/10/2024 14:30	1	4.5	0	0	0	0	0	999	999
30/10/2024 14:45	1	4.7	0	0	0	0	0	999	999
30/10/2024 15:00	1	4.5	0	0	0	0	1	999	999
30/10/2024 15:15	0.003906	4.5	0	0	0	0	0	999	999
30/10/2024 15:30	0.003906	4.3	0	0	0	0	0	999	999
30/10/2024 15:45	0.003906	4.3	0	0	0	0	0	999	999
30/10/2024 16:00	0.003906	4.5	0	0	0	0	0	999	999
30/10/2024 16:15	0.003906	4.5	0	0	0	0	0	999	999
30/10/2024 16:30	0.003906	4.3	0	0	0	0	0	999	999
30/10/2024 16:45	0.003906	4.3	0	0	0	0	0	999	999
30/10/2024 17:00	0.003906	4	0	0	0	0	0	999	999
30/10/2024 17:15	0.035156	4.5	0	0	0	0	0	999	999
30/10/2024 17:30	0.035156	4.3	0	0	0	0	0	999	999
30/10/2024 17:45	0.144531	4.4	0	0	0	0	0	999	999
30/10/2024 18:00	0.363281	4.4	0	0	0	0	0	999	999
30/10/2024 18:15	0.636719	4.3	0	0	0	0	0	999	999
30/10/2024 18:30	0.855469	4.7	0	0	0	0	0	999	999
30/10/2024 18:45	0.964844	4.3	0	0	0	0	0	999	999
30/10/2024 19:00	0.996094	4.4	0	0	0	0	0	999	999
30/10/2024 19:15	0.996094	4.4	0	0	0	0	0	999	999
30/10/2024 19:30	1	4.4	0	0	0	1	0	999	999

30/10/2024 19:45	1	4.5	0	0	0	0	0	999	999
30/10/2024 20:00	1	4.5	0	0	0	0	0	999	999
30/10/2024 20:15	1	4.5	0	0	0	0	0	999	999
30/10/2024 20:30	1	4.7	0	0	0	0	0	999	999
30/10/2024 20:45	1	4.5	0	0	0	0	0	999	999
30/10/2024 21:00	1	4.3	0	0	0	0	0	999	999
30/10/2024 21:15	1	4.8	0	0	0	0	0	999	999
30/10/2024 21:30	1	4.2	0	0	0	0	1	999	999
30/10/2024 21:45	0.003906	4.5	0	0	0	0	0	999	999
30/10/2024 22:00	0.003906	4.3	0	0	0	0	0	999	999
30/10/2024 22:15	0.003906	4.4	0	0	0	0	0	999	999
30/10/2024 22:30	0.003906	4.3	0	0	0	0	0	999	999
30/10/2024 22:45	0.035156	4.5	0	0	0	0	0	999	999
30/10/2024 23:00	0.144531	4.4	0	0	0	0	0	999	999
30/10/2024 23:15	0.363281	4.3	0	0	0	0	0	999	999
30/10/2024 23:30	0.636719	4.5	0	0	0	0	0	999	999
30/10/2024 23:45	0.855469	4.3	0	0	0	0	0	999	999
31/10/2024 00:00	0.964844	4.2	0	0	0	0	0	999	999
31/10/2024 00:15	0.996094	4.2	0	0	0	0	0	999	999
31/10/2024 00:30	1	4.2	0	0	0	1	0	999	999
31/10/2024 00:45	1	4.5	0	0	0	0	0	999	999
31/10/2024 01:00	1	4.4	0	0	0	0	0	999	999
31/10/2024 01:15	1	4.3	0	0	0	0	0	999	999
31/10/2024 01:30	1	4.3	0	0	0	0	0	999	999
31/10/2024 01:45	1	4.5	0	0	0	0	0	999	999
31/10/2024 02:00	1	4.2	0	0	0	0	0	999	999
31/10/2024 02:15	1	4.2	0	0	0	0	0	999	999
31/10/2024 02:30	1	4.1	0	0	0	0	0	999	999
31/10/2024 02:45	1	4.2	0	0	0	0	1	999	999
31/10/2024 03:00	0.035156	4.3	0	0	0	0	0	999	999
31/10/2024 03:15	0.144531	4.5	0	0	0	0	0	999	999
31/10/2024 03:30	0.363281	4	0	0	0	0	0	999	999
31/10/2024 03:45	0.636719	4.5	0	0	0	0	0	999	999
31/10/2024 04:00	0.855469	4.3	0	0	0	0	0	999	999
31/10/2024 04:15	0.964844	4.1	0	0	0	0	0	999	999
31/10/2024 04:30	0.996094	4.2	0	0	0	0	0	999	999

31/10/2024 04:45	1	4.3	0	0	0	1	0	999	999
31/10/2024 05:00	1	3.9	0	0	0	0	0	999	999
31/10/2024 05:15	1	3.9	0	0	0	0	0	999	999
31/10/2024 05:30	1	4	0	0	0	0	0	999	999
31/10/2024 05:45	1	4.2	0	0	0	0	0	999	999
31/10/2024 06:00	1	4	0	0	0	0	0	999	999
31/10/2024 06:15	1	4	0	0	0	0	0	999	999
31/10/2024 06:30	1	4	0	0	0	0	0	999	999
31/10/2024 06:45	1	4.1	0	0	0	0	0	999	999
31/10/2024 07:00	1	4	0	0	0	0	1	999	999
31/10/2024 07:15	0.035156	4.3	0	0	0	0	0	999	999
31/10/2024 07:30	0.144531	4.2	0	0	0	0	0	999	999
31/10/2024 07:45	0.363281	3.9	0	0	0	0	0	999	999
31/10/2024 08:00	0.636719	4	0	0	0	0	0	999	999
31/10/2024 08:15	0.855469	3.9	0	0	0	0	0	999	999
31/10/2024 08:30	0.964844	4.2	0	0	0	0	0	999	999
31/10/2024 08:45	0.996094	3.9	0	0	0	0	0	999	999
31/10/2024 09:00	1	4.1	0	0	0	1	0	999	999
31/10/2024 09:15	1	4	0	0	0	0	0	999	999
31/10/2024 09:30	1	4	0	0	0	0	0	999	999
31/10/2024 09:45	1	3.9	0	0	0	0	0	999	999
31/10/2024 10:00	1	3.9	0	0	0	0	0	999	999
31/10/2024 10:15	1	4	0	0	0	0	0	999	999
31/10/2024 10:30	1	4.1	0	0	0	0	0	999	999
31/10/2024 10:45	1	4.5	0	0	0	0	0	999	999
31/10/2024 11:00	1	4.2	0	0	0	0	0	999	999
31/10/2024 11:15	1	4	0	0	0	0	1	999	999
31/10/2024 11:30	0.003906	4.1	0	0	0	0	0	999	999
31/10/2024 11:45	0.035156	4	0	0	0	0	0	999	999
31/10/2024 12:00	0.144531	4	0	0	0	0	0	999	999
31/10/2024 12:15	0.363281	4	0	0	0	0	0	999	999
31/10/2024 12:30	0.636719	3.9	0	0	0	0	0	999	999
31/10/2024 12:45	0.855469	3.9	0	0	0	0	0	999	999
31/10/2024 13:00	0.964844	3.9	0	0	0	0	0	999	999
31/10/2024 13:15	0.996094	4	0	0	0	0	0	999	999
31/10/2024 13:30	1	3.9	0	0	0	1	0	999	999

31/10/2024 13:45	1	3.9	0	0	0	0	0	999	999
31/10/2024 14:00	1	3.8	0	0	0	0	0	999	999
31/10/2024 14:15	1	3.8	0	0	0	0	0	999	999
31/10/2024 14:30	1	3.9	0	0	0	0	0	999	999
31/10/2024 14:45	1	3.6	0	0	0	0	0	999	999
31/10/2024 15:00	1	3.6	0	0	0	0	0	999	999
31/10/2024 15:15	1	3.7	0	0	0	0	0	999	999
31/10/2024 15:30	1	3.7	0	0	0	0	0	999	999
31/10/2024 15:45	1	3.7	0	0	0	0	1	999	999
31/10/2024 16:00	0.003906	3.6	0	0	0	0	0	999	999
31/10/2024 16:15	0.003906	3.5	0	0	0	0	0	999	999
31/10/2024 16:30	0.035156	3.6	0	0	0	0	0	999	999
31/10/2024 16:45	0.035156	3.8	0	0	0	0	0	999	999
31/10/2024 17:00	0.035156	4.2	0	0	0	0	0	999	999
31/10/2024 17:15	0.144531	3.6	0	0	0	0	0	999	999
31/10/2024 17:30	0.363281	3.8	0	0	0	0	0	999	999
31/10/2024 17:45	0.363281	3.8	0	0	0	0	0	999	999
31/10/2024 18:00	0.363281	3.6	0	0	0	0	0	999	999
31/10/2024 18:15	0.363281	3.7	0	0	0	0	0	999	999
31/10/2024 18:30	0.144531	3.6	0	0	0	0	0	999	999
31/10/2024 18:45	0.363281	3.9	0	0	0	0	0	999	999
31/10/2024 19:00	0.363281	3.9	0	0	0	0	0	999	999
31/10/2024 19:15	0.144531	3.7	0	0	0	0	0	999	999
31/10/2024 19:30	0.035156	3.6	0	0	0	0	0	999	999
31/10/2024 19:45	0.035156	3.6	0	0	0	0	0	999	999
31/10/2024 20:00	0.035156	3.9	0	0	0	0	0	999	999
31/10/2024 20:15	0.035156	3.8	0	0	0	0	0	999	999
31/10/2024 20:30	0.035156	3.9	0	0	0	0	0	999	999
31/10/2024 20:45	0.035156	3.9	0	0	0	0	0	999	999
31/10/2024 21:00	0.144531	4	0	0	0	0	0	999	999
31/10/2024 21:15	0.363281	3.8	0	0	0	0	0	999	999
31/10/2024 21:30	0.636719	3.9	0	0	0	0	0	999	999
31/10/2024 21:45	0.855469	3.9	0	0	0	0	0	999	999
31/10/2024 22:00	0.964844	4.2	0	0	0	0	0	999	999
31/10/2024 22:15	0.996094	3.7	0	0	0	0	0	999	999
31/10/2024 22:30	1	3.9	0	0	0	1	0	999	999

31/10/2024 22:45	1	3.9	0	0	0	0	0	999	999
31/10/2024 23:00	1	3.7	0	0	0	0	0	999	999
31/10/2024 23:15	1	3.7	0	0	0	0	0	999	999
31/10/2024 23:30	1	3.7	0	0	0	0	0	999	999
31/10/2024 23:45	1	3.6	0	0	0	0	0	999	999
01/11/2024 00:00	1	3.4	0	0	0	0	0	999	999
01/11/2024 00:15	1	3.7	0	0	0	0	0	999	999
01/11/2024 00:30	1	3.6	0	0	0	0	0	999	999
01/11/2024 00:45	1	3.8	0	0	0	0	1	999	999
01/11/2024 01:00	0.003906	3.6	0	0	0	0	0	999	999
01/11/2024 01:15	0.003906	3.8	0	0	0	0	0	999	999
01/11/2024 01:30	0.003906	3.6	0	0	0	0	0	999	999
01/11/2024 01:45	0.003906	3.4	0	0	0	0	0	999	999
01/11/2024 02:00	0.035156	3.4	0	0	0	0	0	999	999
01/11/2024 02:15	0.144531	3.6	0	0	0	0	0	999	999
01/11/2024 02:30	0.363281	3.7	0	0	0	0	0	999	999
01/11/2024 02:45	0.636719	3.3	0	0	0	0	0	999	999
01/11/2024 03:00	0.855469	3.4	0	0	0	0	0	999	999
01/11/2024 03:15	0.964844	3.6	0	0	0	0	0	999	999
01/11/2024 03:30	0.996094	3.4	0	0	0	0	0	999	999
01/11/2024 03:45	1	3.4	0	0	0	1	0	999	999
01/11/2024 04:00	1	3.4	0	0	0	0	0	999	999
01/11/2024 04:15	1	3.4	0	0	0	0	0	999	999
01/11/2024 04:30	1	3.6	0	0	0	0	0	999	999
01/11/2024 04:45	1	3.5	0	0	0	0	0	999	999
01/11/2024 05:00	1	3.3	0	0	0	0	0	999	999
01/11/2024 05:15	1	4.2	0	0	0	0	0	999	999
01/11/2024 05:30	1	3.6	0	0	0	0	0	999	999
01/11/2024 05:45	1	3.2	0	0	0	0	0	999	999
01/11/2024 06:00	1	3.3	0	0	0	0	1	999	999
01/11/2024 06:15	0.003906	3.2	0	0	0	0	0	999	999
01/11/2024 06:30	0.035156	3.4	0	0	0	0	0	999	999
01/11/2024 06:45	0.144531	3.6	0	0	0	0	0	999	999
01/11/2024 07:00	0.363281	3.3	0	0	0	0	0	999	999
01/11/2024 07:15	0.636719	3.6	0	0	0	0	0	999	999
01/11/2024 07:30	0.855469	3.4	0	0	0	0	0	999	999

01/11/2024 07:45	0.964844	3.3	0	0	0	0	0	999	999
01/11/2024 08:00	0.996094	3.4	0	0	0	0	0	999	999
01/11/2024 08:15	1	3.4	0	0	0	1	0	999	999
01/11/2024 08:30	1	3.5	0	0	0	0	0	999	999
01/11/2024 08:45	1	3.4	0	0	0	0	0	999	999
01/11/2024 09:00	1	3.3	0	0	0	0	0	999	999
01/11/2024 09:15	1	3.2	0	0	0	0	0	999	999
01/11/2024 09:30	1	3.6	0	0	0	0	0	999	999
01/11/2024 09:45	1	3.6	0	0	0	0	0	999	999
01/11/2024 10:00	1	3.5	0	0	0	0	0	999	999
01/11/2024 10:15	1	3.4	0	0	0	0	0	999	999
01/11/2024 10:30	1	3.2	0	0	0	0	1	999	999
01/11/2024 10:45	0.035156	3.7	0	0	0	0	0	999	999
01/11/2024 11:00	0.035156	3.5	0	0	0	0	0	999	999
01/11/2024 11:15	0.035156	3.3	0	0	0	0	0	999	999
01/11/2024 11:30	0.035156	3.5	0	0	0	0	0	999	999
01/11/2024 11:45	0.035156	3.4	0	0	0	0	0	999	999
01/11/2024 12:00	0.144531	3.5	0	0	0	0	0	999	999
01/11/2024 12:15	0.363281	3.6	0	0	0	0	0	999	999
01/11/2024 12:30	0.636719	3.4	0	0	0	0	0	999	999
01/11/2024 12:45	0.636719	3.3	0	0	0	0	0	999	999
01/11/2024 13:00	0.855469	3.4	0	0	0	0	0	999	999
01/11/2024 13:15	0.964844	3.3	0	0	0	0	0	999	999
01/11/2024 13:30	0.996094	3.2	0	0	0	0	0	999	999
01/11/2024 13:45	1	3.2	0	0	0	1	0	999	999
01/11/2024 14:00	1	3.1	0	0	0	0	0	999	999
01/11/2024 14:15	1	3.4	0	0	0	0	0	999	999
01/11/2024 14:30	1	3.5	0	0	0	0	0	999	999
01/11/2024 14:45	1	3.5	0	0	0	0	0	999	999
01/11/2024 15:00	1	3.3	0	0	0	0	0	999	999
01/11/2024 15:15	1	3.5	0	0	0	0	0	999	999
01/11/2024 15:30	1	3.3	0	0	0	0	0	999	999
01/11/2024 15:45	1	3.5	0	0	0	0	0	999	999
01/11/2024 16:00	1	3.2	0	0	0	0	1	999	999
01/11/2024 16:15	0.003906	3.4	0	0	0	0	0	999	999
01/11/2024 16:30	0.003906	3.7	0	0	0	0	0	999	999

01/11/2024 16:45	0.035156	3	0	0	0	0	0	999	999
01/11/2024 17:00	0.144531	3.2	0	0	0	0	0	999	999
01/11/2024 17:15	0.144531	3.4	0	0	0	0	0	999	999
01/11/2024 17:30	0.144531	2.9	0	0	0	0	0	999	999
01/11/2024 17:45	0.363281	3.2	0	0	0	0	0	999	999
01/11/2024 18:00	0.363281	3.2	0	0	0	0	0	999	999
01/11/2024 18:15	0.363281	3.4	0	0	0	0	0	999	999
01/11/2024 18:30	0.636719	3.4	0	0	0	0	0	999	999
01/11/2024 18:45	0.636719	3.3	0	0	0	0	0	999	999
01/11/2024 19:00	0.636719	3.3	0	0	0	0	0	999	999
01/11/2024 19:15	0.855469	3.4	0	0	0	0	0	999	999
01/11/2024 19:30	0.964844	3.3	0	0	0	0	0	999	999
01/11/2024 19:45	0.964844	3.7	0	0	0	0	0	999	999
01/11/2024 20:00	0.996094	3.4	0	0	0	0	0	999	999
01/11/2024 20:15	1	3.4	0	0	0	1	0	999	999
01/11/2024 20:30	1	3.6	0	0	0	0	0	999	999
01/11/2024 20:45	1	3.4	0	0	0	0	0	999	999
01/11/2024 21:00	1	3.2	0	0	0	0	0	999	999
01/11/2024 21:15	1	3.4	0	0	0	0	0	999	999
01/11/2024 21:30	1	3.5	0	0	0	0	0	999	999
01/11/2024 21:45	1	3.6	0	0	0	0	0	999	999
01/11/2024 22:00	1	3.6	0	0	0	0	0	999	999
01/11/2024 22:15	1	3.6	0	0	0	0	1	999	999
01/11/2024 22:30	0.003906	3.4	0	0	0	0	0	999	999
01/11/2024 22:45	0.003906	3.6	0	0	0	0	0	999	999
01/11/2024 23:00	0.035156	3.5	0	0	0	0	0	999	999
01/11/2024 23:15	0.035156	3.6	0	0	0	0	0	999	999
01/11/2024 23:30	0.035156	3.4	0	0	0	0	0	999	999
01/11/2024 23:45	0.035156	3.6	0	0	0	0	0	999	999
02/11/2024 00:00	0.144531	3.7	0	0	0	0	0	999	999
02/11/2024 00:15	0.363281	3.4	0	0	0	0	0	999	999
02/11/2024 00:30	0.363281	3.4	0	0	0	0	0	999	999
02/11/2024 00:45	0.363281	3.3	0	0	0	0	0	999	999
02/11/2024 01:00	0.144531	3.6	0	0	0	0	0	999	999
02/11/2024 01:15	0.144531	3.6	0	0	0	0	0	999	999
02/11/2024 01:30	0.144531	3.6	0	0	0	0	0	999	999

02/11/2024 01:45	0.144531	3.4	0	0	0	0	0	999	999
02/11/2024 02:00	0.035156	3.6	0	0	0	0	0	999	999
02/11/2024 02:15	0.003906	3.4	0	0	0	0	0	999	999
02/11/2024 02:30	0.003906	3.4	0	0	0	0	0	999	999
02/11/2024 02:45	0.003906	3.4	0	0	0	0	0	999	999
02/11/2024 03:00	0.003906	3.3	0	0	0	0	0	999	999
02/11/2024 03:15	0.003906	3.4	0	0	0	0	0	999	999
02/11/2024 03:30	0.003906	3.6	0	0	0	0	0	999	999
02/11/2024 03:45	0.003906	3.4	0	0	0	0	0	999	999
02/11/2024 04:00	0.003906	3.5	0	0	0	0	0	999	999
02/11/2024 04:15	0.003906	3.4	0	0	0	0	0	999	999
02/11/2024 04:30	0.003906	3.5	0	0	0	0	0	999	999
02/11/2024 04:45	0.003906	3.3	0	0	0	0	0	999	999
02/11/2024 05:00	0.003906	3.4	0	0	0	0	0	999	999
02/11/2024 05:15	0.003906	3.4	0	0	0	0	0	999	999
02/11/2024 05:30	0.003906	3.3	0	0	0	0	0	999	999
02/11/2024 05:45	0.003906	3.3	0	0	0	0	0	999	999
02/11/2024 06:00	0.003906	3.2	0	0	0	0	0	999	999
02/11/2024 06:15	0.003906	3.3	0	0	0	0	0	999	999
02/11/2024 06:30	0.003906	3.3	0	0	0	0	0	999	999
02/11/2024 06:45	0.003906	3.4	0	0	0	0	0	999	999
02/11/2024 07:00	0.003906	3.5	0	0	0	0	0	999	999
02/11/2024 07:15	0.003906	3.4	0	0	0	0	0	999	999
02/11/2024 07:30	0.003906	3.2	0	0	0	0	0	999	999
02/11/2024 07:45	0.035156	3.2	0	0	0	0	0	999	999
02/11/2024 08:00	0.144531	3.4	0	0	0	0	0	999	999
02/11/2024 08:15	0.363281	3.2	0	0	0	0	0	999	999
02/11/2024 08:30	0.636719	3.5	0	0	0	0	0	999	999
02/11/2024 08:45	0.855469	3.4	0	0	0	0	0	999	999
02/11/2024 09:00	0.964844	3.2	0	0	0	0	0	999	999
02/11/2024 09:15	0.996094	3.2	0	0	0	0	0	999	999
02/11/2024 09:30	1	3.3	0	0	0	1	0	999	999
02/11/2024 09:45	1	3.2	0	0	0	0	0	999	999
02/11/2024 10:00	1	3.3	0	0	0	0	0	999	999
02/11/2024 10:15	1	3.3	0	0	0	0	0	999	999
02/11/2024 10:30	1	3.2	0	0	0	0	0	999	999

02/11/2024 10:45	1	3.5	0	0	0	0	0	999	999
02/11/2024 11:00	1	3.2	0	0	0	0	0	999	999
02/11/2024 11:15	1	3.2	0	0	0	0	0	999	999
02/11/2024 11:30	1	3.2	0	0	0	0	0	999	999
02/11/2024 11:45	1	3.2	0	0	0	0	1	999	999
02/11/2024 12:00	0.003906	3.3	0	0	0	0	0	999	999
02/11/2024 12:15	0.035156	3.1	0	0	0	0	0	999	999
02/11/2024 12:30	0.144531	3.2	0	0	0	0	0	999	999
02/11/2024 12:45	0.363281	3.3	0	0	0	0	0	999	999
02/11/2024 13:00	0.636719	3.3	0	0	0	0	0	999	999
02/11/2024 13:15	0.855469	3.4	0	0	0	0	0	999	999
02/11/2024 13:30	0.964844	3.1	0	0	0	0	0	999	999
02/11/2024 13:45	0.996094	3.3	0	0	0	0	0	999	999
02/11/2024 14:00	1	3.1	0	0	0	1	0	999	999
02/11/2024 14:15	1	3.1	0	0	0	0	0	999	999
02/11/2024 14:30	1	3.2	0	0	0	0	0	999	999
02/11/2024 14:45	1	3.1	0	0	0	0	0	999	999
02/11/2024 15:00	1	3.1	0	0	0	0	0	999	999
02/11/2024 15:15	1	3.2	0	0	0	0	0	999	999
02/11/2024 15:30	1	2.8	0	0	0	0	0	999	999
02/11/2024 15:45	1	3	0	0	0	0	0	999	999
02/11/2024 16:00	1	2.9	0	0	0	0	0	999	999
02/11/2024 16:15	1	3.1	0	0	0	0	1	999	999
02/11/2024 16:30	0.035156	3.2	0	0	0	0	0	999	999
02/11/2024 16:45	0.144531	3.1	0	0	0	0	0	999	999
02/11/2024 17:00	0.363281	3.1	0	0	0	0	0	999	999
02/11/2024 17:15	0.636719	3.1	0	0	0	0	0	999	999
02/11/2024 17:30	0.855469	2.9	0	0	0	0	0	999	999
02/11/2024 17:45	0.964844	3.2	0	0	0	0	0	999	999
02/11/2024 18:00	0.996094	3.4	0	0	0	0	0	999	999
02/11/2024 18:15	1	3.2	0	0	0	1	0	999	999
02/11/2024 18:30	1	3.1	0	0	0	0	0	999	999
02/11/2024 18:45	1	3.1	0	0	0	0	0	999	999
02/11/2024 19:00	1	3.1	0	0	0	0	0	999	999
02/11/2024 19:15	1	3.2	0	0	0	0	0	999	999
02/11/2024 19:30	1	3.2	0	0	0	0	0	999	999

02/11/2024 19:45	1	2.8	0	0	0	0	0	999	999
02/11/2024 20:00	1	3	0	0	0	0	0	999	999
02/11/2024 20:15	1	3.2	0	0	0	0	0	999	999
02/11/2024 20:30	1	3.2	0	0	0	0	1	999	999
02/11/2024 20:45	0.003906	3.3	0	0	0	0	0	999	999
02/11/2024 21:00	0.003906	3.5	0	0	0	0	0	999	999
02/11/2024 21:15	0.035156	3.4	0	0	0	0	0	999	999
02/11/2024 21:30	0.144531	3.1	0	0	0	0	0	999	999
02/11/2024 21:45	0.144531	3.4	0	0	0	0	0	999	999
02/11/2024 22:00	0.363281	3.3	0	0	0	0	0	999	999
02/11/2024 22:15	0.636719	3.2	0	0	0	0	0	999	999
02/11/2024 22:30	0.855469	3.3	0	0	0	0	0	999	999
02/11/2024 22:45	0.964844	3.1	0	0	0	0	0	999	999
02/11/2024 23:00	0.996094	3.2	0	0	0	0	0	999	999
02/11/2024 23:15	0.996094	3.1	0	0	0	0	0	999	999
02/11/2024 23:30	0.996094	3.4	0	0	0	0	0	999	999
02/11/2024 23:45	1	3.2	0	0	0	1	0	999	999
03/11/2024 00:00	1	3.2	0	0	0	0	0	999	999
03/11/2024 00:15	1	3.4	0	0	0	0	0	999	999
03/11/2024 00:30	1	3.3	0	0	0	0	0	999	999
03/11/2024 00:45	1	3.3	0	0	0	0	0	999	999
03/11/2024 01:00	1	3.3	0	0	0	0	0	999	999
03/11/2024 01:15	1	3	0	0	0	0	0	999	999
03/11/2024 01:30	1	3.2	0	0	0	0	1	999	999
03/11/2024 01:45	0.003906	3.2	0	0	0	0	0	999	999
03/11/2024 02:00	0.003906	3.3	0	0	0	0	0	999	999
03/11/2024 02:15	0.003906	3.5	0	0	0	0	0	999	999
03/11/2024 02:30	0.035156	3.3	0	0	0	0	0	999	999
03/11/2024 02:45	0.035156	3.1	0	0	0	0	0	999	999
03/11/2024 03:00	0.035156	3.2	0	0	0	0	0	999	999
03/11/2024 03:15	0.035156	3.1	0	0	0	0	0	999	999
03/11/2024 03:30	0.035156	3.3	0	0	0	0	0	999	999
03/11/2024 03:45	0.035156	3.2	0	0	0	0	0	999	999
03/11/2024 04:00	0.144531	3.2	0	0	0	0	0	999	999
03/11/2024 04:15	0.363281	3.4	0	0	0	0	0	999	999
03/11/2024 04:30	0.363281	3.3	0	0	0	0	0	999	999

03/11/2024 04:45	0.636719	3.4	0	0	0	0	0	999	999
03/11/2024 05:00	0.855469	3.1	0	0	0	0	0	999	999
03/11/2024 05:15	0.964844	3.2	0	0	0	0	0	999	999
03/11/2024 05:30	0.996094	3.2	0	0	0	0	0	999	999
03/11/2024 05:45	1	3.2	0	0	0	1	0	999	999
03/11/2024 06:00	1	3.1	0	0	0	0	0	999	999
03/11/2024 06:15	1	3.2	0	0	0	0	0	999	999
03/11/2024 06:30	1	3	0	0	0	0	0	999	999
03/11/2024 06:45	1	3.1	0	0	0	0	0	999	999
03/11/2024 07:00	1	3.2	0	0	0	0	0	999	999
03/11/2024 07:15	1	3.2	0	0	0	0	0	999	999
03/11/2024 07:30	1	3.2	0	0	0	0	0	999	999
03/11/2024 07:45	1	3.2	0	0	0	0	0	999	999
03/11/2024 08:00	1	2.9	0	0	0	0	1	999	999
03/11/2024 08:15	0.035156	3.2	0	0	0	0	0	999	999
03/11/2024 08:30	0.035156	3.2	0	0	0	0	0	999	999
03/11/2024 08:45	0.035156	3.2	0	0	0	0	0	999	999
03/11/2024 09:00	0.035156	2.9	0	0	0	0	0	999	999
03/11/2024 09:15	0.144531	3.2	0	0	0	0	0	999	999
03/11/2024 09:30	0.144531	2.9	0	0	0	0	0	999	999
03/11/2024 09:45	0.363281	3	0	0	0	0	0	999	999
03/11/2024 10:00	0.363281	3	0	0	0	0	0	999	999
03/11/2024 10:15	0.144531	3.1	0	0	0	0	0	999	999
03/11/2024 10:30	0.144531	3.2	0	0	0	0	0	999	999
03/11/2024 10:45	0.144531	3	0	0	0	0	0	999	999
03/11/2024 11:00	0.144531	3.2	0	0	0	0	0	999	999
03/11/2024 11:15	0.035156	3.1	0	0	0	0	0	999	999
03/11/2024 11:30	0.035156	3.1	0	0	0	0	0	999	999
03/11/2024 11:45	0.003906	3	0	0	0	0	0	999	999
03/11/2024 12:00	0.003906	3.2	0	0	0	0	0	999	999
03/11/2024 12:15	0.003906	3.1	0	0	0	0	0	999	999
03/11/2024 12:30	0.003906	3.1	0	0	0	0	0	999	999
03/11/2024 12:45	0.003906	3.1	0	0	0	0	0	999	999
03/11/2024 13:00	0.035156	3.1	0	0	0	0	0	999	999
03/11/2024 13:15	0.144531	3.1	0	0	0	0	0	999	999
03/11/2024 13:30	0.363281	3.1	0	0	0	0	0	999	999

03/11/2024 13:45	0.636719	3.3	0	0	0	0	0	999	999
03/11/2024 14:00	0.855469	3	0	0	0	0	0	999	999
03/11/2024 14:15	0.964844	3.1	0	0	0	0	0	999	999
03/11/2024 14:30	0.996094	3.1	0	0	0	0	0	999	999
03/11/2024 14:45	1	3.2	0	0	0	1	0	999	999
03/11/2024 15:00	1	3.2	0	0	0	0	0	999	999
03/11/2024 15:15	1	3.2	0	0	0	0	0	999	999
03/11/2024 15:30	1	3.2	0	0	0	0	0	999	999
03/11/2024 15:45	1	3.1	0	0	0	0	0	999	999
03/11/2024 16:00	1	3	0	0	0	0	0	999	999
03/11/2024 16:15	1	3.1	0	0	0	0	0	999	999
03/11/2024 16:30	1	3.1	0	0	0	0	0	999	999
03/11/2024 16:45	1	3	0	0	0	0	0	999	999
03/11/2024 17:00	1	3.1	0	0	0	0	1	999	999
03/11/2024 17:15	0.003906	2.9	0	0	0	0	0	999	999
03/11/2024 17:30	0.003906	3	0	0	0	0	0	999	999
03/11/2024 17:45	0.003906	3.1	0	0	0	0	0	999	999
03/11/2024 18:00	0.035156	3.3	0	0	0	0	0	999	999
03/11/2024 18:15	0.144531	3.3	0	0	0	0	0	999	999
03/11/2024 18:30	0.363281	3.1	0	0	0	0	0	999	999
03/11/2024 18:45	0.636719	2.9	0	0	0	0	0	999	999
03/11/2024 19:00	0.855469	3.2	0	0	0	0	0	999	999
03/11/2024 19:15	0.964844	3.2	0	0	0	0	0	999	999
03/11/2024 19:30	0.996094	3.1	0	0	0	0	0	999	999
03/11/2024 19:45	1	3.2	0	0	0	1	0	999	999
03/11/2024 20:00	1	3	0	0	0	0	0	999	999
03/11/2024 20:15	1	3.8	0	0	0	0	0	999	999
03/11/2024 20:30	1	3.3	0	0	0	0	0	999	999
03/11/2024 20:45	1	3.3	0	0	0	0	0	999	999
03/11/2024 21:00	1	3.1	0	0	0	0	0	999	999
03/11/2024 21:15	1	3.3	0	0	0	0	0	999	999
03/11/2024 21:30	1	3.2	0	0	0	0	0	999	999
03/11/2024 21:45	1	3.2	0	0	0	0	0	999	999
03/11/2024 22:00	1	3.3	0	0	0	0	1	999	999
03/11/2024 22:15	0.003906	3.2	0	0	0	0	0	999	999
03/11/2024 22:30	0.003906	3.4	0	0	0	0	0	999	999

03/11/2024 22:45	0.035156	3.1	0	0	0	0	0	999	999
03/11/2024 23:00	0.144531	3.2	0	0	0	0	0	999	999
03/11/2024 23:15	0.363281	3.3	0	0	0	0	0	999	999
03/11/2024 23:30	0.636719	3.1	0	0	0	0	0	999	999
03/11/2024 23:45	0.855469	3.3	0	0	0	0	0	999	999
04/11/2024 00:00	0.964844	3.2	0	0	0	0	0	999	999
04/11/2024 00:15	0.996094	3.3	0	0	0	0	0	999	999
04/11/2024 00:30	1	3.1	0	0	0	1	0	999	999
04/11/2024 00:45	1	3.2	0	0	0	0	0	999	999
04/11/2024 01:00	1	3.6	0	0	0	0	0	999	999
04/11/2024 01:15	1	3.4	0	0	0	0	0	999	999
04/11/2024 01:30	1	3.1	0	0	0	0	0	999	999
04/11/2024 01:45	1	3.2	0	0	0	0	0	999	999
04/11/2024 02:00	1	3.2	0	0	0	0	0	999	999
04/11/2024 02:15	1	3.5	0	0	0	0	0	999	999
04/11/2024 02:30	1	3.2	0	0	0	0	0	999	999
04/11/2024 02:45	1	3.4	0	0	0	0	1	999	999
04/11/2024 03:00	0.003906	3.4	0	0	0	0	0	999	999
04/11/2024 03:15	0.003906	3.1	0	0	0	0	0	999	999
04/11/2024 03:30	0.003906	3.4	0	0	0	0	0	999	999
04/11/2024 03:45	0.003906	3.4	0	0	0	0	0	999	999
04/11/2024 04:00	0.003906	3.2	0	0	0	0	0	999	999
04/11/2024 04:15	0.003906	3.2	0	0	0	0	0	999	999
04/11/2024 04:30	0.003906	3.2	0	0	0	0	0	999	999
04/11/2024 04:45	0.003906	3.3	0	0	0	0	0	999	999
04/11/2024 05:00	0.003906	3.4	0	0	0	0	0	999	999
04/11/2024 05:15	0.003906	3.1	0	0	0	0	0	999	999
04/11/2024 05:30	0.003906	3.2	0	0	0	0	0	999	999
04/11/2024 05:45	0.003906	3.4	0	0	0	0	0	999	999
04/11/2024 06:00	0.003906	3.2	0	0	0	0	0	999	999
04/11/2024 06:15	0.003906	3	0	0	0	0	0	999	999
04/11/2024 06:30	0.035156	3.4	0	0	0	0	0	999	999
04/11/2024 06:45	0.035156	3.3	0	0	0	0	0	999	999
04/11/2024 07:00	0.035156	3.1	0	0	0	0	0	999	999
04/11/2024 07:15	0.035156	3.1	0	0	0	0	0	999	999
04/11/2024 07:30	0.035156	3.1	0	0	0	0	0	999	999

04/11/2024 07:45	0.035156	3.1	0	0	0	0	0	999	999
04/11/2024 08:00	0.035156	3.7	0	0	0	0	0	999	999
04/11/2024 08:15	0.144531	3.1	0	0	0	0	0	999	999
04/11/2024 08:30	0.035156	3.2	0	0	0	0	0	999	999
04/11/2024 08:45	0.035156	2.9	0	0	0	0	0	999	999
04/11/2024 09:00	0.144531	3.2	0	0	0	0	0	999	999
04/11/2024 09:15	0.144531	3.1	0	0	0	0	0	999	999
04/11/2024 09:30	0.144531	3.2	0	0	0	0	0	999	999
04/11/2024 09:45	0.144531	3.1	0	0	0	0	0	999	999
04/11/2024 10:00	0.363281	3.2	0	0	0	0	0	999	999
04/11/2024 10:15	0.363281	3.1	0	0	0	0	0	999	999
04/11/2024 10:30	0.636719	3.1	0	0	0	0	0	999	999
04/11/2024 10:45	0.855469	3.2	0	0	0	0	0	999	999
04/11/2024 11:00	0.855469	3.8	0	0	0	0	0	999	999
04/11/2024 11:15	0.964844	3.4	0	0	0	0	0	999	999
04/11/2024 11:30	0.996094	3.2	0	0	0	0	0	999	999
04/11/2024 11:45	1	3.3	0	0	0	1	0	999	999
04/11/2024 12:00	1	3.5	0	0	0	0	0	999	999
04/11/2024 12:15	1	3.7	0	0	0	0	0	999	999
04/11/2024 12:30	1	3.2	0	0	0	0	0	999	999
04/11/2024 12:45	1	3.4	0	0	0	0	0	999	999
04/11/2024 13:00	1	3.1	0	0	0	0	0	999	999
04/11/2024 13:15	1	3.2	0	0	0	0	0	999	999
04/11/2024 13:30	1	3.2	0	0	0	0	0	999	999
04/11/2024 13:45	1	3.2	0	0	0	0	0	999	999
04/11/2024 14:00	1	3.2	0	0	0	0	1	999	999
04/11/2024 14:15	0.035156	3.2	0	0	0	0	0	999	999
04/11/2024 14:30	0.144531	3	0	0	0	0	0	999	999
04/11/2024 14:45	0.363281	3.2	0	0	0	0	0	999	999
04/11/2024 15:00	0.636719	3.3	0	0	0	0	0	999	999
04/11/2024 15:15	0.855469	3.3	0	0	0	0	0	999	999
04/11/2024 15:30	0.964844	3.2	0	0	0	0	0	999	999
04/11/2024 15:45	0.996094	3.3	0	0	0	0	0	999	999
04/11/2024 16:00	1	3.4	0	0	0	1	0	999	999
04/11/2024 16:15	1	3.3	0	0	0	0	0	999	999
04/11/2024 16:30	1	3.5	0	0	0	0	0	999	999

04/11/2024 16:45	1	3.4	0	0	0	0	0	999	999
04/11/2024 17:00	1	3.2	0	0	0	0	0	999	999
04/11/2024 17:15	1	3.2	0	0	0	0	0	999	999
04/11/2024 17:30	1	3.2	0	0	0	0	0	999	999
04/11/2024 17:45	1	3.2	0	0	0	0	0	999	999
04/11/2024 18:00	1	3.2	0	0	0	0	0	999	999
04/11/2024 18:15	1	2.9	0	0	0	0	1	999	999
04/11/2024 18:30	0.035156	3.4	0	0	0	0	0	999	999
04/11/2024 18:45	0.144531	3.2	0	0	0	0	0	999	999
04/11/2024 19:00	0.144531	3.3	0	0	0	0	0	999	999
04/11/2024 19:15	0.363281	3.3	0	0	0	0	0	999	999
04/11/2024 19:30	0.636719	3.3	0	0	0	0	0	999	999
04/11/2024 19:45	0.855469	3.2	0	0	0	0	0	999	999
04/11/2024 20:00	0.964844	3.4	0	0	0	0	0	999	999
04/11/2024 20:15	0.996094	3.4	0	0	0	0	0	999	999
04/11/2024 20:30	0.996094	3.3	0	0	0	0	0	999	999
04/11/2024 20:45	0.996094	3.2	0	0	0	0	0	999	999
04/11/2024 21:00	1	3.4	0	0	0	1	0	999	999
04/11/2024 21:15	1	3.3	0	0	0	0	0	999	999
04/11/2024 21:30	1	3.2	0	0	0	0	0	999	999
04/11/2024 21:45	1	3.3	0	0	0	0	0	999	999
04/11/2024 22:00	1	3.2	0	0	0	0	0	999	999
04/11/2024 22:15	1	3.4	0	0	0	0	0	999	999
04/11/2024 22:30	1	3.3	0	0	0	0	0	999	999
04/11/2024 22:45	1	4	0	0	0	0	1	999	999
04/11/2024 23:00	0.035156	3.4	0	0	0	0	0	999	999
04/11/2024 23:15	0.035156	3.3	0	0	0	0	0	999	999
04/11/2024 23:30	0.035156	3.1	0	0	0	0	0	999	999
04/11/2024 23:45	0.035156	3	0	0	0	0	0	999	999
05/11/2024 00:00	0.144531	2.9	0	0	0	0	0	999	999
05/11/2024 00:15	0.363281	3	0	0	0	0	0	999	999
05/11/2024 00:30	0.636719	2.9	0	0	0	0	0	999	999
05/11/2024 00:45	0.855469	3.1	0	0	0	0	0	999	999
05/11/2024 01:00	0.855469	3.1	0	0	0	0	0	999	999
05/11/2024 01:15	0.964844	3.1	0	0	0	0	0	999	999
05/11/2024 01:30	0.996094	2.9	0	0	0	0	0	999	999

05/11/2024 01:45	1	2.9	0	0	0	1	0	999	999
05/11/2024 02:00	1	3	0	0	0	0	0	999	999
05/11/2024 02:15	1	3.2	0	0	0	0	0	999	999
05/11/2024 02:30	1	3	0	0	0	0	0	999	999
05/11/2024 02:45	1	3	0	0	0	0	0	999	999
05/11/2024 03:00	1	3.1	0	0	0	0	0	999	999
05/11/2024 03:15	1	2.8	0	0	0	0	0	999	999
05/11/2024 03:30	1	3.1	0	0	0	0	0	999	999
05/11/2024 03:45	1	3.1	0	0	0	0	0	999	999
05/11/2024 04:00	1	3.1	0	0	0	0	1	999	999
05/11/2024 04:15	0.035156	3	0	0	0	0	0	999	999
05/11/2024 04:30	0.144531	3.1	0	0	0	0	0	999	999
05/11/2024 04:45	0.363281	3.1	0	0	0	0	0	999	999
05/11/2024 05:00	0.636719	3	0	0	0	0	0	999	999
05/11/2024 05:15	0.855469	2.8	0	0	0	0	0	999	999
05/11/2024 05:30	0.964844	3.1	0	0	0	0	0	999	999
05/11/2024 05:45	0.996094	3.1	0	0	0	0	0	999	999
05/11/2024 06:00	1	2.8	0	0	0	1	0	999	999
05/11/2024 06:15	1	2.9	0	0	0	0	0	999	999
05/11/2024 06:30	1	2.8	0	0	0	0	0	999	999
05/11/2024 06:45	1	2.8	0	0	0	0	0	999	999
05/11/2024 07:00	1	2.9	0	0	0	0	0	999	999
05/11/2024 07:15	1	3.4	0	0	0	0	0	999	999
05/11/2024 07:30	1	3	0	0	0	0	0	999	999
05/11/2024 07:45	1	2.9	0	0	0	0	0	999	999
05/11/2024 08:00	1	2.9	0	0	0	0	0	999	999
05/11/2024 08:15	1	3.2	0	0	0	0	1	999	999
05/11/2024 08:30	0.003906	3.1	0	0	0	0	0	999	999
05/11/2024 08:45	0.003906	3.1	0	0	0	0	0	999	999
05/11/2024 09:00	0.035156	2.8	0	0	0	0	0	999	999
05/11/2024 09:15	0.144531	2.9	0	0	0	0	0	999	999
05/11/2024 09:30	0.363281	2.9	0	0	0	0	0	999	999
05/11/2024 09:45	0.636719	2.9	0	0	0	0	0	999	999
05/11/2024 10:00	0.855469	2.9	0	0	0	0	0	999	999
05/11/2024 10:15	0.964844	2.9	0	0	0	0	0	999	999
05/11/2024 10:30	0.996094	2.9	0	0	0	0	0	999	999

05/11/2024 10:45	1	3.1	0	0	0	1	0	999	999
05/11/2024 11:00	1	3.1	0	0	0	0	0	999	999
05/11/2024 11:15	1	2.9	0	0	0	0	0	999	999
05/11/2024 11:30	1	3	0	0	0	0	1	999	999
0	0	0	0	0	0	0	0	999	999
