# Supplementary material for: An open-source workflow for identifying hydrodynamic water quality events in rivers by continuous water quality monitoring and time-series data processing using R and US EPA CANARY
Source: MethodsX. 2025 Jul 27;15:103538. doi: 10.1016/j.mex.2025.103538 (PMC12347471; doi:10.1016/j.mex.2025.103538)
Supplement: Supplementary file 1 — Supplementary material and/or additional information The supplementary material for this research includes a detailed step by step user guide to the workflow for importing the data, running the R code, and for the output of the analysis provided as Supplementary Files S1-S4. S1: User Guide - Timeseries Water Quality data processing with R and CANARY for Hydrodynamic Event Monitoring. S2: Details for CANARY Output Files S3: Logic for identifying full and partial matches between manually verified events and CANARY events. S4: Example of match logic applied to _Dat_For_OW061024_051124C_LPCF_BED6_ET0.89063alg_1. S5: Supplementary References for non-peer reviewed publications. The source code used for visualisation and data analysis is publicly available on GitHub (Cronin and Taylor, 2025) along with a README file describing how to reproduce the results. The repository contains data, an interactive R markdown file with code and results of analysis, so users can apply the workflow directly to their own datasets. The repository is structured as per the nine-step protocol for data visualization and training of the CANARY event detection system detailed in Fig. 5 and detailed in the user guide. All materials are freely accessible under the MIT license. [file mmc1.zip › S5 Supplementary References.docx]

**An open-source workflow for identifying hydrodynamic water quality events in rivers by continuous water quality monitoring and time-series data processing using R and US EPA CANARY**

**Supplementary References**

Auguie, B. and Antonov, A. (2017) ‘gridExtra: Miscellaneous Functions for “Grid” Graphics (Version 2.3)’. Comprehensive R Archive Network. Available at: https://cran.r-project.org/web/packages/gridExtra/index.html (Accessed: 16 June 2025).

Barrett, T. *et al.* (2024) ‘data.table: Extension of “data.frame” (Version 1.17.4)’. Available at: https://cran.r-project.org/web/packages/data.table/index.html (Accessed: 16 June 2025).

Burkhardt, J. (2024) Email to Lisa Cronin, 6 December.

Coonan, B., Curley, M. and Ryan, C. (2024) *Long-term rainfall averages for Ireland 1991-2020*. Climatological Note No. 22. Met Éireann, p. 32. Available at: https://www.tara.tcd.ie/handle/2262/108693 (Accessed: 16 June 2025).

Cronin, L. and Taylor, C.M. (2025) ‘Workflow for identifying water quality hydrodynamic events in time series data (V1.0.0)’. Zenodo. Available at: https://doi.org/10.5281/ZENODO.15358190 (Accessed: 2025-06-16).

EEA (2024) *Europe’s state of water 2024: the need for improved water resilience*. 07/2024. Copenhagen, Denmark: European Environment Agency, p. 108. Available at: https://www.eea.europa.eu/en/analysis/publications/europes-state-of-water-2024 (Accessed: 16 June 2025).

European Commission (2000) *Directive 2000/60/EC of the European Parliament and of the Council of 23 October 2000 establishing a framework for community action in the field of water policy*. Available at: https://eur-lex.europa.eu/legal-content/EN/TXT/?uri=CELEX:32000L0060 (Accessed: 16 June 2025).

Goodwillie, R., Buckley, P. and Douglas, C. (1992) *Owenmore River, Proposed Arterial Drainage Environmental Impact Assessment, Botanical and Ornithological Surveys.* Environmental Impact Assessment. National Parks & Wildlife Service, Office of Public Works, p. 201. Available at: https://www.bing.com/ck/a?!&&p=e677b8a9a92aea87JmltdHM9MTY4NzY1MTIwMCZpZ3VpZD0yYzQ2ZTFkYS1lNGY3LTZiOTQtMjhmNi1mM2MyZTU4ODZhZjImaW5zaWQ9NTQ1OA&ptn=3&hsh=3&fclid=2c46e1da-e4f7-6b94-28f6-f3c2e5886af2&psq=owenmore+catchment+environmental+impact+assessment&u=a1aHR0cHM6Ly93d3cubnB3cy5pZS9zaXRlcy9kZWZhdWx0L2ZpbGVzL3B1YmxpY2F0aW9ucy9wZGYvR29vZHdpbGxpZV8xOTkyX093ZW5tb3JlX1JpdmVyLnBkZiM6fjp0ZXh0PVRoaXMlMjByZXBvcnQlMjBpcyUyMHRoZSUyMG91dGNvbWUlMjBvZiUyMGElMjBjb250cmFjdCx3aG8lMjBhbHNvJTIwZ2F2ZSUyMHNvbWUlMjBhc3Npc3RhbmNlJTIwaW4lMjB0aGUlMjBmaWVsZC4&ntb=1 (Accessed: 16 June 2025).

Grolemund, G. and Wickham, H. (2011) ‘Dates and Times Made Easy with lubridate’, *Journal of Statistical Software*, 40, pp. 1–25. Available at: https://doi.org/10.18637/jss.v040.i03.

Hart, D. and Haxon, T. (2020) *CANARY-EDS (Continuous Analysis of Networked Array of Sensors for Event Detection)*, *github*. Available at: https://github.com/USEPA/CANARY (Accessed: 16 June 2025).

Hart, D.B. and McKenna, S.A. (2012) ‘User’s Manual for CANARY’. US EPA. Available at: https://nepis.epa.gov/Exe/ZyPURL.cgi?Dockey=P1005VE2.TXT (Accessed: 16 June 2025).

Herrera, J. *et al.* (2022) ‘SEMI-AUTOMATED PRODUCTION AND FILTERING OF SATELLITE DERIVED WATER QUALITY PARAMETERS’, *The International Archives of the Photogrammetry, Remote Sensing and Spatial Information Sciences*, XLIII-B3-2022, pp. 1019–1026. Available at: https://doi.org/10.5194/isprs-archives-XLIII-B3-2022-1019-2022.

Hlavac, M. (2022) ‘stargazer: Well-Formatted Regression and Summary Statistics Tables (Version 5.2.3)’. Available at: https://cran.r-project.org/web/packages/stargazer/index.html (Accessed: 16 June 2024).

Hoppe, K. (2023) *Continuous Monitoring Water Quality Standard Operating Procedures*. Standard Operating Procedure Revision No. 2. Augusta, Maine: State of Maine Department of Environmental Protection, p. 50. Available at: https://www.maine.gov/dep/water/monitoring/Continuous%20Monitoring%20SOP%202023.pdf (Accessed: 16 June 2025).

Kristensen, P. *et al.* (2018) *European waters -- Assessment of status and pressures 2018*. Copenhagen, Denmark: European Environment Agency (7/2018). Available at: https://www.eea.europa.eu/en/analysis/publications/state-of-water (Accessed: 16 June 2025).

Larmarange, J. *et al.* (2025) ‘labelled: Manipulating Labelled Data (Version 2.14.0)’. Available at: https://cran.r-project.org/web/packages/labelled/index.html (Accessed: 16 June 2025).

Müller, K. and Bryan, J. (2020) ‘here: A Simpler Way to Find Your Files (Version 1.0.1)’. Available at: https://cran.r-project.org/web/packages/here/index.html (Accessed: 16 June 2025).

Murrell, P. (2005) ‘R: The Grid Graphics Package (Version 0.5-1)’. Available at: https://cran.r-project.org/web/packages/gridGraphics/index.html (Accessed: 16 June 2025).

Nolan, P. (2015) *Ensemble of regional climate model projections for Ireland: 2008-FS-CC-m*. Johnstown Castle, Co. Wexford, Ireland: Environmental Protection Agency (EPA Research report, no. 159, Accessed 16/06/2025).

Pileggi, S. (2024) ‘Context-is-king’, in *What They Forgot To Teach You About R*. *Posit conf(2024)*, Seattle. Available at: https://posit.co/blog/talks-and-workshops-from-posit-conf-2024/ (Accessed: 16 June 2025).

Posit team (2024) ‘RStudio: Integrated Development Environment for R’. Boston, MA: Posit Software, PBC. Available at: https://posit.co/products/open-source/rstudio/ (Accessed: 16 June 2025).

R Core Team (2024) ‘R: A Language and Environment for Statistical Computing (v4.4.1 on Windows 11x64 (build 22631)’. Vienna, Austria: R Foundation for Statistical Computing. Available at: https://www.R-project.org/ (Accessed: 16 June 2025).

Sievert, C. (2020) *Interactive Web-Based Data Visualization with R, plotly, and shiny*. Florida: Chapman & Hall (The R Series). Available at: https://www.routledge.com/Interactive-Web-Based-Data-Visualization-with-R-plotly-and-shiny/Sievert/p/book/9781138331457 (Accessed: 16 June 2025).

Tierney, N. and Cook, D. (2023) ‘Expanding Tidy Data Principles to Facilitate Missing Data Exploration, Visualization and Assessment of Imputations’, *Journal of Statistical Software*, 105, pp. 1–31. Available at: https://doi.org/10.18637/jss.v105.i07.

Wagner, R. *et al.* (2006) *Guidelines and standard procedures for continuous water-quality monitors: Station operation, record computation, and data reporting*. Water-Resources Investigations Report Report 00-4252. Reston, Virginia: U.S. Geological Survey & U.S. Dept. of the Interior, p. 51. Available at: https://pubs.usgs.gov/publication/tm1D3 (Accessed: 16 June 2025).

Wickham, H. (2009) *ggplot2: Elegant Graphics for Data Analysis*. New York, NY: Springer. Available at: https://doi.org/10.1007/978-0-387-98141-3.

Wickham, H. *et al.* (2019) ‘Welcome to the Tidyverse (v2.0.0)’, *Journal of Open Source Software*, 4(43), p. 1686. Available at: https://doi.org/10.21105/joss.01686.

Wickham, H. *et al.* (2023) ‘dplyr: A Grammar of Data Manipulation (V 1.1.4)’. Available at: https://cran.r-project.org/web/packages/dplyr/index.html (Accessed: 16 June 2025).

Wickham, H., Pedersen, T.L., *et al.* (2024) ‘scales: Scale Functions for Visualization (V1.4.0)’. Available at: https://cran.r-project.org/web/packages/scales/index.html (Accessed: 16 June 2025).

Wickham, H., Vaughan, D., *et al.* (2024) ‘tidyr: Tidy Messy Data (V 1.3.1)’. Available at: https://cran.r-project.org/web/packages/tidyr/index.html (Accessed: 16 June 2025).

Wickham, H. and Grolemund, G. (2017) *R for Data Science: Import, Tidy, Transform, Visualize, and Model*. 1st edn. ‎ O’Reilly Media. Available at: https://www.amazon.co.uk/R-Data-Science-Garrett-Grolemund/dp/1491910399 (Accessed: 16 June 2025).

Wickham, H., Software and PBC (2023) ‘stringr: Simple, Consistent Wrappers for Common String Operations (V1.5.1)’. Available at: https://cran.r-project.org/web/packages/stringr/index.html (Accessed: 16 June 2025).

Wilke, C.O. (2024) ‘cowplot: Streamlined Plot Theme and Plot Annotations for “ggplot2” (V 1.1.3)’. Available at: https://cran.r-project.org/web/packages/cowplot/index.html (Accessed: 16 June 2025).
